# Identification and Validation of a PD-L1 Binding Peptide for Determination of PDL1 Expression in Tumors

**DOI:** 10.1038/s41598-017-10946-2

**Published:** 2017-10-20

**Authors:** Charles Caldwell, Cory E. Johnson, V. N. Balaji, Govardhan A. Balaji, Richard D. Hammer, Raghuraman Kannan

**Affiliations:** 1 Departments of Radiology, School of Medicine, University of Missouri, Columbia, MO 65212 USA; 2 Departments of Bioengineering, School of Medicine, University of Missouri, Columbia, MO 65212 USA; 3 Departments of Pathology, School of Medicine, University of Missouri, Columbia, MO 65212 USA; 4 Molark Enterprises Pvt. Ltd., R.M.V.Extension, Bangalore, 560094 India

## Abstract

Blocking the interaction between Programmed Death Ligand 1 (PD-L1) and its receptor, PD-1, is an effective method of treating many types of cancers. Certain tumors overexpress PD-L1, causing host immune cells that express PD-1 to bind PD-L1 and cease killing the tumor. Inhibition of PD-L1 and PD-1 binding can restore host immunity towards tumor killing, and many new drugs have been developed to target this interaction. Current methods of PD-L1 diagnosis have shown to vary based on the antibody, detection kit brand, antigen retrieval method, and clinically defined methods by the FDA. To refine detection of PD-L1, we have identified a peptide, RK-10, and used it to detect PD-L1 expressing tumors with immunohistochemistry or flow cytometry. Flow cytometry was performed on cell lines and patient tissues using a fluorescent peptide (RK-10-Cy5). Immunohistochemistry using a biotin-modified peptide (RK-10-Biotin) was tested against the FDA-approved SP263 clone on biopsied patient tissues. For this study, we evaluated specificity of RK-10 using IHC in over 200 patient tissues, including NSCLC and Hodgkin’s Lymphoma. RK-10 shows staining in the tumor regions of FFPE tissues where the SP263 kit does not. RK-10-Cy5 peptide also demonstrates PD-L1 detection in NSCLC, breast, squamous cell carcinoma, and melanoma.

## Introduction

**Figure 1:**
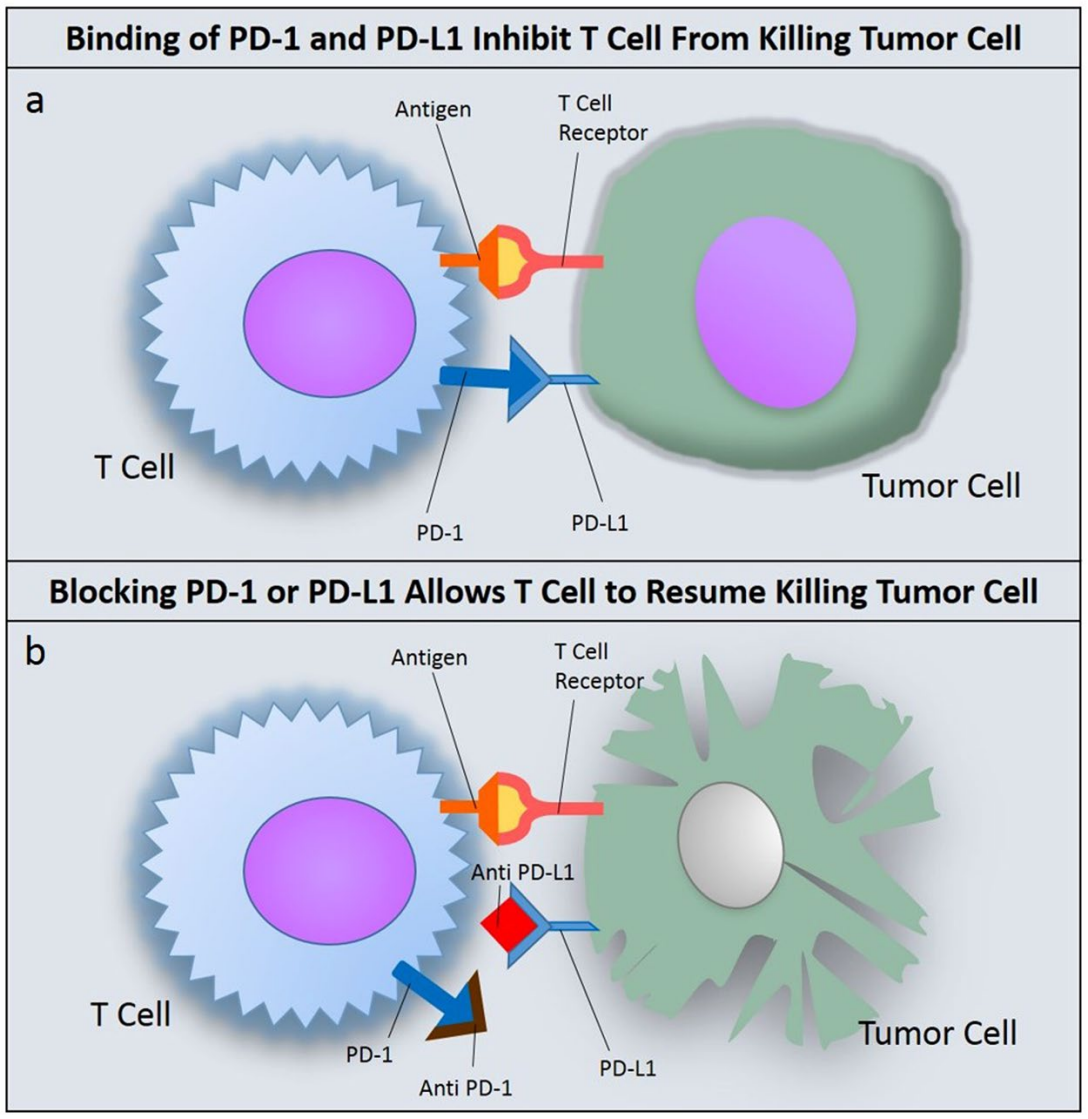
Blocking of PD-1 or PD-L1 Restores Host T-Cell Function. (**a**) Binding of PD-L1 to PD-1 will inhibit host T cell from killing the tumor cell; (**b**) By drugging either PD-1 or PD-L1, receptor binding is blocked and the T-cell can resume normal tumor killing functions.

Immune checkpoint inhibition has become an important modality for treating cancers, and has demonstrated significant success in recent years^1^. By inhibiting immune checkpoints host immune response recover from tumor evasion. The innate immune response can potentially negate the tumor’s ability to resist targeted therapy, eliminating the need for continuous lines of therapy^2^. There are numerous drugs either approved or in the pipeline that target dominant immune checkpoints such as PD-L1 or CTLA4^3,4^. One immune checkpoint of particular interest in human cancers is the interaction between Programmed Cell Death Receptor 1 (PD-1) and its ligand, Programmed Cell Death Ligand 1 (PD-L1)^5^. Overexpression of PD-L1 has been reported in many different tumor types, such as melanoma (40–100%), Non-Small Cell Lung Carcinoma (NSCLC) (35%-95%), Glioblastoma (100%), ovarian cancer (33–80%), and colorectal adenocarcinoma (53%)^6^. PD-L1 expression is characteristic of immune checkpoint evasion, allowing tumor cells to go unrecognized by immune T-cells as foreign. When an activated T-cell recognizes an antigen through binding of T-cell receptor to major histocompatibility complex, other checkpoints such as PD-1:PD-L1 are checked before the T-cell can recognize the cancer cell as foreign. When PD-1 on the T-cell surface and PD-L1 on the tumor surface are allowed to interact, the T-cell will be inhibited from destroying the foreign cell^7^ (Fig. 1). Many approved drugs are aimed at binding to and blocking either PD-1 or PD-L1 that stops receptor-ligand binding and will allow the T-cell to continue with killing foreign tumor cells. These drugs have shown therapeutic success in both primary and metastatic cancers^8,9^; however, not all patients will respond to this kind of therapy based on initial diagnosis. In order to determine which patients should be selected for immune checkpoint therapy, the appropriate diagnostic must be used to determine levels of PD-L1 in the tumor. Patient selection for the therapy depend on the levels of PD-L1 staining in the tissue. Above a certain “cutoff” point on staining pattern, patient would be considered as PD-L1 positive and expected to respond to administered therapy. Some clinical trials confirm that patients with higher expression of PD-L1 levels show increased response to the drug^10^. In other trials, it is shown that the expression is not a clear predictor for patient’s response^11^. Indeed, diagnosis of PD-L1 expression in patients has proven to be somewhat controversial due to proprietary methods and diagnostic interpretation^12,13^. PD-L1 assays are being developed in a ‘one drug – one assay’ method, where assay scoring and guidelines can vary based on the type of drug and diagnostic method used^14^, and companion diagnostic development is usually tied to the clinical outcome of the drug^15^. Drugs such as nivolumab, use PD-L1 companion assays for patient selection. Based on several clinical studies, it is clear that current immunohistochemistry (IHC) diagnostic agents for detecting PD-L1 in patients’ tissues suffer from three serious limitations. *First*, IHC agents for PD-L1 are based on antibodies raised against different clones of PD-L1; even though these IHC agents target the same marker they identify different parts of the marker. Therefore, these agents give different staining pattern based on the clone used. *Second*, the antibody used for detecting the primary IHC agent bound to the tissue would also be different in these assays resulting in varying performance based on the assay used for diagnosis. *Third*, the IHC agents were designed and developed by different companies and they would require the use of their own staining equipment and scoring algorithm. For example, Dako’s IHC agents used for selecting patients for nivolumab and pembrolizumab, utilize Dako IHC autostainer and their own scoring algorithm. In a similar fashion, for selecting patients for treating with drugs such as Atezolizumab and Durvalumab, Ventana diagnostics utilize Ventana automated IHC platforms and their own scoring algorithm. The data comparing these IHC agents for patients’ response, the Blueprint Project –a collaboration of 6 major pharmaceutical companies focused on comparing these tests with patient’s response data, is still ongoing. It is worth to mention here that factors such as tumor heterogeneity would not play a role in predicting patient response, as this factor is common in both PD-L1 positive and PD-L1 negative patients. Furthermore, running a different test for each drug evaluated is impractical due to limited tissue from biopsy, turnaround time, and cost. Potential harm to patients can result if inappropriate tests or cutoff levels are used to make treatment decisions^16^. Among all, the PD-L1 marker itself is also somewhat labile and must be evaluated soon after the biopsy^17^.

In this paper, we report the specificity of RK-10 in binding PD-L1 in cells, patient blood samples, and tissues. We have demonstrated that RK-10 binds to PD-L1 positive tumor cells -breast, retinoblastoma, and lung cancer cell lines using flow cytometry. Flow cytometry using RK-10 was also performed in whole blood spiked with PD-L1 expressing cells, and squamous cell carcinoma and metastatic melanoma obtained from patients. The specificity of RK-10 has been evaluated using IHC methods in over 200 different patient tissues. We used gold standard Ventana SP263 PD-L1 antibody for comparison.

## Materials and Methods

All experimental protocols under Evaluation of Molecular Mutations in Lung Cancer, IRB #2004603, were approved by the University of Missouri Institutional Review Board. All methods were carried out in accordance with relevant guidelines and regulations. All patient specimens were obtained from the tissue core and the samples were collected previously with informed consent.

### Identification of RK-10 Peptide [RK-10: GSGSGSTYLCGAISLAPKAQIKESL]

The RCSB protein data bank was searched for the complex of PD-1 and PD-L1. Out of the results, the structure corresponding to the PDB ID “4ZQK” was selected for analysis because it represents the structure of the complex of human programmed death-1 (PD-1) and its ligand PD-L1 in its non-mutated form with an X-ray resolution of 2.45 Å. The selected structure was visually examined using the open-source program PyMOL Molecular Graphics System Version 1.8.20. A proprietary Fortran program was developed and used to analyze interactions between residues within the binding region. If distance between two residues in the binding region was less than or equal to 1.2 times the sum of the Van der Waal’s radii of the two atoms, it was regarded to be a contact and the residue-residue contact count was updated to +1. Number of occurrences for each sequence was calculated and used to identify the peptide sequences used in this study.

### Flow Cytometry Using Cultured Cell Lines

Cell lines MDA-MB-231, Y79, and MCF-7 were purchased from ATCC, thawed, and grown in culture to confluency. When confluent, adherent cells were removed from the flask by scraping gently with a cell scraper and media removed using centrifugation. Suspension cells were pipetted from the flask and centrifuged to remove media. Cell lines were resuspended in Eppendorf tubes in 100 µL PBS at a concentration of 5 × 10^6^ cells per mL. Cy5-conjugated peptide solution was then added to the tubes to make the desired concentration of peptide in 200 µL. Eppendorf tubes were then placed in the incubator for 1 hour and vortexed at the 30-minute mark. After 1 hour, cell lines were analyzed on a BD FACS Canto II, a 3-laser, 8-color flow cytometer (San Jose, CA) using Diva 8.0 acquisition and analysis software (San Jose, CA). The cells of interest were gated using Forward and Side scatter (FSC/SSC) and positive antibody expression. 10,000 singlet events were collected for each specimen.

### Flow Cytometry Using Tissues

Cases were evaluated using flow cytometry for suspected hematopoietic neoplasms. A portion of each fresh specimen was collected into RPMI. Each sample was prepared to create cell suspensions which were combined with neat amounts of the following antibodies (BD, San Jose, CA): CD15 FITC, CD34 PE, CD33 PerCP-Cy5-5, CD13 PE-Cy7, CD11B APC, HLA-DR APC-H7, CD16 V450, CD45 V500C, Kappa FITC, Lambda PE, CD5 PerCP-Cy5-5, CD19 PE-Cy7, CD23 APC, CD20 APC-H7, CD10 BV421 V450, CD4 FITC, CD8 PE, CD2 PE-Cy7, CD56 APC, CD3 APC-H7, CD7 V450, CD38 PerCP-Cy5-5, CD10 APC, CD5 BV421 V450, CD23 PE, CD8 PE-Cy7, CD200 APC, and CD138 PerCP-Cy5-5 (Dako, Carpinteria, CA) and incubated for 15 minutes in the dark. Any erythrocytes within the specimens were lysed with BD PharmLyse (San Jose, CA) and the specimens were washed with BD Staining Buffer with BSA (San Jose, CA). Each sample was evaluated using BD FACSCanto II, a three laser, eight-color flow cytometer (San Jose, CA) within 24 hours of collection. 50,000 events were collected for each sample. The expression data were analyzed using BD FACSDiva software, version 8.0 (San Jose, CA). Cases diagnosed as non-hematopoietic tumors were further subjected to evaluation with PDL-1 peptide if material was available combined with CK to identify the epithelial component. The cell suspensions were stained with 10 µl of BD Cytokeratin FITC (clone CAM5.2), 20 µl of Cy5-conjugated peptide solution, and 10 µl of BD Pharmingen CD274 PE (clone MIH1), incubated in the dark for 30 minutes, washed with BD Stain Buffer with BSA, and reconstituted to 500 µl with Stain Buffer with BSA in 500 ml polystyrene tubes for analysis. The specimens were analyzed on the FACS Canto II using the same panel template, gating strategy, and collection events as the cell line specimens.

### Immunohistochemistry Using Biotinylated Peptide [Biotin-GSGSGSTYLCGAISLAPKAQIKESL]

To detect PD-L1 in FFPE tissues we employed manual IHC techniques with a biotin-conjugated version of peptide RK-10-Cy5 [Cy5-GSGSGSTYLCGAISLAPKAQIKESL] and compared with Ventana PD-L1 (SP263) Rabbit monoclonal Primary Antibody stained on a Roche Benchmark Ultra autostainer. Seven PD-L1 expressing NSCLC patient tissues were obtained from the MU OneHealth tissue bank and de-identified according to IRB protocols. Paraffin-embedded patient tissue slides were baked overnight, then de-waxed and rehydrated according to standard protocols. Tissue sections were then subjected to antigen retrieval in EDTA at 95 °C for 20 minutes in EDTA (pH 0.9). The solution is then cooled for an additional 20 minutes on the bench top prior to buffer rinse. Tissues were then incubated with 15 µM biotinylated peptide for 2 hours in a humid chamber at RT. After 2 hours, slides were washed with buffer and treated with Pierce™ High Sensitivity Streptavidin-HRP (1:200 dilution) (Sigma) for 30 minutes at RT in a humid chamber. Once this was complete, slides were again washed in buffer then treated with DAB (Sigma) for 10 minutes. Slides were again washed in buffer, then dehydrated using graded alcohol and xylene and counterstained with hematoxylin. Slides were then imaged using bright-field microscopy on a Leica DM5500.

### Immunohistochemistry Using Cy5-Peptide

We investigated PDL1 expression in the same seven FFPE tissues and 192 lung cancer cases on a microarray using our peptide conjugated with Cy5 fluorophore. A lung cancer tissue microarray (TMA) was purchased from U.S. Biomax that contains 192 separate cases of various types of lung cancers (LC1923, biomax.us). In addition, seven NSCLC patient tissues were obtained from the Mizzou OneHealth tissue bank and de-identified according to IRB protocols. Paraffin-embedded tissue slides were baked overnight, then de-waxed and rehydrated according to standard protocols. Tissue sections were then subjected to antigen retrieval in EDTA at 95 °C for 20 minutes in EDTA (pH 0.9). The solution is then cooled for an additional 20 minutes on the bench top prior to buffer rinse. Tissues were then incubated with 15 µM Cy5-conjugated peptide for 2 hours in a humid chamber in the dark at RT. After 2 hours, slides were washed with buffer. The slides were then mounted using nucleus-specific DAPI counterstain and cover slipped. Slides were then imaged using fluorescence microscopy on a Leica DM5500 and compared to the same sections which had been stained with the Ventana antibody. For fluorescent analysis, DAPI channels and Cy5 channels were overlaid to image cell nuclei and PD-L1 expression, respectively.

### IHC Blocking Using PD-L1 Peptide or Ventana Antibody SP263

To test specificity of the PD-L1 peptide, we first blocked the PD-L1 receptors on the tissue with RK-10 peptide for 1 hour prior to autostaining the tissue with SP263 antibody using the Roche autostainer with the Ventana PD-L1 kit according to Roche’s specifications. We also investigated blocking of the RK-10 peptide using the SP263 antibody by first treating the tissue with SP263 antibody for 30 minutes prior to treating the tissue with peptide according to the previously mentioned protocol.

## Results

### Identification of PD-L1 Binding Peptide and Mock Peptide (GSGSGSFVLNWYRMSPSNQTDKLAA)

**Figure 2:**
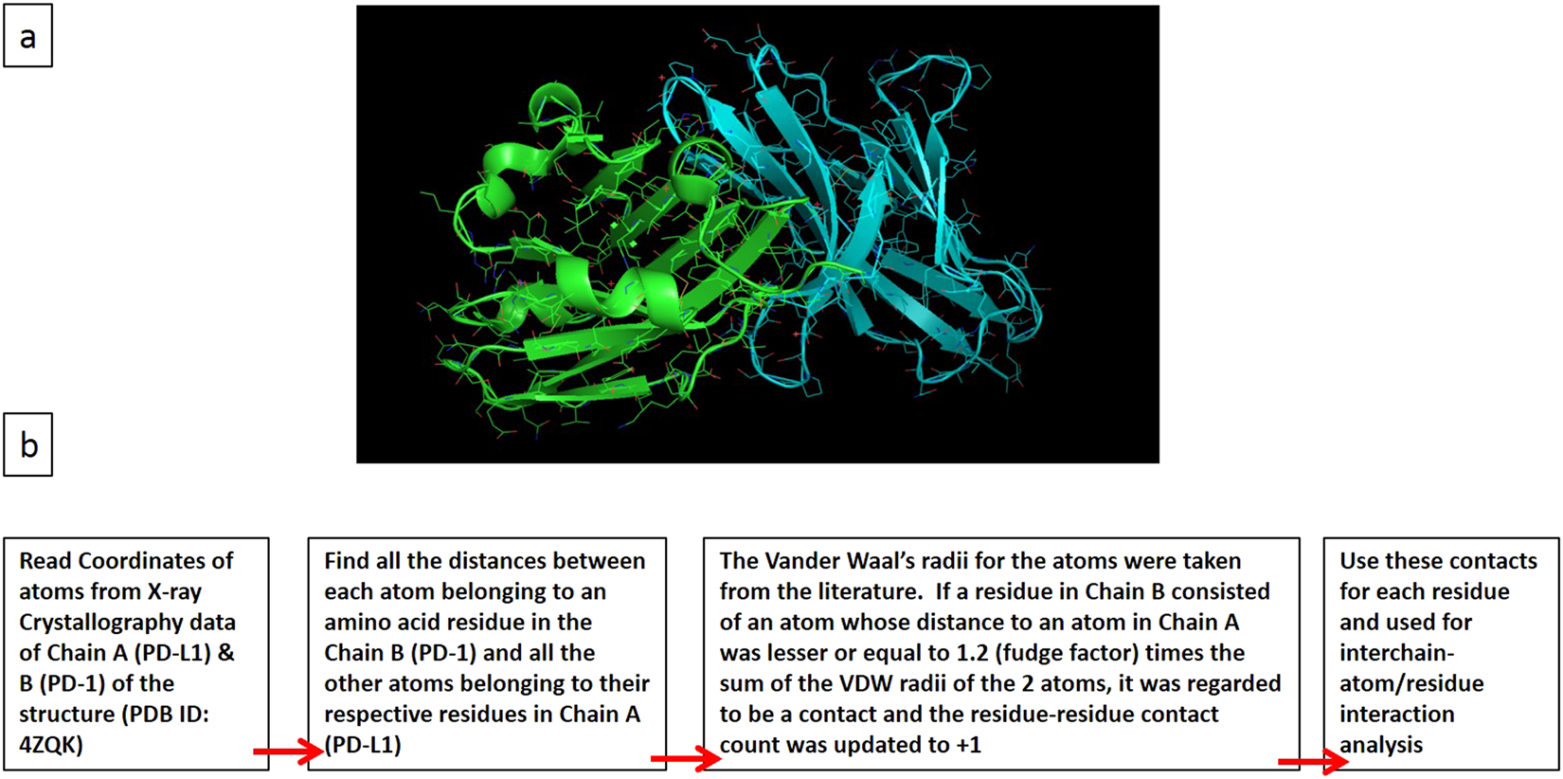
Protein Structures of PD-1 and PD-L1, with flow chart for identifying optimal binding sequence. (**a**) Protein structure of PD-1(blue) and PD-L1 (green); (**b**) Flowchart for identifying optimal peptide binding sequence.

We focused our initial research on understanding the interaction of PD-1 and PD-L1 based on X-ray crystal structure data (S1) with the goal of identifying the peptide sequence that is selectively mediating the interactions. After the crystal structure of each protein was identified, a proprietary Fortran program was used to analyze which amino acid sequences interact most closely between the two proteins. Number of occurrences for each sequence was calculated and used to identify the peptide sequences used in this study. (Fig. 2). The calculations provided several sequences of peptide that could possess high-affinity for targeting PD-L1 in tumor. As a first step, we synthesized a library of peptides and studied the stability and PD-L1 affinity. The study resulted in identification of a high affinity peptide, RK-10-Cy5 for targeting PD-L1. Data related to anti-PDL1 and mock peptide was used to synthesize peptides for this study. The binding sequences of each peptide were further modified to incorporate either biotin or fluorophore for antigen detection, and to increase solubility of the peptide.

### Fluorescent RK-10-Cy5 as Marker for Flow Cytometry

**Figure 3:**
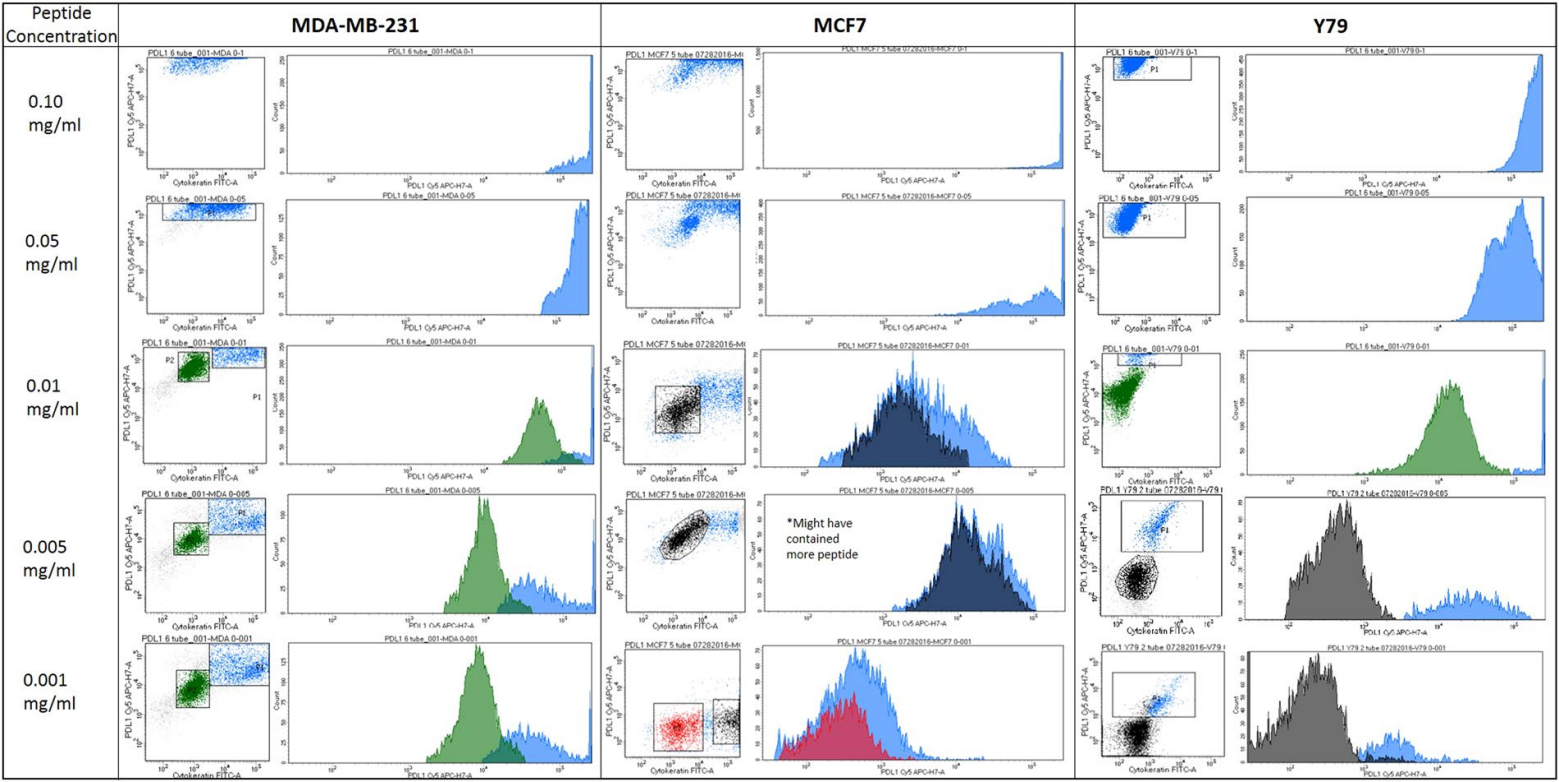
Titrations of RK-10-Cy5 in Cell Lines using flow cytometry. Cell lines expressing high PD-L1 (MDA-MB-231) and low PD-L1 (MCF7, Y79) were incubated with fluorescent RKC-10-Cy5 peptide and anti-cytokeratin for 1 hour at shown concentrations, then analyzed using flow cytometry. In some cases we can distinguish populations of PD-L1 expression, separated by color. Higher concentrations of peptide were unable to distinguish expression, while concentrations of 0.005 and 0.001 mg/mL peptide were selected as ideal for flow cytometry.

**Figure 4:**
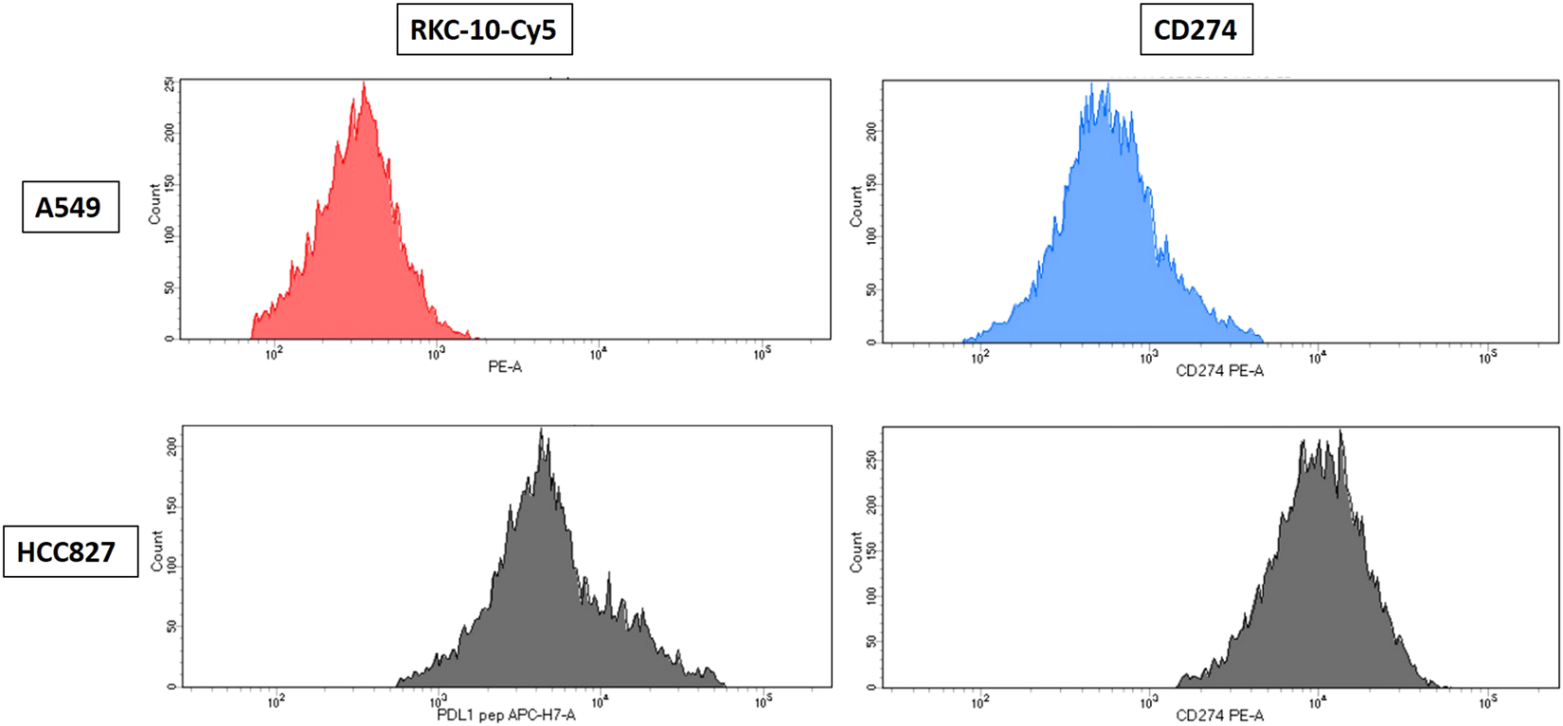
RK-10-Cy5 and CD274 antibody compared in NSCLC cell lines using flow cytometry. NSCLC cell lines A549 (PD-L1 low) and HCC827 (PD-L1 high) were incubated with either RKC-10-Cy5 peptide or CD274 antibody for 1 hour, then analyzed using flow cytometry. As expected, both antibody and peptide showed high MFI in HCC827, while A549 showed much lower MFI.

RK-10-Cy5 was then investigated for PD-L1 specificity using flow cytometry in cultured cell lines and patient tissues. The cell lines examined were breast cancer line MDA-MB-231, which shows very high PD-L1 expression^18^, along with retinoblastoma line Y79 and breast cancer line MCF-7, which show no meaningful PD-L1 expression^19^. To set our conditions for flow cytometry, we first examined titrations of PD-L1 peptide using all three cell lines. In each of the samples double-positives were selected by analyzing expression of both cytokeratin (FITC channel) and PD-L1 (Cy5 channel) (S2). All three cell lines showed a decrease in mean fluorescence intensity (MFI) as the concentration of peptide decreased (Figs 3 and S3–5). Samples containing 0.1 and 0.05 mg/mL concentrations were deemed to have fluorescence intensities too high for accurate analysis for each cell line. Y79 and MCF7 both have a much lower PD-L1 expression than MDA-MB-231, which correlates with expression seen using Cy5 conjugated peptide and comparing the cell lines with flow cytometry. Y79 and MCF7 MFI is close to tenfold lower than that seen in MDA-MB-231 in all lower concentrations. The MCF7 sample containing 0.005 mg/ml was much higher than anticipated due to this sample being treated twice with peptide. We selected 0.005 mg/mL as optimal concentration based on these comparisons, and all subsequent flow cytometry experiments were performed using this concentration. Lung cancer cell lines A549 (low PDL1) and HCC827 (high PDL1)^20^ were investigated for PD-L1 expression using both phycoerythrin-conjugated CD274 and RK-10-Cy5 (Figs 4 and S6). Cell lines were cultured as before and treated with either antibody or peptide in buffer. When run through the flow cytometer we notice a much higher signal associated with PD-L1 expression in the HCC827 cell line than in the A549 samples. The antibody associated fluorescence was higher than the peptide associated fluorescence in both samples, which is attributed to differences in titrating peptide and antibody.

### Detection of PD-L1 in Circulating Tumor Cells and Patient Tissues

**Figure 5:**
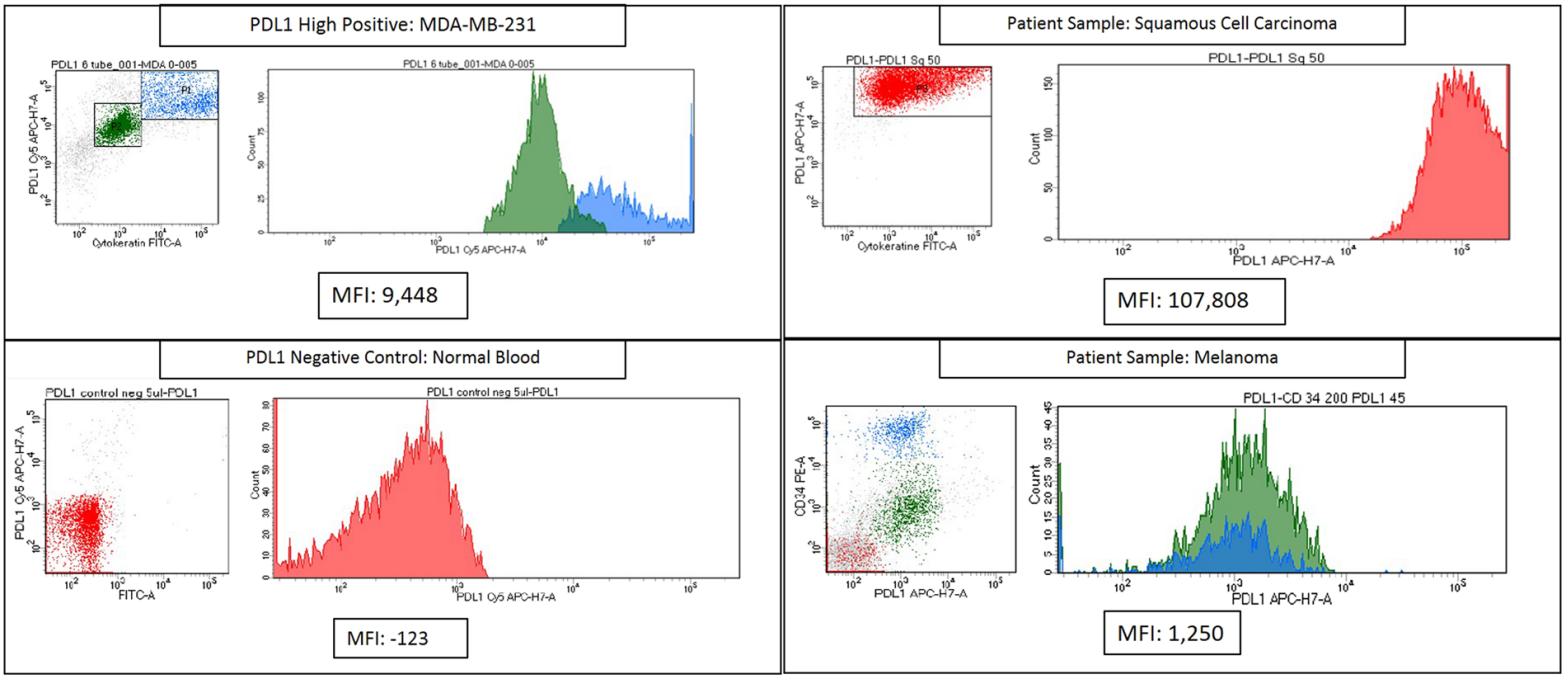
Flow cytometry analysis of PD-L1 expression in patient tissues using RK-10-Cy5. RKC-10-Cy5 peptide was incubated with patient melanoma and squamous cell carcinoma samples for 1 hour according to clinical protocols. Patient tissues were compared to MDA-MB-231 (PD-L1 high positive) and normal blood (PD-L1 negative) in order to asses PD-L1 expression. The SCC sample showed very high PD-L1 expression, while the melanoma sample showed a moderate expression of PD-L1.

**Figure 6:**
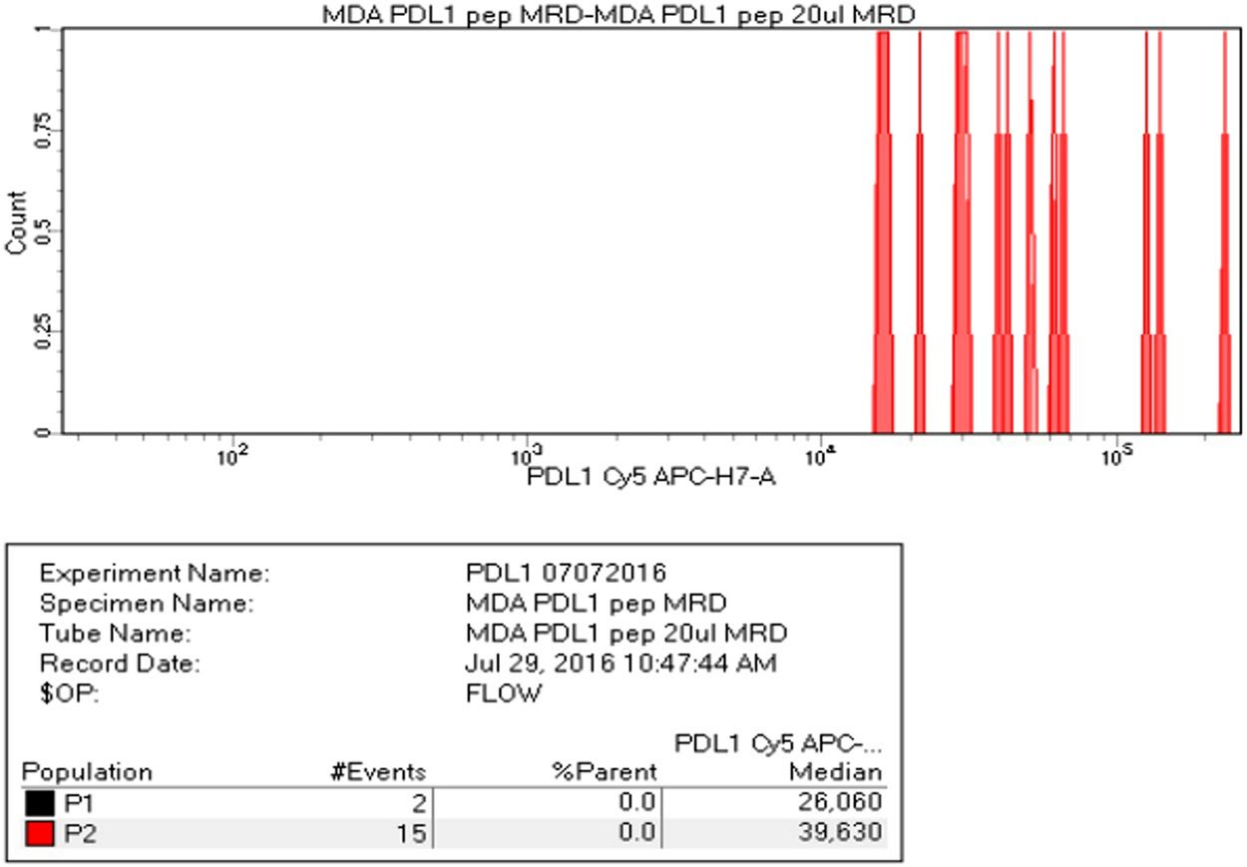
Flow cytometry analysis of PD-L1 expression in whole blood spiked with MDA-MB-231. High PD-L1 expressing cell line MDA-MB-231 was serially diluted in 2mL of whole blood and treated with RKC-10-Cy5 peptide and anti-cytokeratin. Counts of cells as low as 15 cells were seen using RKC-10-Cy5 peptide, and confirmed using the cytokeratin stain.

PD-L1 expression in patient tissues was analyzed using RK-10-Cy5 and compared to expression in MDA-MB-231 cells and a negative control of normal blood (Fig. 5). The MFI of the MDA-MB-231 cells was 9,448, while the whole blood gave an MFI of −123 (S9). Squamous cell carcinoma and metastatic melanoma samples were investigated for PD-L1 expression using the previously mentioned MFIs as high and negative, respectively. The squamous cell carcinoma was shown as having an MFI of 107,808, while the melanoma sample gave an MFI of 1,250. The squamous cell carcinoma PD-L1 expression was very high, while the melanoma sample was graded as ‘moderate’ PD-L1 expression (S10,11), and additionally showed no expression of cytokeratin. Since loss of CK expression is consistent with circulating tumor cells, it was thought that RK-10-Cy5 peptide could be used to detect CTCs. To see whether detection of low cell counts was possible, whole blood samples were spiked with MDA-MB-231 cells. MDA-MB-231 were diluted with whole blood, then treated with RK-10-Cy5 and cytokeratin before staining. These samples were also treated with a phycoerythrin-conjugated antibody (CD274) against PD-L1 to further verify PD-L1 detection. We were indeed able to detect positive signals of cytokeratin and PDL1 expression at low counts of ~15 cells in 1 mL whole blood sample using both the peptide and the antibody (Fig. 6).

### Biotinylated RK-10-Biotin Detects PD-L1 in NSCLC Patient Tissues

**Figure 7:**
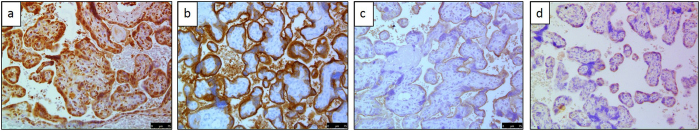
FFPE placenta tissue stained with RK-10-Biotin or SP263. Placenta tissue was treated with (**a**) 15 µM RKC-10-Biotin, (**b**) Ventana SP263 PDL-1 antibody, (**c**) pre-blocked with RKC-10-Biotin, then treated with Ventana PDL-1 antibody, (**d**) mock peptide RK-11-Biotin.

Seven Patient NSCLC tissues were investigated for PD-L1 using either manual IHC with 15 µM RK-10-Biotin peptide, or the Ventana PD-L1 (SP263) rabbit monoclonal primary antibody stained on a Roche Benchmark Ultra. In this study the peptide was conjugated with biotin, which was used to bind a secondary treatment of streptavidin-HRP. Formalin-Fixed Paraffin Embedded (FFPE) placenta tissue was used as the positive control, since PDL1 is expressed in placental trophoblasts^21^. In this study we also utilized biotinylated mock peptide RK-11-Biotin as a negative control. This mock peptide was synthesized to have very low affinity to PD-L1. Both the PD-L1 peptide and Ventana clone SP263 stained the trophoblasts heavily in the placental tissue (Figs 7 and S12–14). The SP263 antibody featured heavy edge staining but also showed membranous staining of the trophoblast cells, while the RK-10-Biotin peptide showed heavy membrane staining of the trophoblast cells without the intense edge artifacts seen when using the Ventana antibody. Mock peptide RK-11-Biotin showed light staining at high concentrations, but did not achieve the heavy trophoblast staining RK-10-Biotin did (S15). Higher concentrations of RK-10 peptide showed more staining in other parts of the placental tissue, but the heaviest staining is localized to the trophoblast cells (S2–S6). Blocking of the Ventana antibody was achieved by first treating the placenta tissue with RK-10-Biotin for 30 minutes, washing, and treating on the Roche autostainer according to specifications. The pre-blocked tissue showed drastic reduction of staining, with mostly edge artifacts being seen (Fig. 7c). Placenta tissue that was not pre-blocked was stained with the Ventana kit in parallel with the pre-blocked tissue, and showed the expected trophoblast staining as before (S16–18). As a negative control, we also stained normal lung, breast, and colorectal tissues using RK-10 and SP263. Each of these cases were determined as negative for PD-L1 expression (S19).

**Figure 8:**
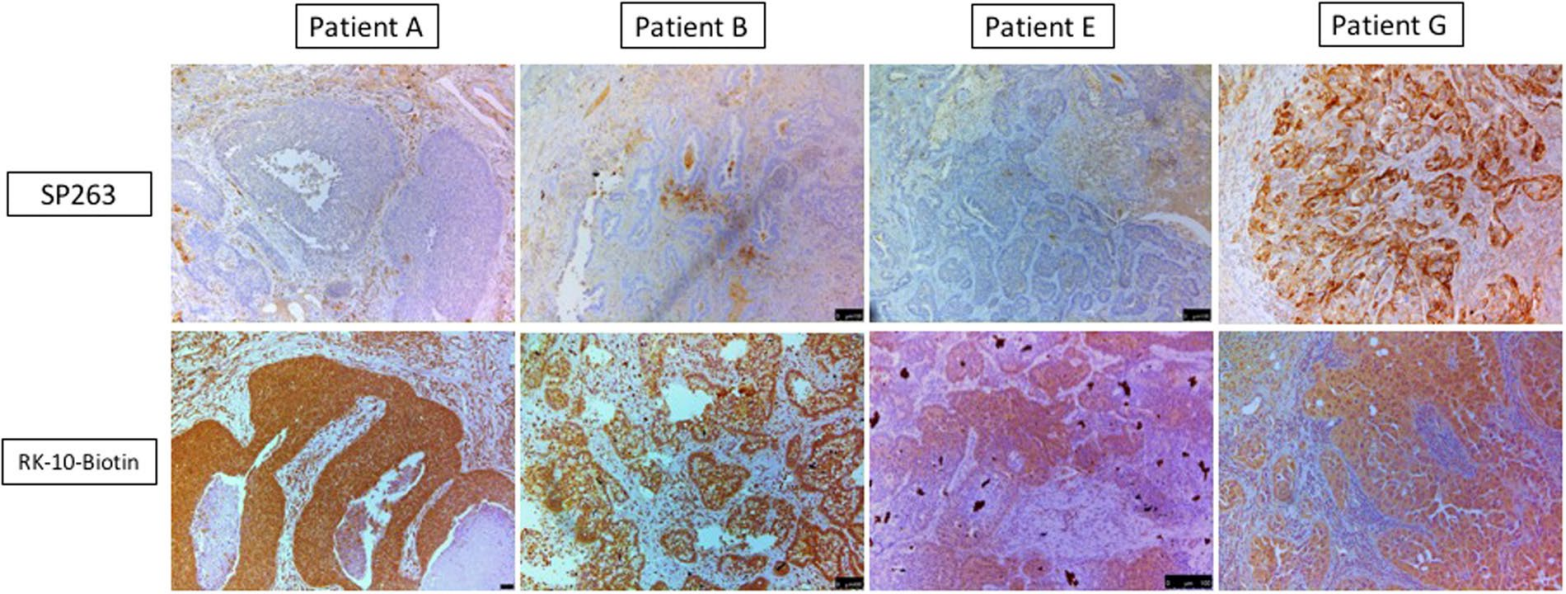
Patient tissues were stained using IHC protocols using either SP263 or RK-10-biotin and analyzed for tumor staining. RK-10-Biotin stains tumor specifically in all cases, whereas SP263 only showed significant staining in patient G.

Once confidence in PD-L1 staining was established in repeated placenta tissues, we compared staining in seven NSCLC patient tissues (patients ‘A’ through ‘G’) with the Ventana SP263 antibody and RK-10-Biotin (Figs 8 and S21–29 When using the RK-10-Biotin peptide we saw heaviest staining localized to the tumor regions of the tissues, which can be very intense based on the concentration of peptide used. In contrast, the SP263 antibody did not show heavy tumor staining in most tissue sections, showing only faint staining in these regions that could be interpreted as negative or faintly positive for PD-L1. The most intense staining from the SP263 antibody was shown in the tumor cells of patient G. All areas of patient G that stained positive for PD-L1 using the SP263 antibody also stained positively using PD-L1 peptide.

### Fluorescent RK-10-Cy5 Detects PD-L1 in NSCLC Patient Tissues and Tissue Microarray

**Figure 9:**
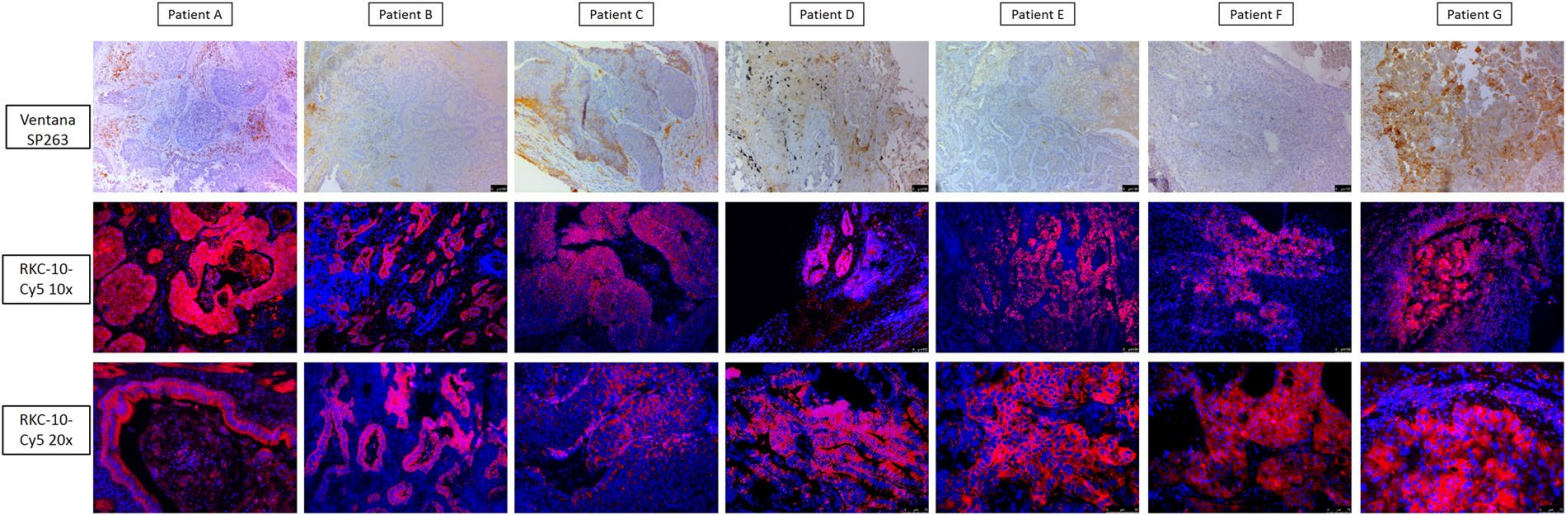
IHC detection of PD-L1 in seven patient tissues using fluorescent RK-10-cy5. Seven patient NSCLC tissues were stained using either SP263 or RKC-10-Cy5 peptide. All cases stained using RKC-10-Cy5 showed high affinity for tumor, while SP263 only showed heavy tumor staining in patient G and some lighter staining in patients C and D.

**Figure 10:**
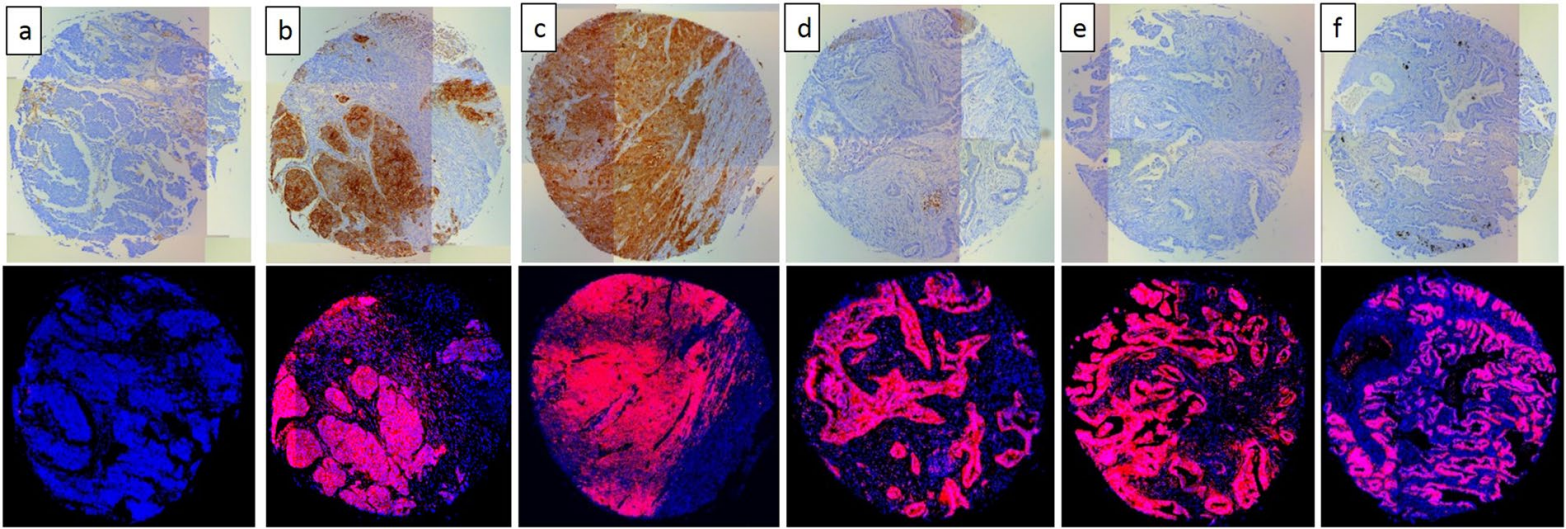
Select cases from NSCLC TMA stained with fluorescent RK-10-Cy5 or SP263. Six of 192 cores from Biomax TMA LC1923 are shown which were stained using either Roche SP263 antibody (top) or RKC-10-Cy5 peptide (bottom). Shown are representative samples which show negative PD-L1 with both methods (**a**), positive PD-L1 with both methods (**b**,**c**), and samples which are negative using SP263 but stain positive in the tumor using RKC-10-Cy5.

**Figure 11:**
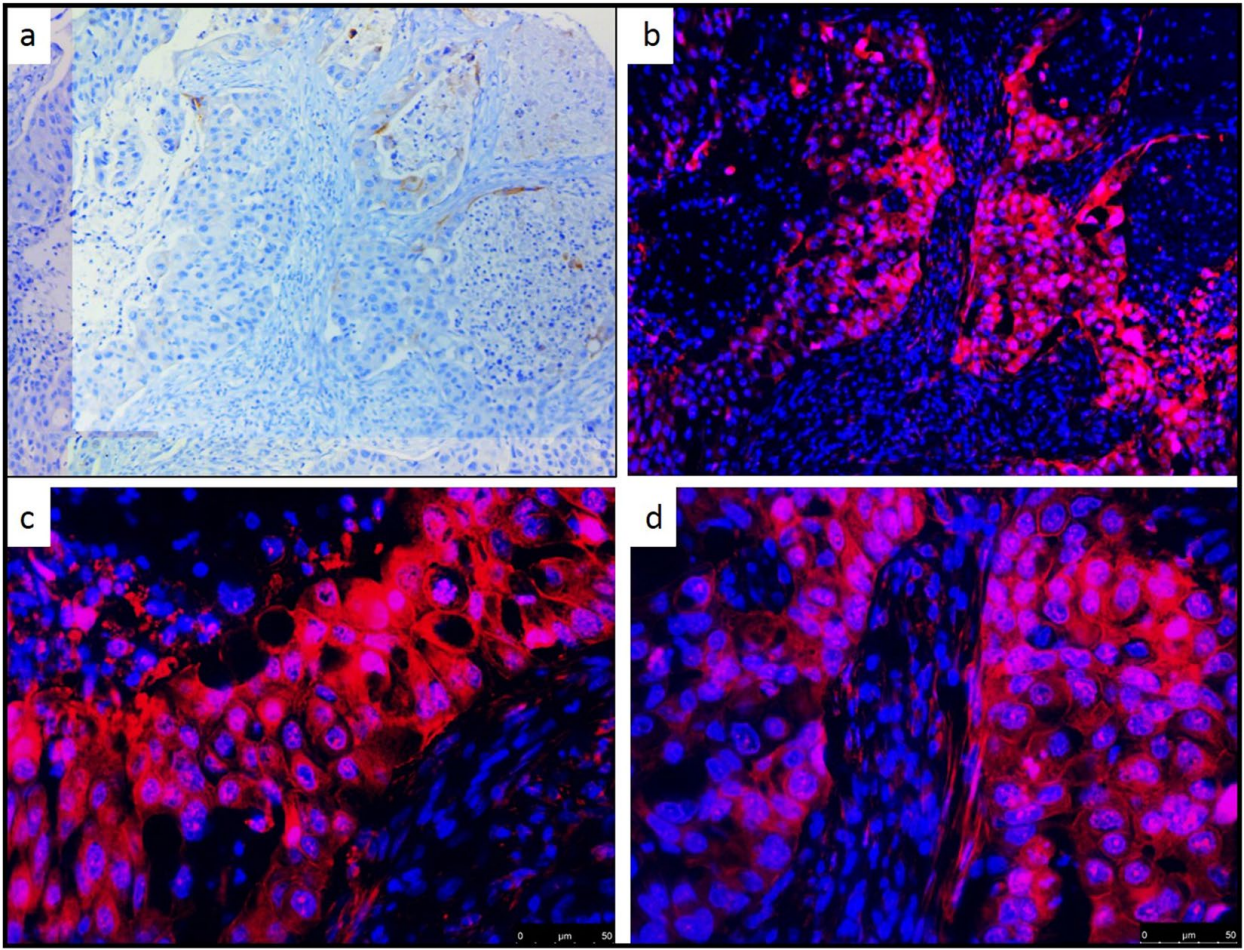
40x Magnification of fluorescent PD-L1 peptide stain. A selected TMA core which stained negative using SP264 antibody (**a**) shows high membrane staining in the tumor using fluorescent RKC-10-Cy5 peptide at 10x (**b**) and 40x (**c,d**) magnifications.

We again compared staining of the placenta and seven selected NSCLC patient tissues using the SP263 antibody and RK-10-Cy5 which was conjugated with a Cy5 fluorophore (S20). To stain with the fluorescent PD-L1 peptide, antigen retrieval was performed and tissue slides were treated with 15 µM fluorescent peptide in a dark, humid chamber for 2 hours, washed with buffer, then counter-stained and mounted with DAPI nucleus stain. Peptide-stained slides were imaged on a Leica DM5500 using channels specific for DAPI or Cy5, and channels were overlaid to examine PD-L1 expression. To confirm the data from the IHC stained tissues, the same seven patient tissues A-G were stained with RK-10-Cy5 (Figs 9 and S30–46). The Cy5 signal in these tissues was consistent with the HRP staining, where RK-10-Cy5 peptide stained many areas of tumor that the SP263 antibody did not. Where the SP263 staining is positive, we see similar staining between both the antibody and peptide. However, many tumor areas not visibly stained by the antibody were stained specifically when the peptide was used. To examine a larger range of tissues for PDL1 expression, fresh-cut lung cancer tissue microarrays containing 192 separate cases of lung cancers were purchased from U.S. Biomax, Inc. This array (S47) contained 78 cases of squamous cell carcinoma, 69 cases of adenocarcinoma, 3 cases of mucinous carcinoma, 7 cases of bronchioalveolar carcinoma, 5 cases of adenosquamous carcinoma, 4 cases of atypical carcinoid, 15 cases of small cell carcinoma, and 11 cases of large cell carcinoma. To analyze stained TMAs, the slides were scanned in at 10x magnification using the Leica DM5500 motorized stage and stitched together using Leica LAS X software (S48-56). Serial sectioned TMAs were then compared head to head when stained with either SP263 kit or the RK-10-Cy5 peptide (Fig. 10). In cases where the SP263 antibody was negative for tumor staining, the same is seen with the RK-10-Cy5 peptide (Fig. 10a). Likewise, in cases where the SP263 antibody stained positively in tumor, RK-10-Cy5 shows staining consistent with the SP263 stain (Fig. 10b,c). Interestingly, in the majority of cases, the SP263 antibody showed no tumor staining, while the RK-10-Cy5 peptide showed consistent, specific staining in tumor cells and immune infiltrate (Fig. 10d,e,f). Analysis of individual spots at 40x confirms the presence of specific tumor cell staining (Fig. 11). These results are consistent with the biotin-conjugated peptide IHC, where the PD-L1 peptide stained many large areas of tumor, while the SP263 antibody showed little to no staining in many of these areas. The Cy5 channel was very intensely bright and we had to use a very low exposure to image the PD-L1. PD-L1 expression was specifically seen in tumor areas of the tissue, and staining of immune cells was also seen outside the tumor areas, as is expected.

### Fluorescent RK-10-Cy5 Detects PD-L1 on Reed-Sternberg Cells in Hodgkin’s Lymphoma

**Figure 12:**
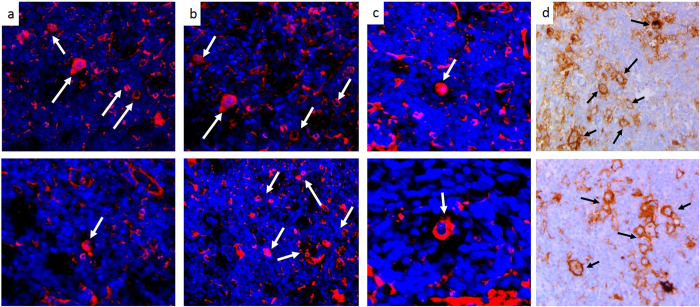
RK-10-Cy5 detects presence of PD-L1 on Reed-Sternberg cells in Hodgkin’s Lymphoma. Hodgkin’s Lymphoma tissues were incubated with either fluorescent RKC-10-Cy5 peptide (**a–c**) or SP263 antibody (**d**) to examine PD-L1 levels in Reed-Sternberg (RS) cells. RKC-10-Cy5 was able to specifically stain the RS cells (some but not all indicated by arrows), which are known to express high levels of PD-L1. PD-L1 expression in RS cells was also confirmed with the SP263 staining.

In addition to the NSCLC patient tissues, we also investigated four different Hodgkin’s Lymphoma cases for PD-L1 expression. Presence of Reed-Sternberg cells in a biopsied tissue is often the diagnostic indicator of a patient having Hodgkin’s lymphoma. RS cells are large, often multinucleated tumor cells that are derived from B-cell lymphocytes. RS cells heavily express PD-L1^22^, to the point of PD-L1 being a diagnostic indicator of RS cells. Due to the characteristic expression of PD-L1 in RS cells, we examined PD-L1 levels in the four identified Hodgkin’s lymphoma patient samples using the fluorescent RK-10-Cy5 peptide and compared with the SP263 antibody (Fig. 12). In each patient sample, the pathologist-identified RS cells showed PD-L1 staining with both RK-10-Cy5 and SP263 antibody. When using the RK-10-Cy5 peptide, RS cells were easily identified by the pathologist due to the heavy Cy5 fluorescent signal. These cells were additionally confirmed as RS cells by examining the multinucleate characteristic of the cells, shown clearly by staining the nuclei with fluorescent DAPI. The SP263 antibody IHC additionally confirmed the presence of PD-L1 in the RS cells. Using both methods, we also see some light staining of the tumor microenvironment besides the RS cells, which is expected as PD-L1 is often expressed on immune cells.

## Discussion

In order to overcome the problems associated with PD-L1 IHC, we have identified a novel peptide sequence, RK-10, which is specific for human PD-L1. RK-10 peptide sequence has shown to bind optimally to the structure of PD-1 receptor using crystal structure analysis of the PD-L1:PD-1 binding pocket. RK-10 can be modified with reporter molecules of interest, such as biotin for IHC (RK-10-Biotin), or fluorescent molecules for fluorescentanalysis (RK-10-Cy5). As mentioned above, antibody based IHC agents recognizes different epitopes in PD-L1; in sharp contrast, the identified peptide sequence recognizes the unique binding site between PD-1 and PD-L1. Additionally, the peptide based assay developed in this study is standalone, that is secondary antibody is not necessary for staining.

RK-10 Peptide attached with biotin or fluorescent dye enables easy detection of PD-L1 biomarker in tissues and cell lines. The data presented in this study utilizes manual staining of RK-10 in human tissues; therefore, the need for autostainer specific for this agent is unnecessary. Additional advantages include that the peptide is relatively inexpensive, easy to synthesize, and can be mass produced in higher quantities.

The PD-L1 targeting peptide RK-10-Cy5 was identified through structural analysis of PD-1:PD-L1 binding pocket structure. PD-L1 specific peptide RK-10 has shown high sensitivity and specificity for tumor cells in over 200 different cases of tissue – 192 lung cancer cases on a TMA, seven patient lung cancers, one placenta tissue, and four Hodgkin’s lymphoma cases. Patient tissues stained specifically and reproducibly within the tumor and PD-L1 expressing immune cells using either a biotin-conjugated peptide for IHC, or a Cy5 fluorophore-labelled peptide for fluorescent microscopy. RK-10-Cy5 staining showed a positive correlation with Ventana’s FDA-approved PD-L1 diagnostic (SP263) where the SP263 kit stained tumor positively for PD-L1 expression. While there were some cases that were negative using both SP263 and RK-10-Cy5, there were a large number of cases where RK-10-Cy5 showed very specific tumor staining that were not stained by the SP263 antibody. This could either be due to higher sensitivity of RK-10-Cy5 or due to a lower titration of SP263 to only detect PD-L1 above a clinical cutoff, since the SP263 kit is meant for clinical diagnosis for use with its companion therapeutic drug Durvalumab.

In the Hodgkin’s lymphoma cases, PD-L1 expression as measured by the RK-10-Cy5 peptide matched up well with the IHC staining shown by the SP263 antibody, especially in the Reed-Sternberg cells. Since pembrolizumab was recently fast-tracked by the FDA to treat Hodgkin’s lymphoma cases, RK-10-Cy5 will need to be compared with the pembrolizumab companion diagnostic 22C3. Due to the multinucleate characteristics of the RS cells, it would be easy to detect and quantify the number of RS cells in a given Hodgkin’s tissue based on PD-L1 expression and nuclei. Since RK-10-Cy5 shows such specificity for tumor, it could detect a wide range of PD-L1 expression and inform more precise diagnostic levels for treatment. It has been shown that there is an urgent need for a PD-L1 diagnostic that can precisely detect PD-L1 protein irrespective of the drug intended to be used – a sensitive assay such as RK-10-Cy5 could be used to achieve this. Detection of PD-L1 expression in whole blood and metastatic melanoma suggests that RK-10-Cy5 could also potentially be used to detect low amounts of circulating tumor cells that express PD-L1.

Recent debates about the diagnosis of PD-L1 in patients highlight the need for refined methods of determining PD-L1 levels in the patient. By utilizing a peptide-based approach, we can detect all levels of PD-L1 with a high sensitivity and specificity. In a heterogeneous tumor, identification of PD-L1 expression using traditional methods may not be an accurate way of determining a binary IHC cutoff, but would rather require a wider range of diagnostic levels to determine optimal therapy. Recent studies have also shown tumors that express PD-L1 according to *in vivo* imaging methods, but upon excision for IHC no PD-L1 was detected^23^. Tumor mutations over a given period of treatment may lead to fluctuating PD-L1 levels, and as such may need to be monitored routinely.

## Electronic supplementary material

Supplementary Information
