# Supplementary material for: Identification and Validation of a PD-L1 Binding Peptide for Determination of PDL1 Expression in Tumors
Source: Sci Rep. 2017 Oct 20;7:13682. doi: 10.1038/s41598-017-10946-2 (PMC5651871; doi:10.1038/s41598-017-10946-2)
Supplement: Supplementary file 1 — Supplementary Information [file 41598_2017_10946_MOESM1_ESM.pdf]

Supporting Figures: Identification and Validation of a PD-L1 Binding Peptide for Determination of PDL1 Expression in Tumors

Charles Caldwell Jr, Cory E. Johnson, V. N. Balaji, Govardhan A. Balaji, Richard D. Hammer & Raghuraman Kannan

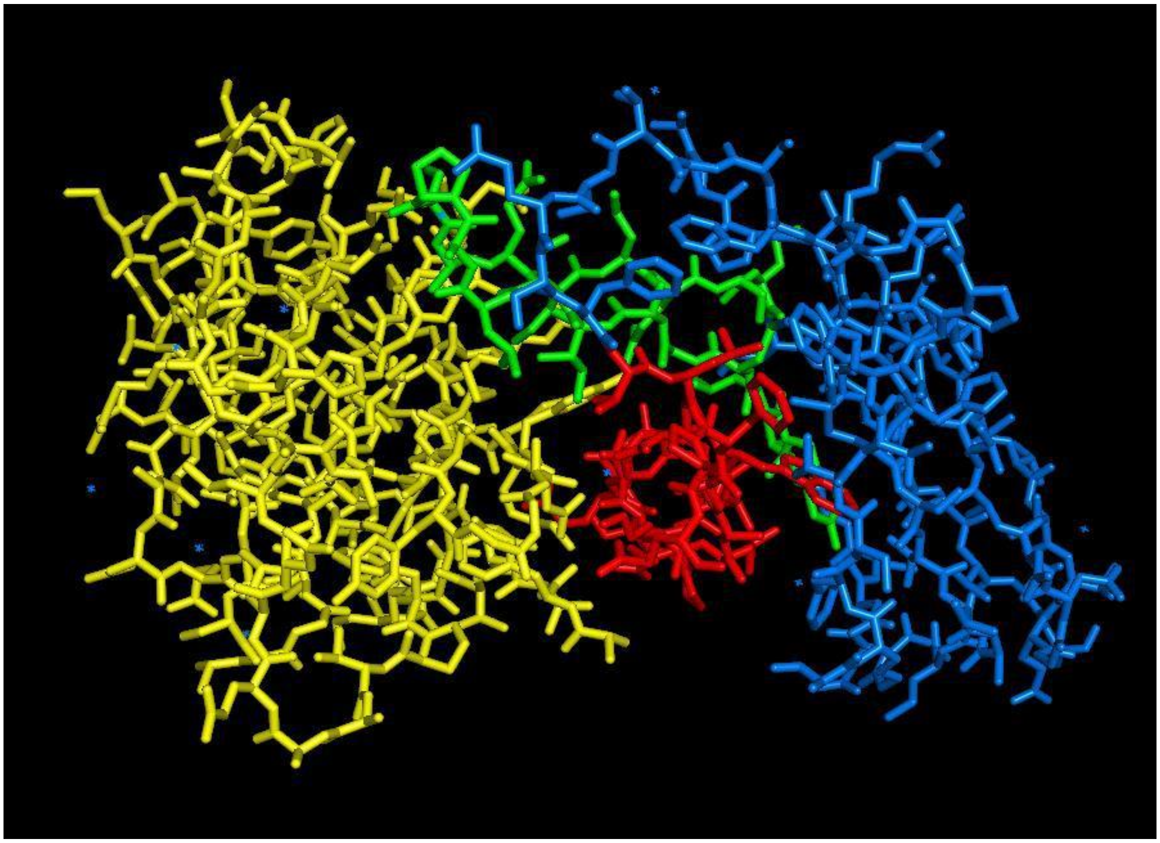

Figure S1. Identification of PD-L1 binding peptides. **Chain A** (PDL1) is shown in yellow. **Chain B** (PD1) is shown in blue, red and green. **Red and green** corresponds to the sequence 1 and 2 suggested for synthesis which are in contact with PDL1. These two sequences were selected based on the contact criteria and extension beyond the contact residues to include extended chains to retain its secondary structure.

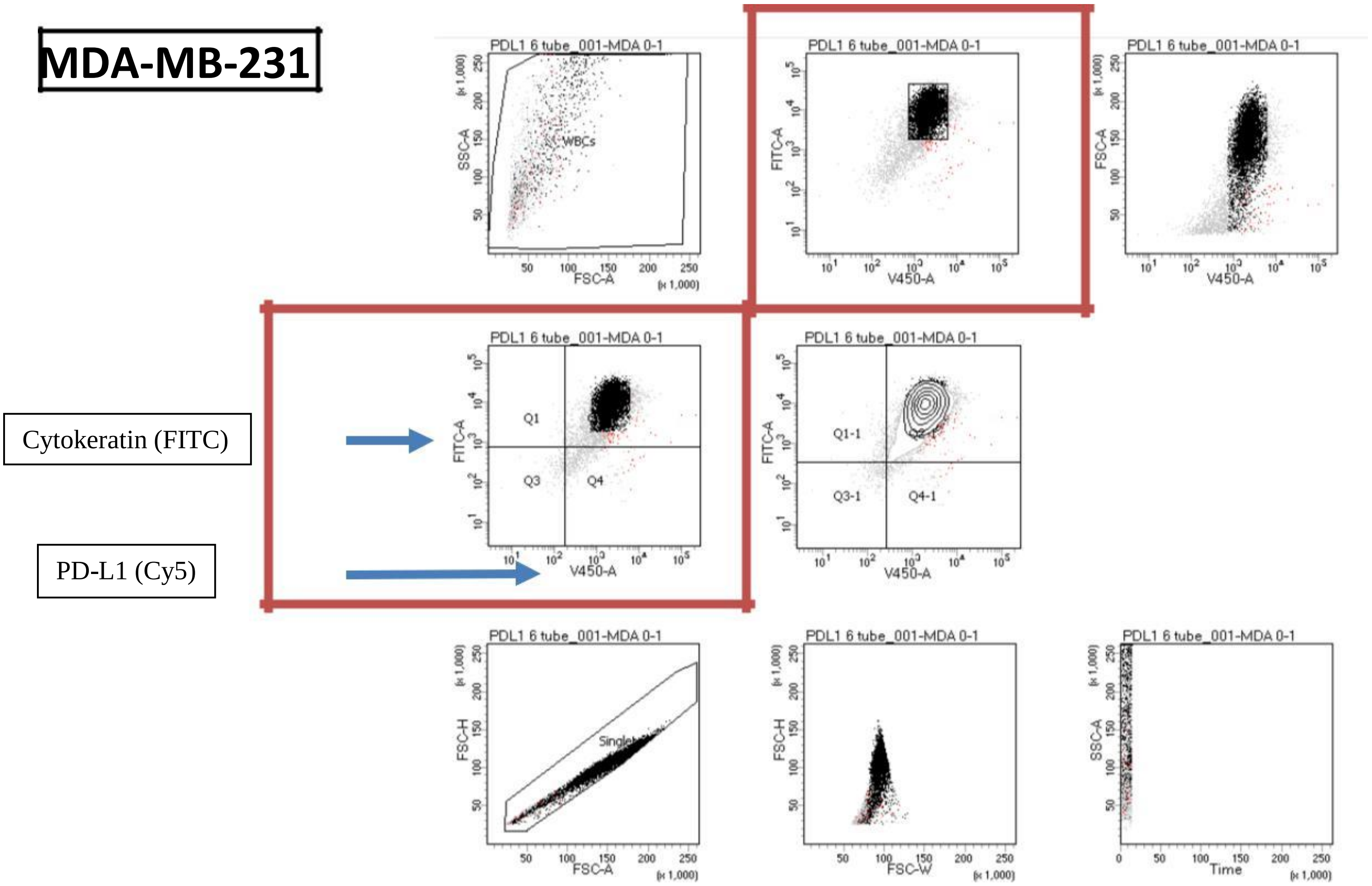

Figure S2. Flow Cytometry Method. Treated cell lines were double-gated for both cytokeratin (FITC) expression and PD-L1 expression (Cy5)

0.1 mg/ml

0.05 mg/ml

0.01 mg/ml

0.005 mg/ml

0.001 mg/ml

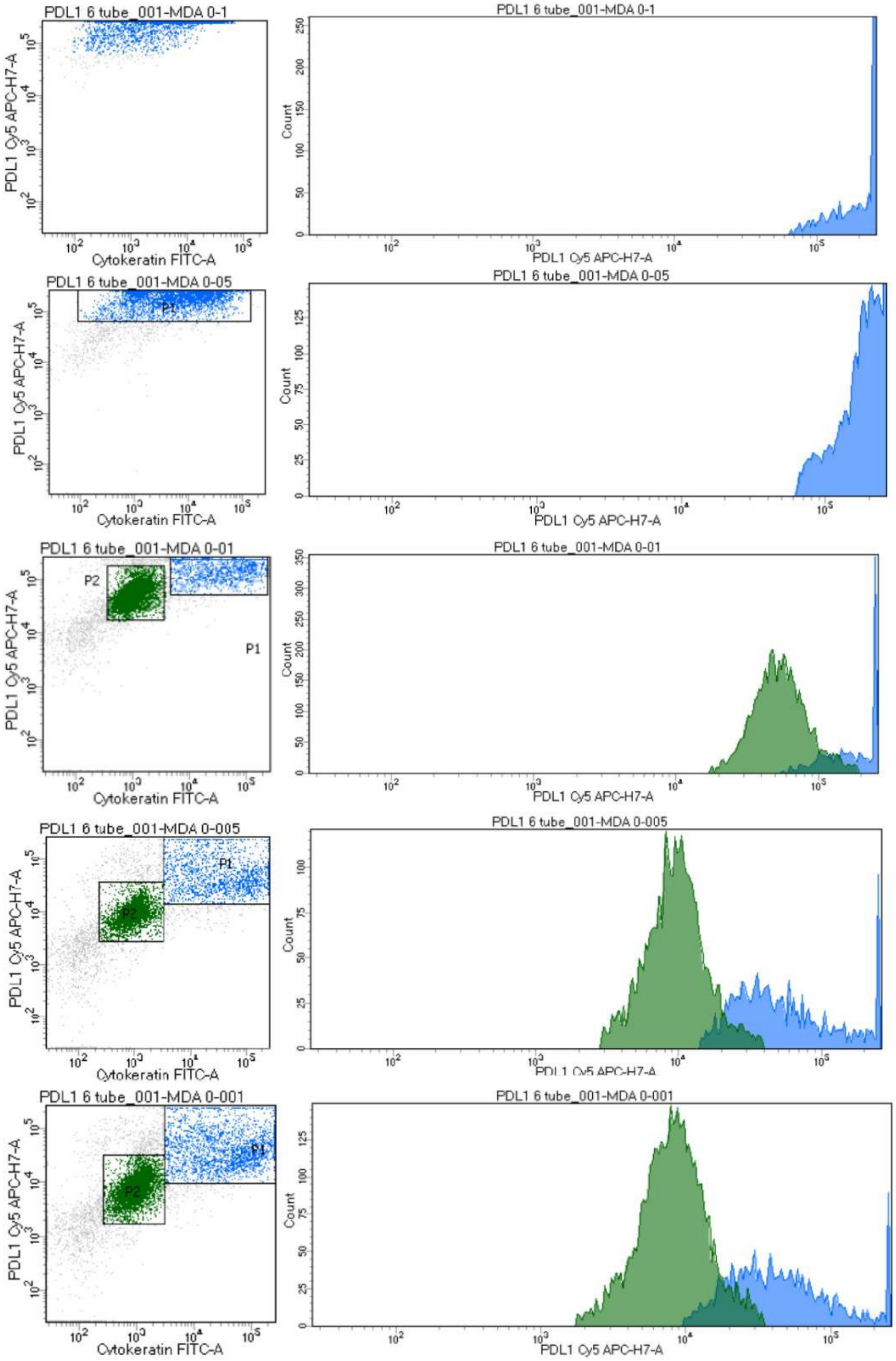

Figure S3. PD-L1 expressing MDA-MB-231 cell lines treated with titrations of PD-L1 peptide at the indicated concentrations

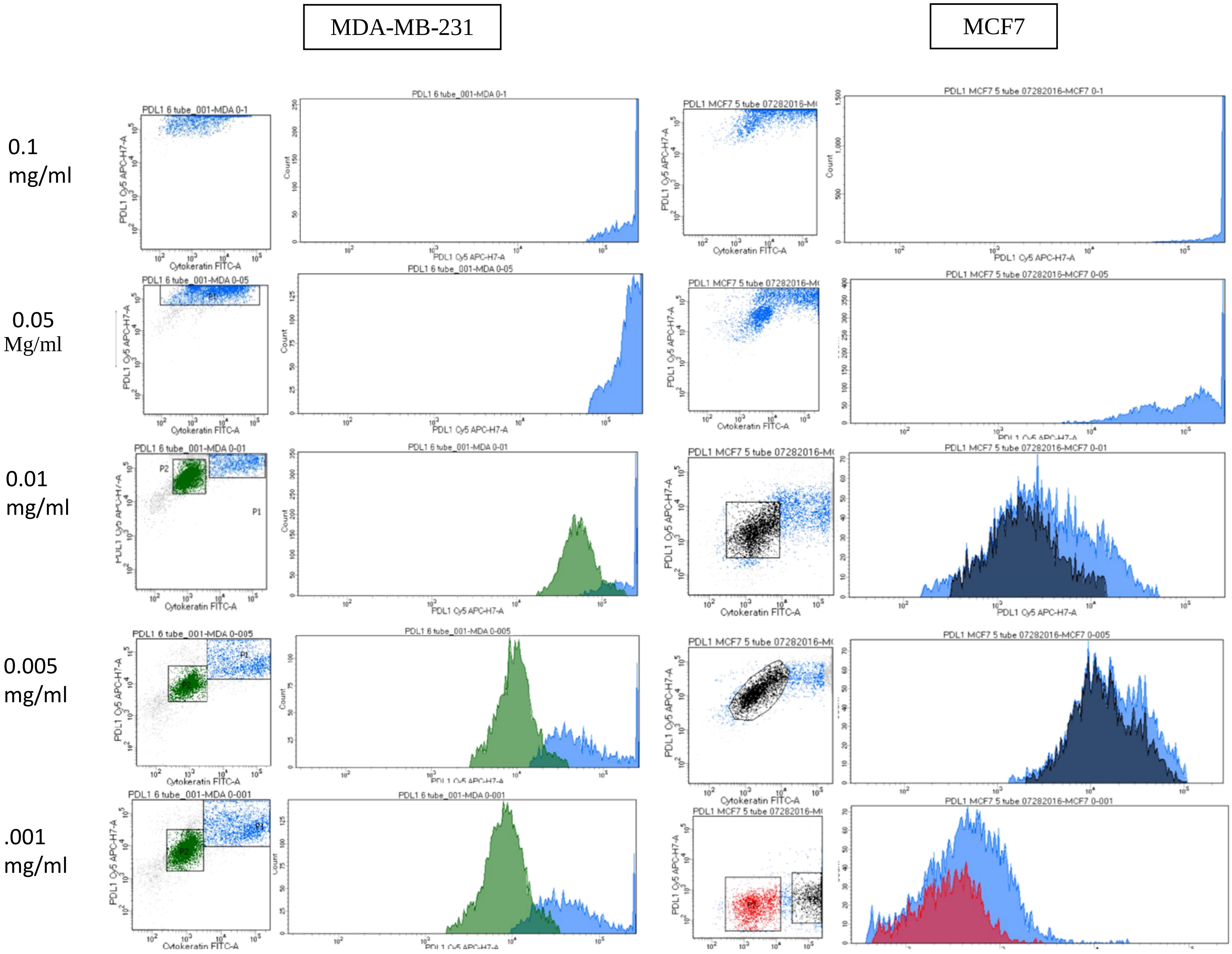

Figure S4. Titrations of PD-L1 peptide compared in PD-L1 high MDA-MB-231 and PD-L1 low MCF7. MCF7 treated at .005mg/ml had been treated twice with peptide.

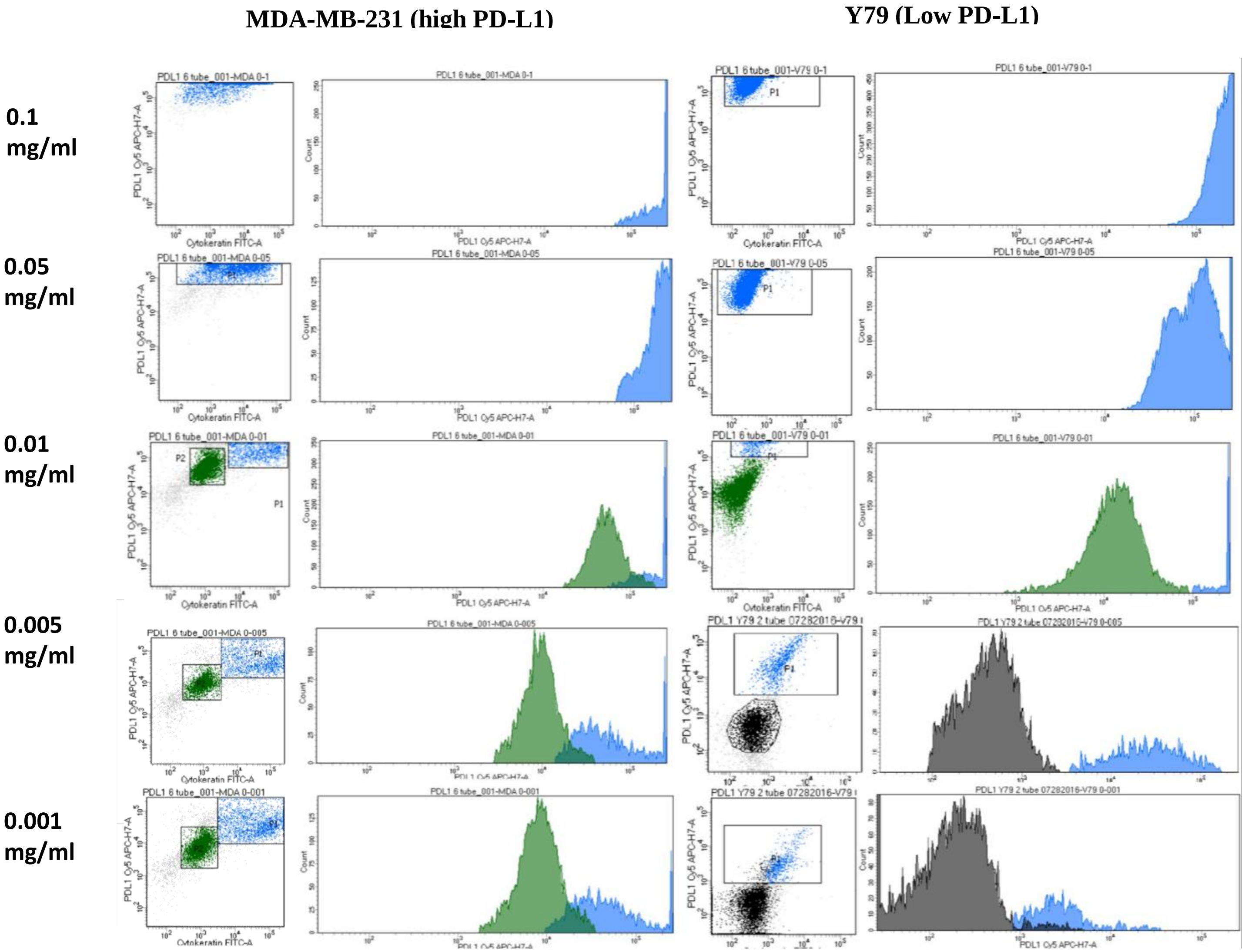

Figure S5. Titrations of PD-L1 peptide compared in PD-L1 high MDA-MB-231 and PD-L1 low Y79

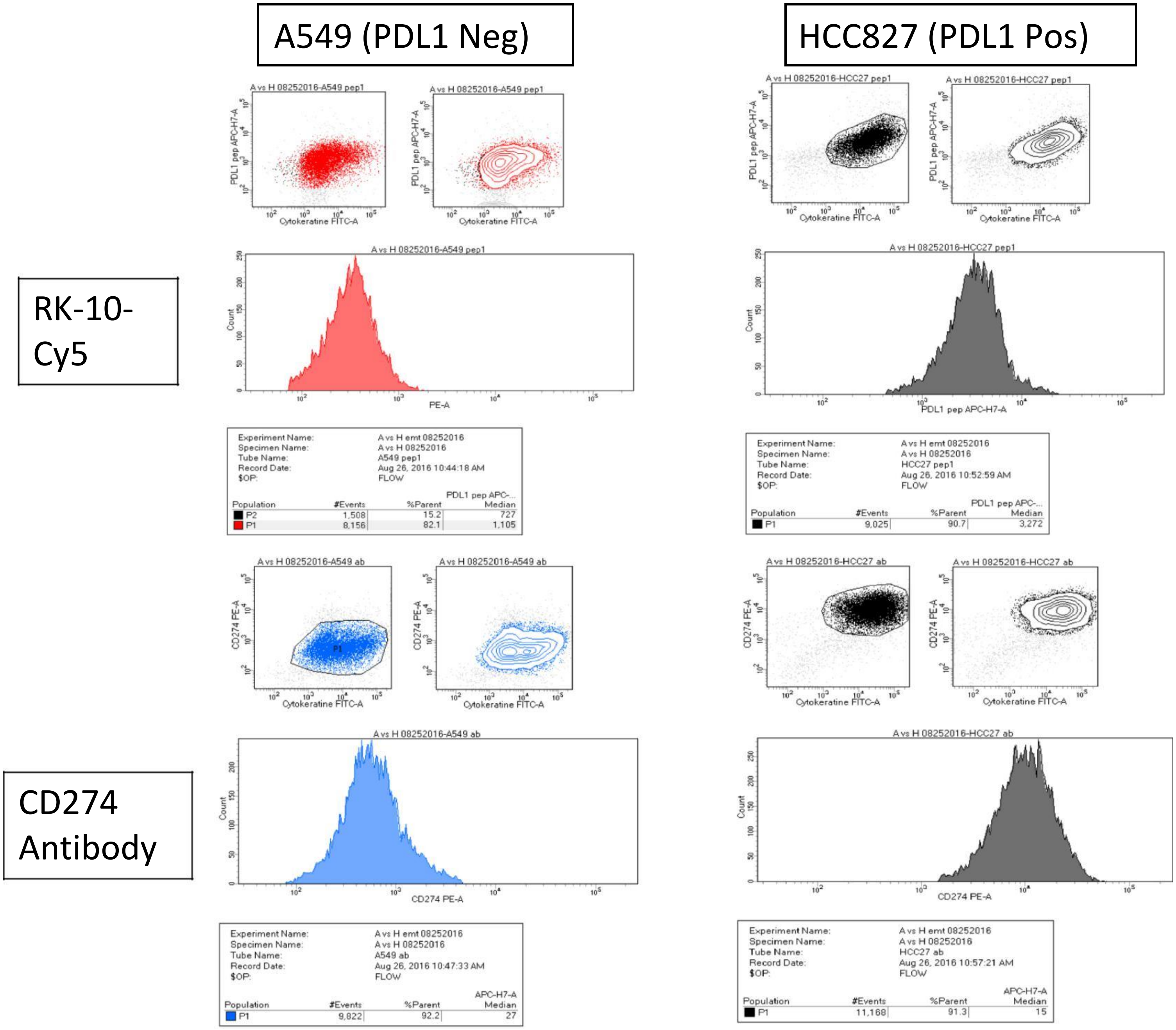

Figure S6. A549 and HCC827 cell lines were treated with RK-10-Cy5 peptide or CD274-PE antibody and analyzed for PD-L1 expression using Flow Cytometry

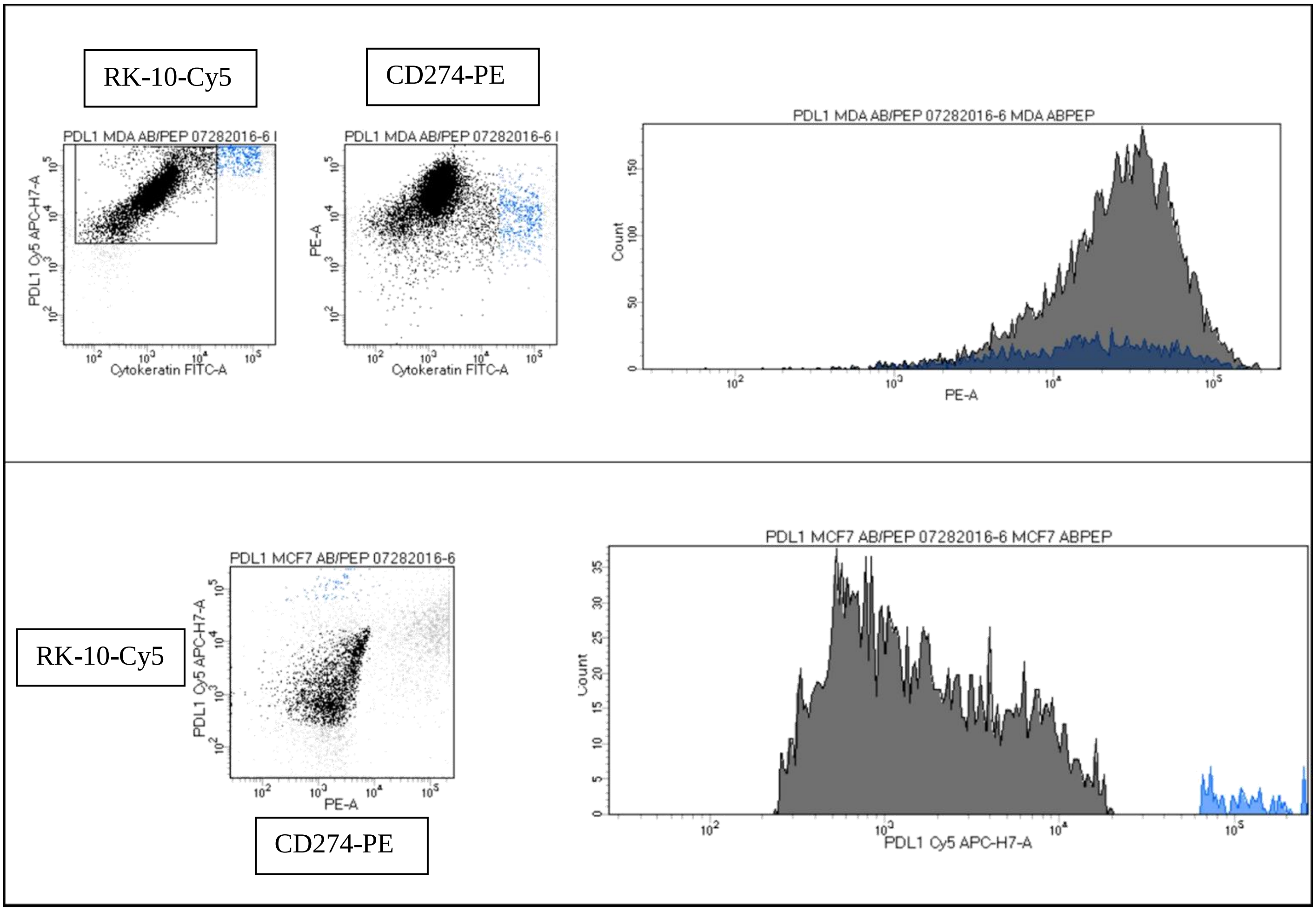

Figure S7. MDA-MB-231 cell line treated Simultaneously with RK-10-Cy5 and CD-274-PE antibody shows similar expression using both methods

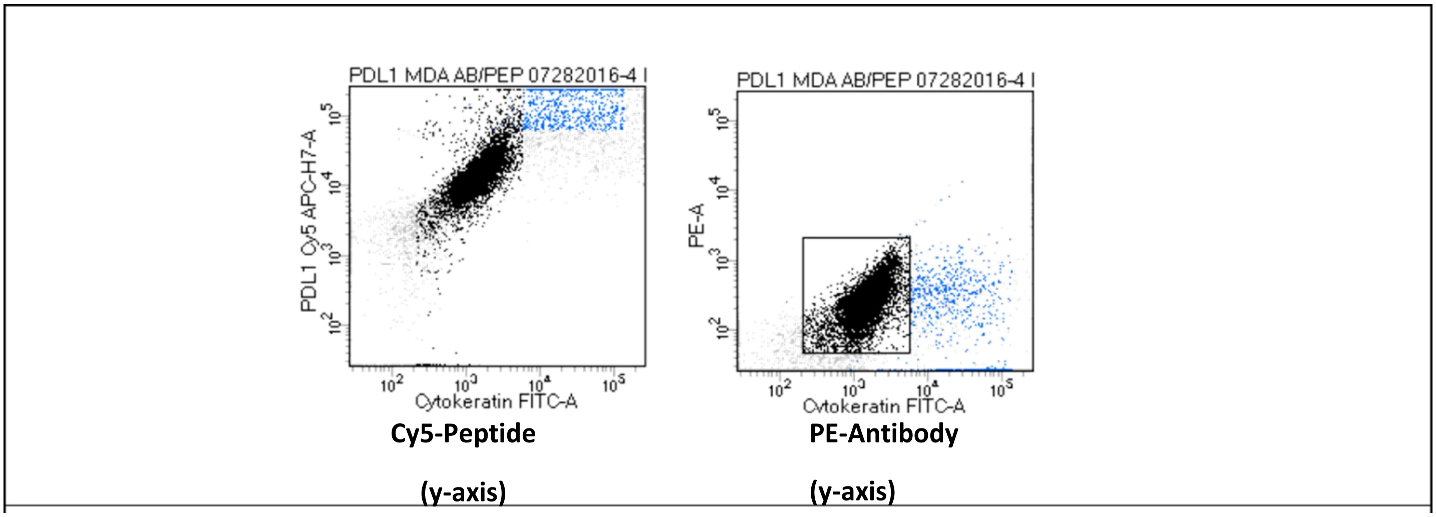

Figure S8. MDA-MB-231 cell line treated with RK-10-Cy5 for 1 hour, then treated with CD274-PE for 30 minutes shows decrease in CD274-PE expression.

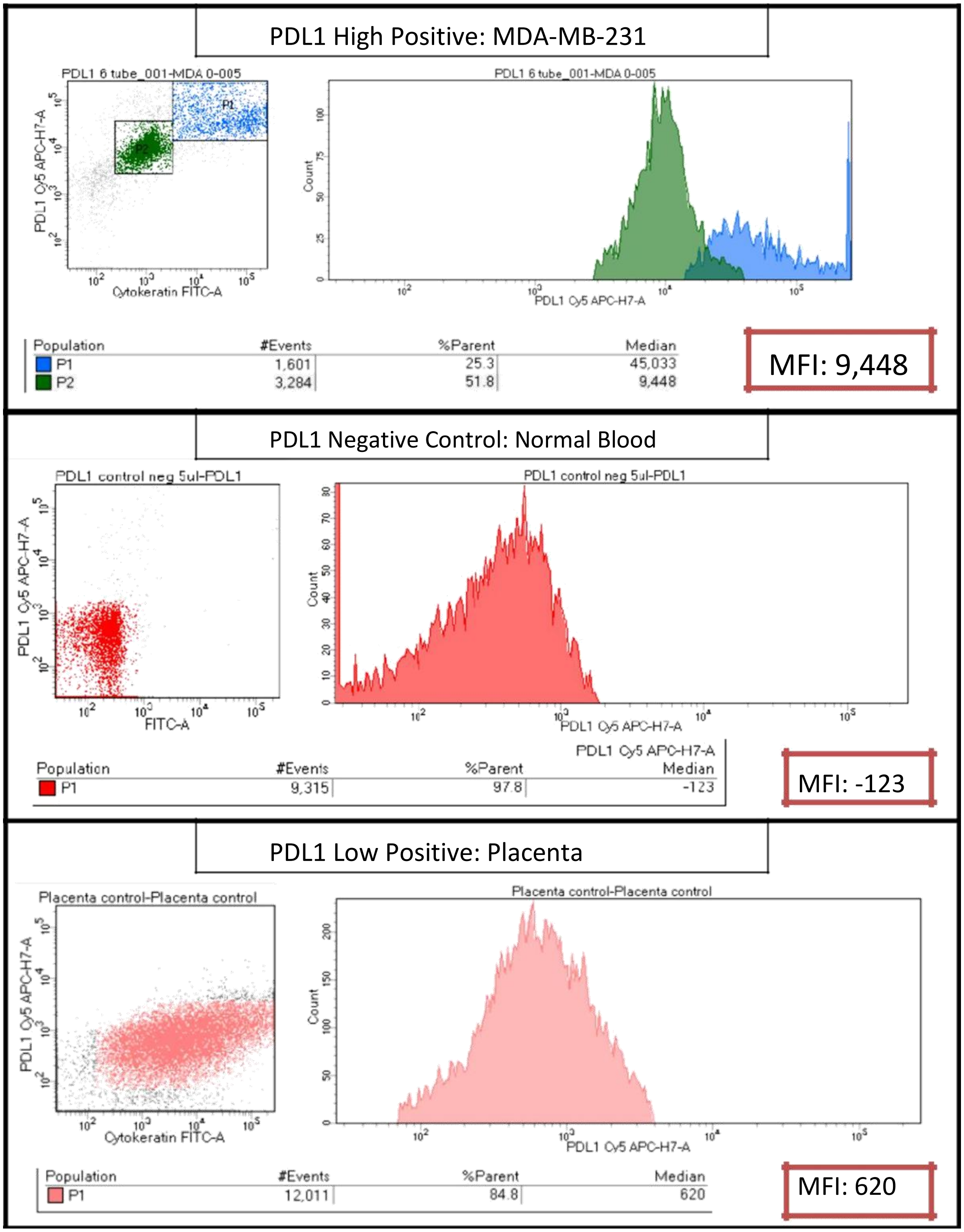

Figure S9. Established controls for PD-L1 expression using flow cytometry shows high and low Mean Fluorescent Intensity of PD-L1 expression

Patient Tissue: Squamous Cell Carcinoma, 50 µL Peptide (0.05mg/mL)

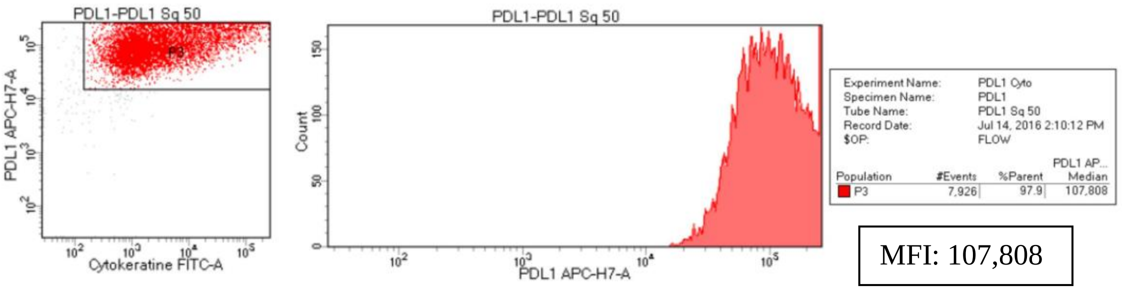

Patient Tissue: Squamous Cell Carcinoma, 100 µL Peptide (0.05mg/mL)

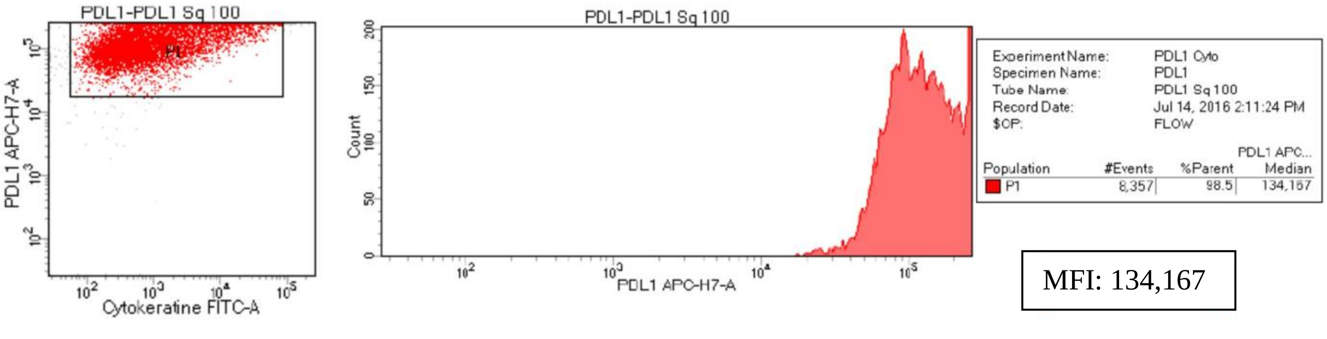

Figure S10. Squamous cell carcinoma patient tissue treated with increasing amounts of RK-10-Cy5 peptide shows high PD-L1 expression, with slight increase of fluorescent signal as amount of peptide added increases

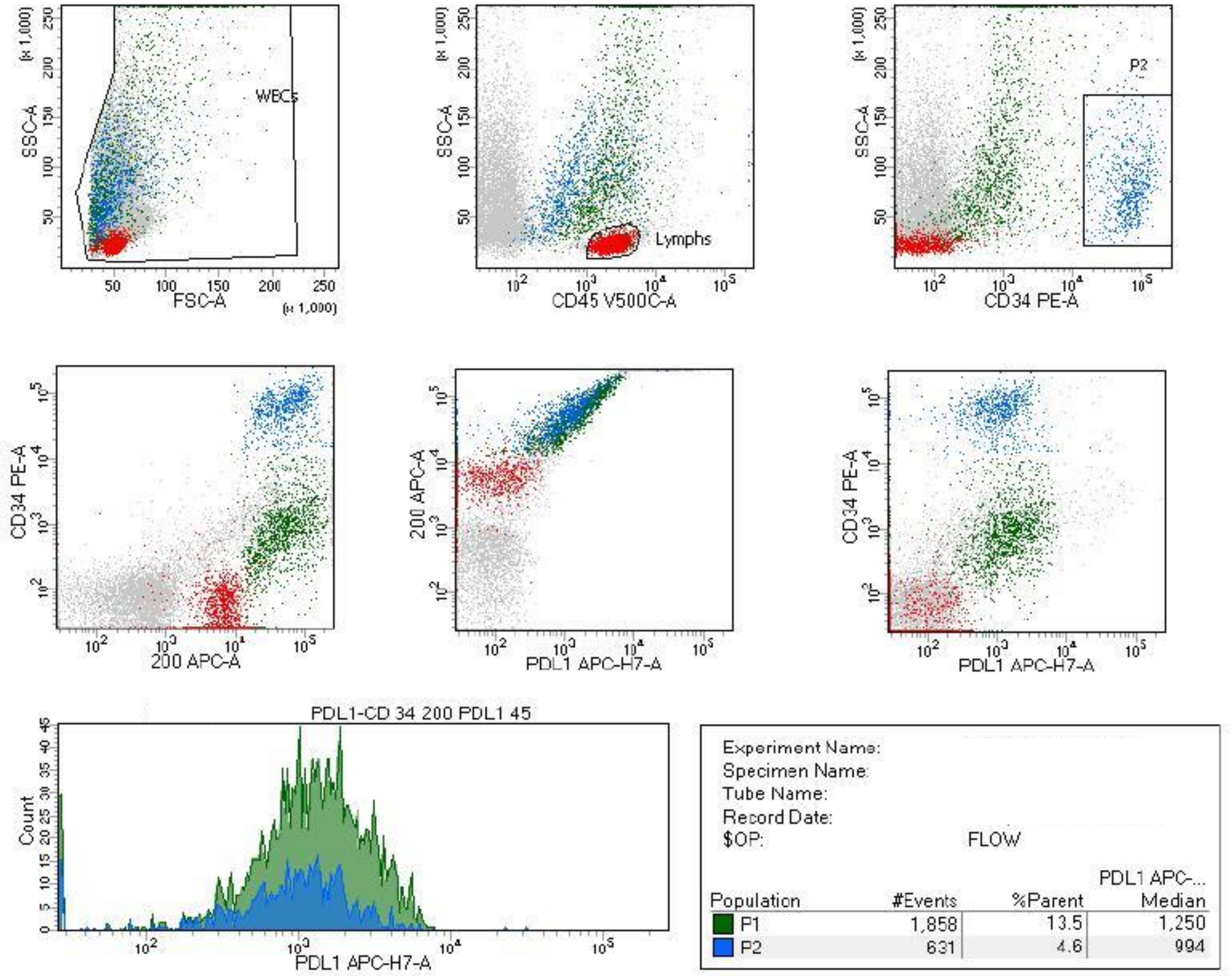

Figure S11 Patient melanoma was identified with the unique phenotype of bright CD34, HLA-DR, moderated CD56 and negative CD45. **There was moderate PDL-1 using RK-10-Cy5** and no expression of cytokeratin by flow cytometry. The tissue biopsy showed metastatic melanoma, which was positive for A103 and Sox 10 and negative for cytokeratin. The morphology, flow and phenotype were diagnostic of melanoma

10x Magnification 20x Magnification

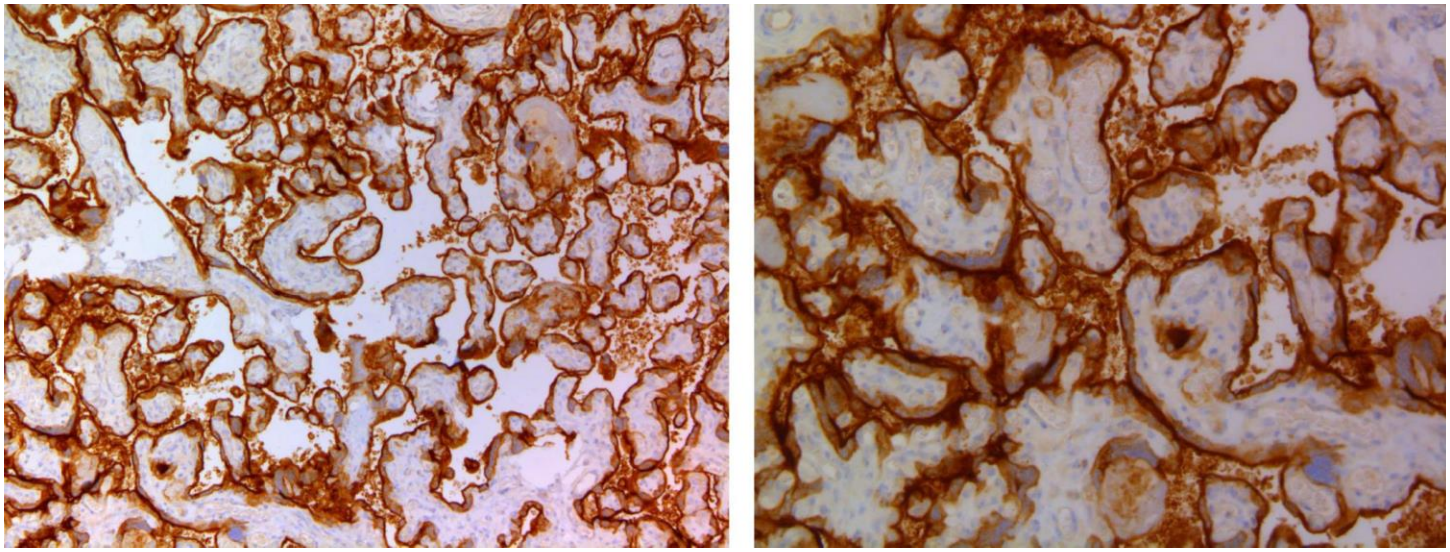

Figure S12 Placenta tissue obtained from Mizzou tissue bank is used as our positive control tissue since trophoblast cells will express PDL1

15µM Concentration

10x Magnification 20x Magnification

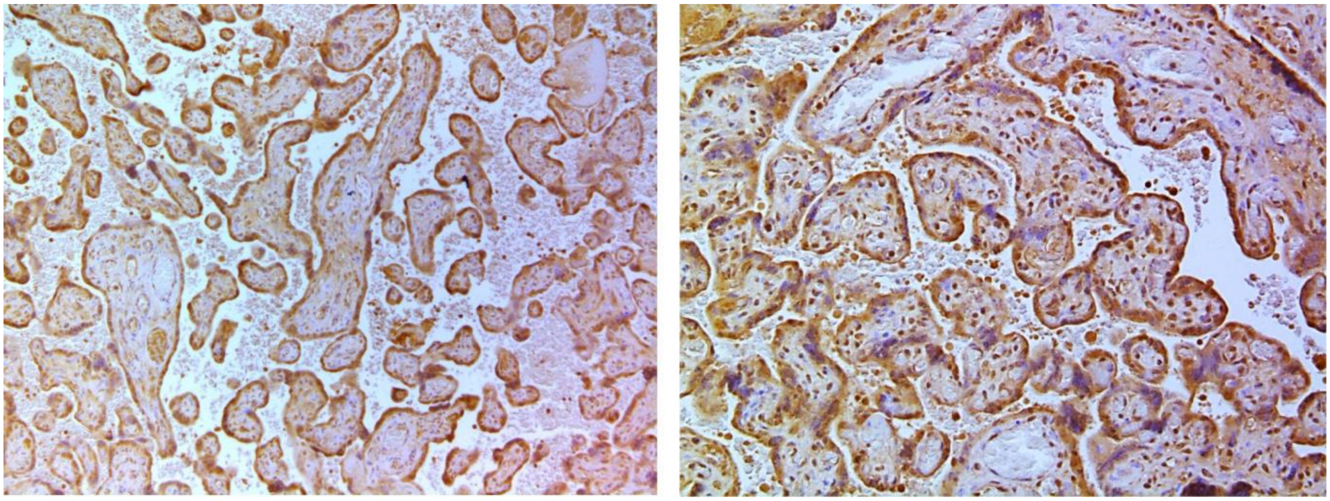

Figure S13. Placenta tissue treated with RK-10-Biotin shows specific staining of trophoblast cells.

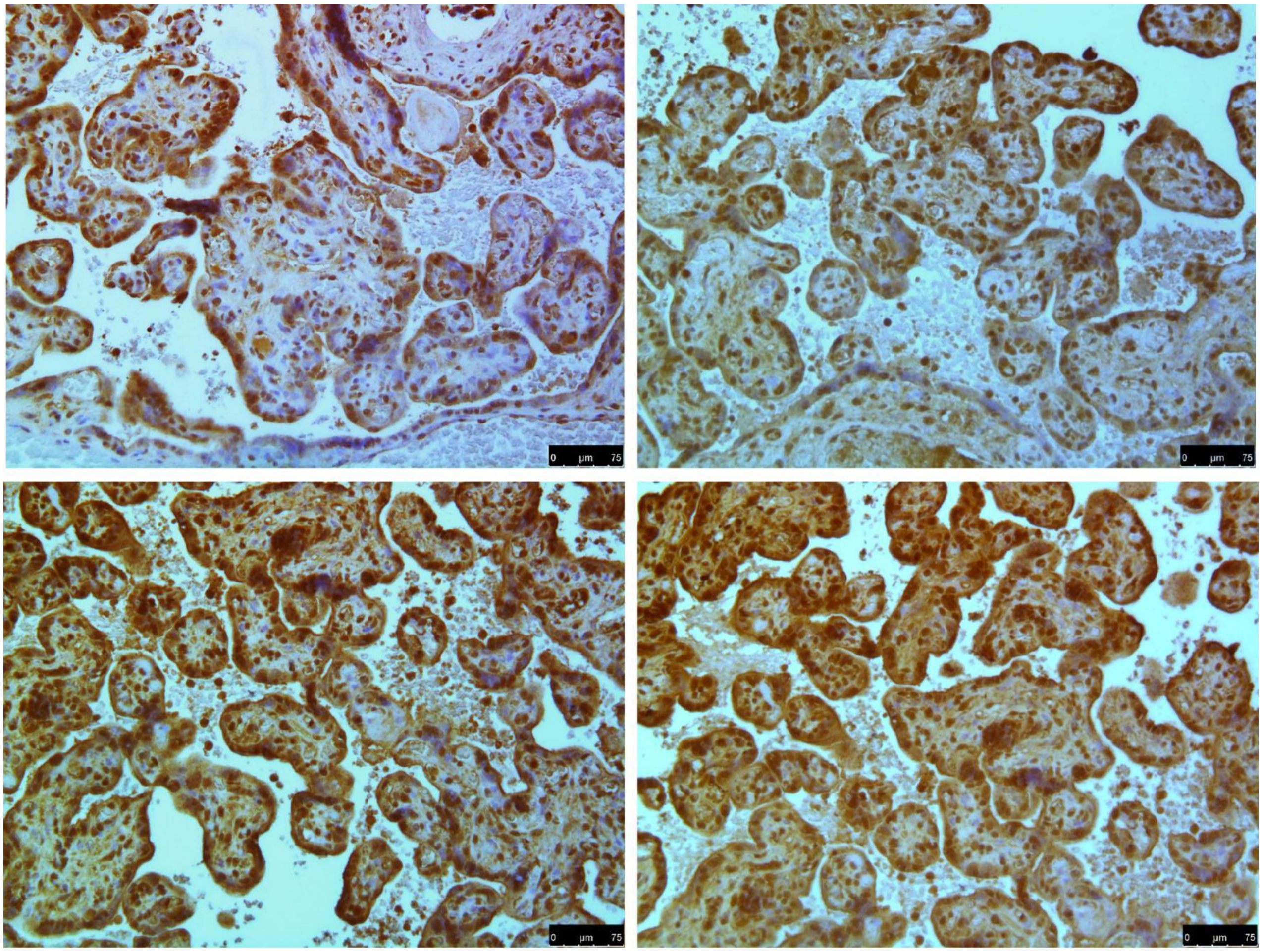

Figure S14. Placenta tissue stained with RK-10-Biotin is amplified using high-sensitivity Streptavidin-HRP

Mock Peptide RK-10-Biotin

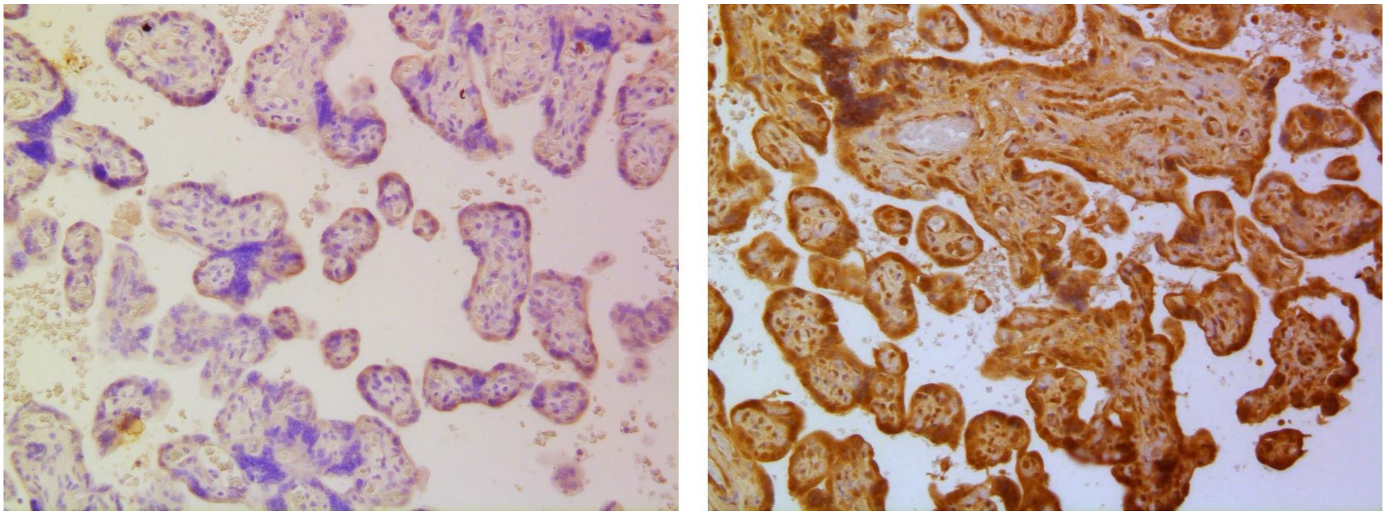

100µM Concentration 50µM Concentration

Figure S15. Mock peptide RK-11-Biotin was designed to have lower affinity than RK-10-biotin. Placenta tissues stained with both peptides are shown. Mock peptide RK-11 showed highly decreased specificity for placental trophoblasts compared to RK-10 peptide.

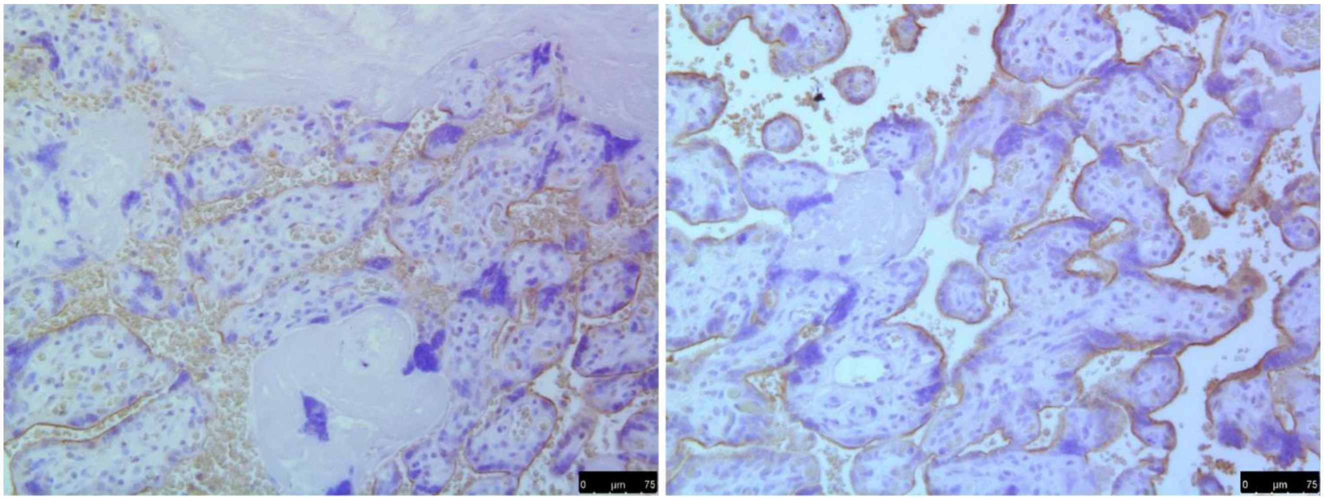

Figure S16. Placenta tissue was first blocked with 15μM peptide for 2h, washed, then put on the SP263 autostainer with the SP263 PDL-1 antibody IHC kit

Patient A

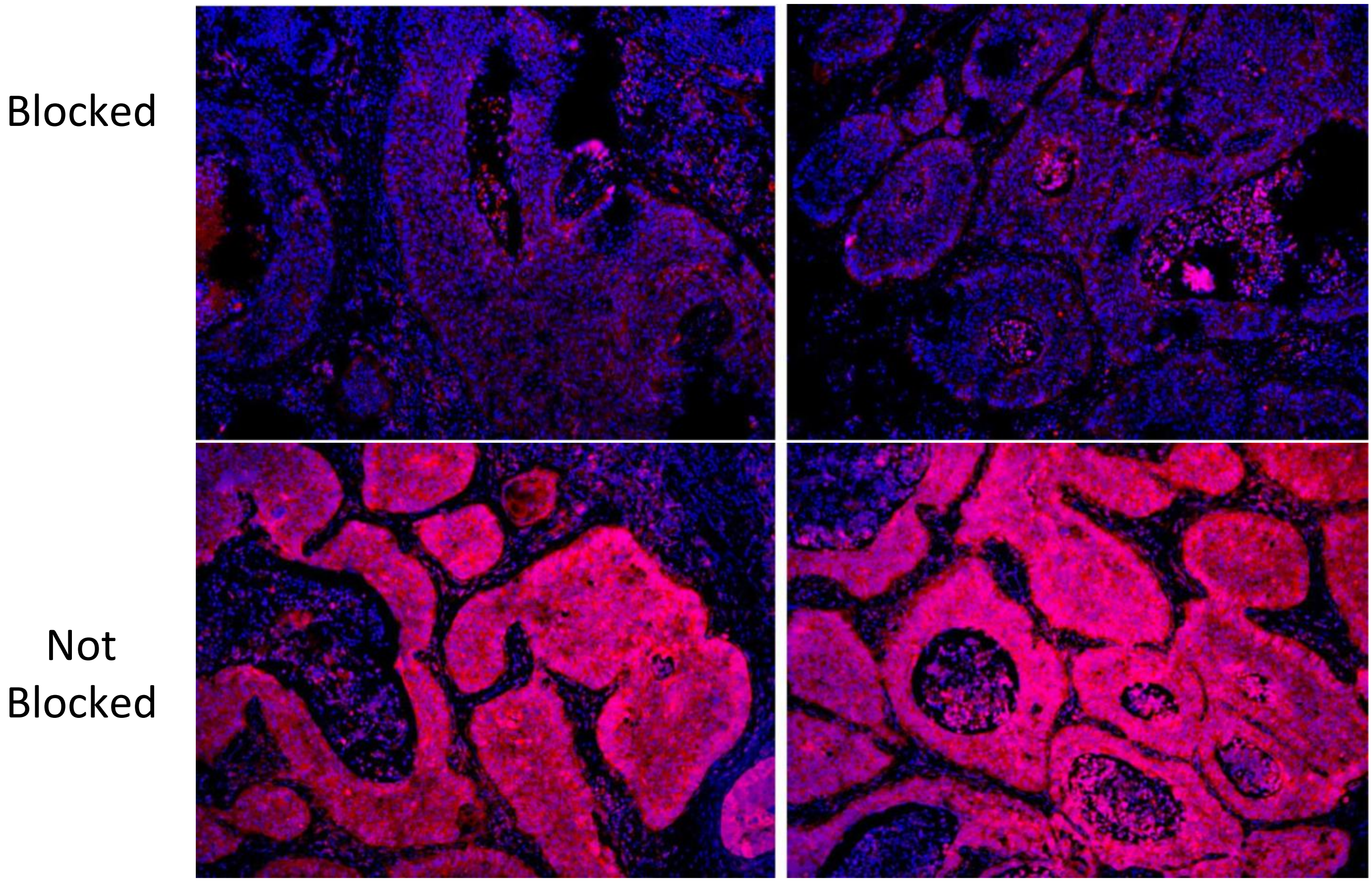

Figure S17. Images of patient tumor tissues Blocked and Not Blocked with RK-10 peptide are taken under the same illumination conditions. No adjustments were made – leading to an overexposed Cy5 channel in the 'not blocked' sample.

Patient A

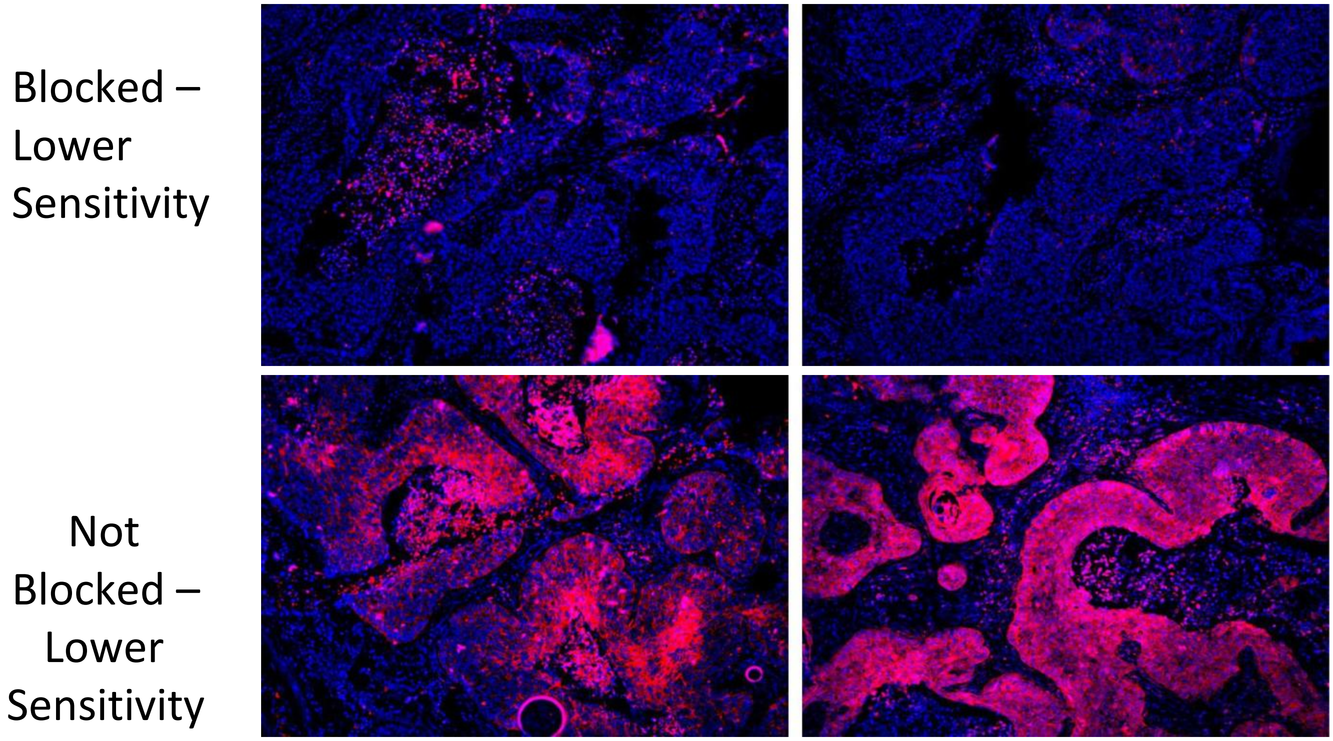

Figure S18. Cy5 Sensitivity was lowered until No Cy5 was seen in the Blocked Samples. The Unblocked samples still gave a strong Cy5 signal in the tumor. No illumination changes between the two samples were made.

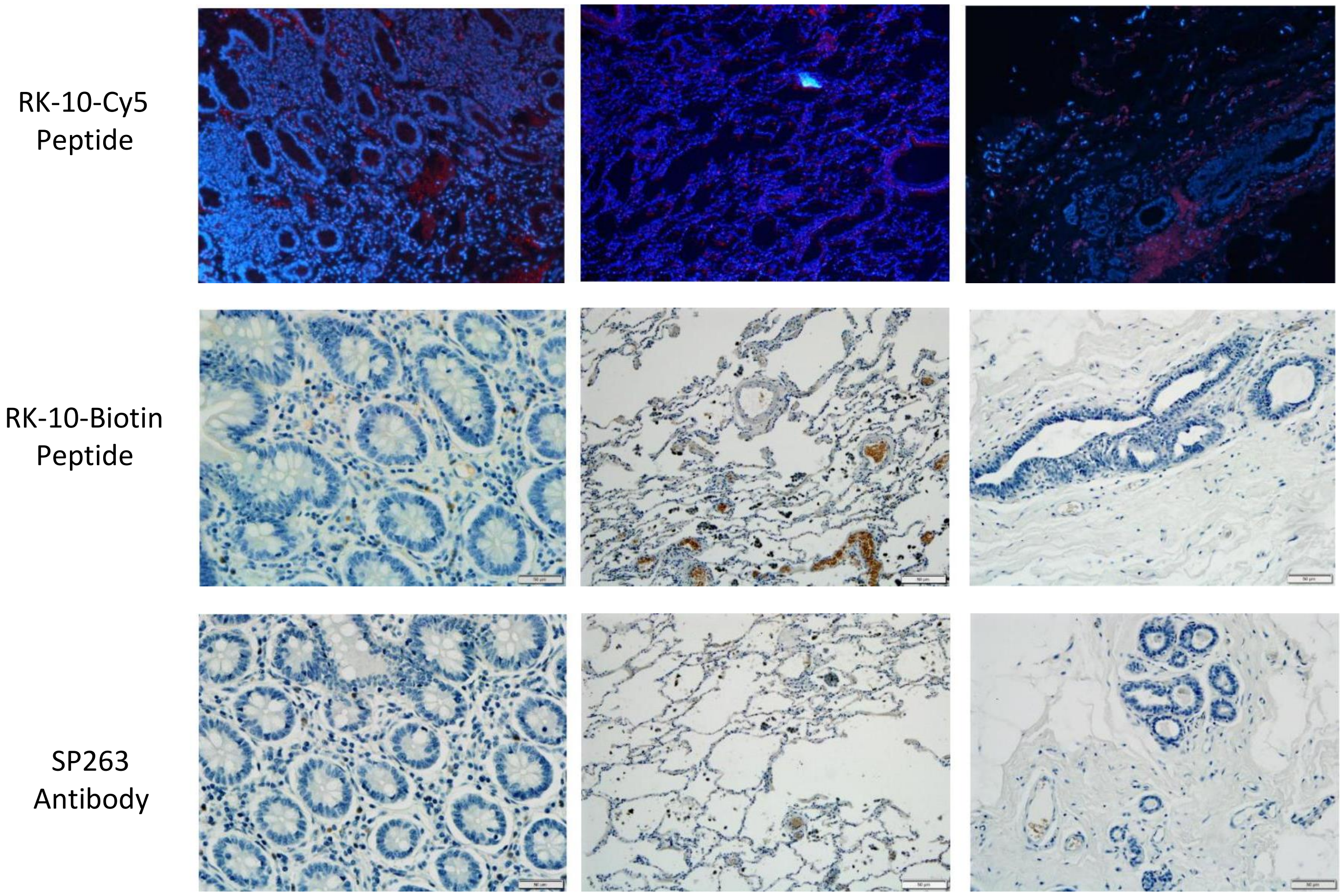

Figure S19. Normal Colon (left), Lung (center), and Breast tissues stained for PD-L1 expression using SP263 antibody or RK-10 peptide constructs

SP263

Cy5 Peptide

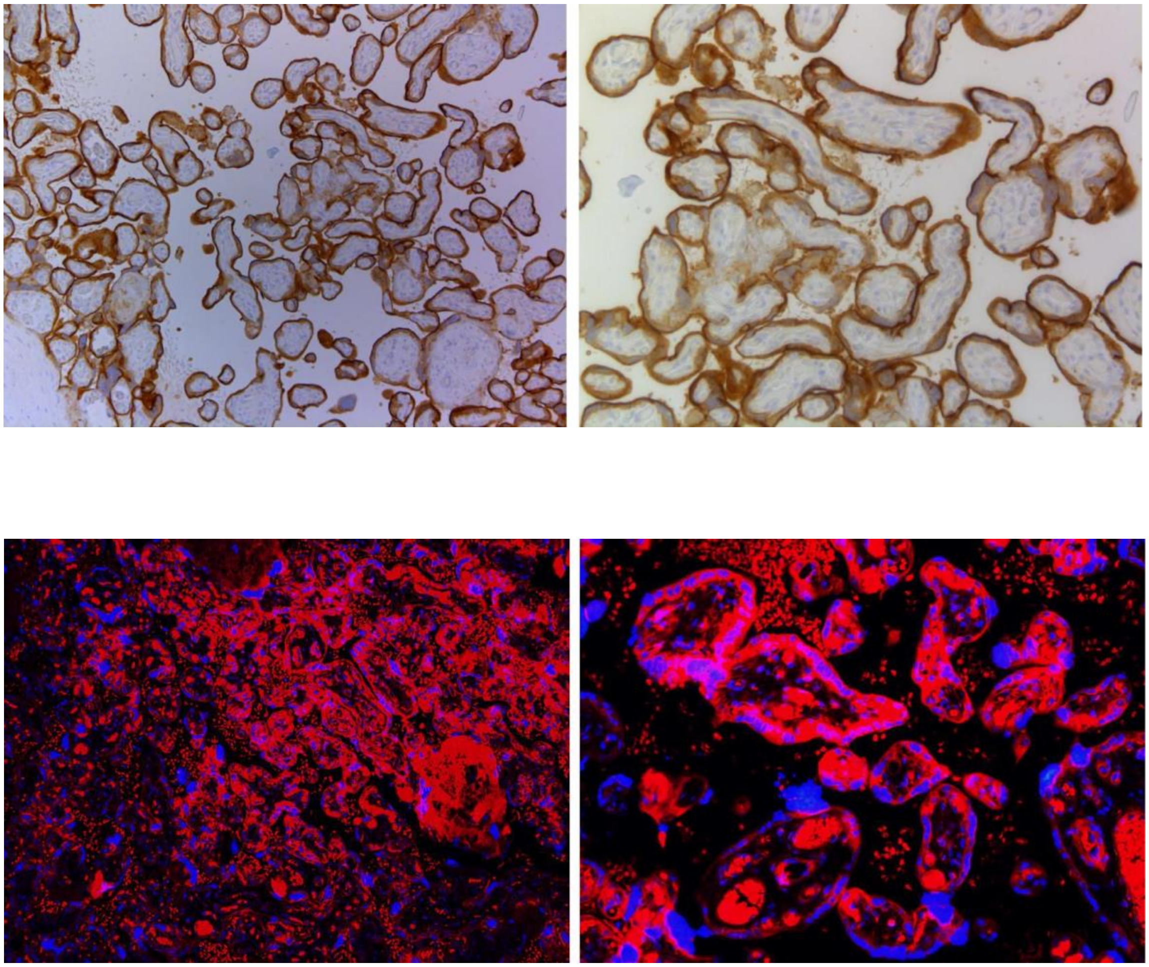

S20. High sensitivity observed with the Fluorescent Peptide staining in Placental control tissue using RK-10-Cy5

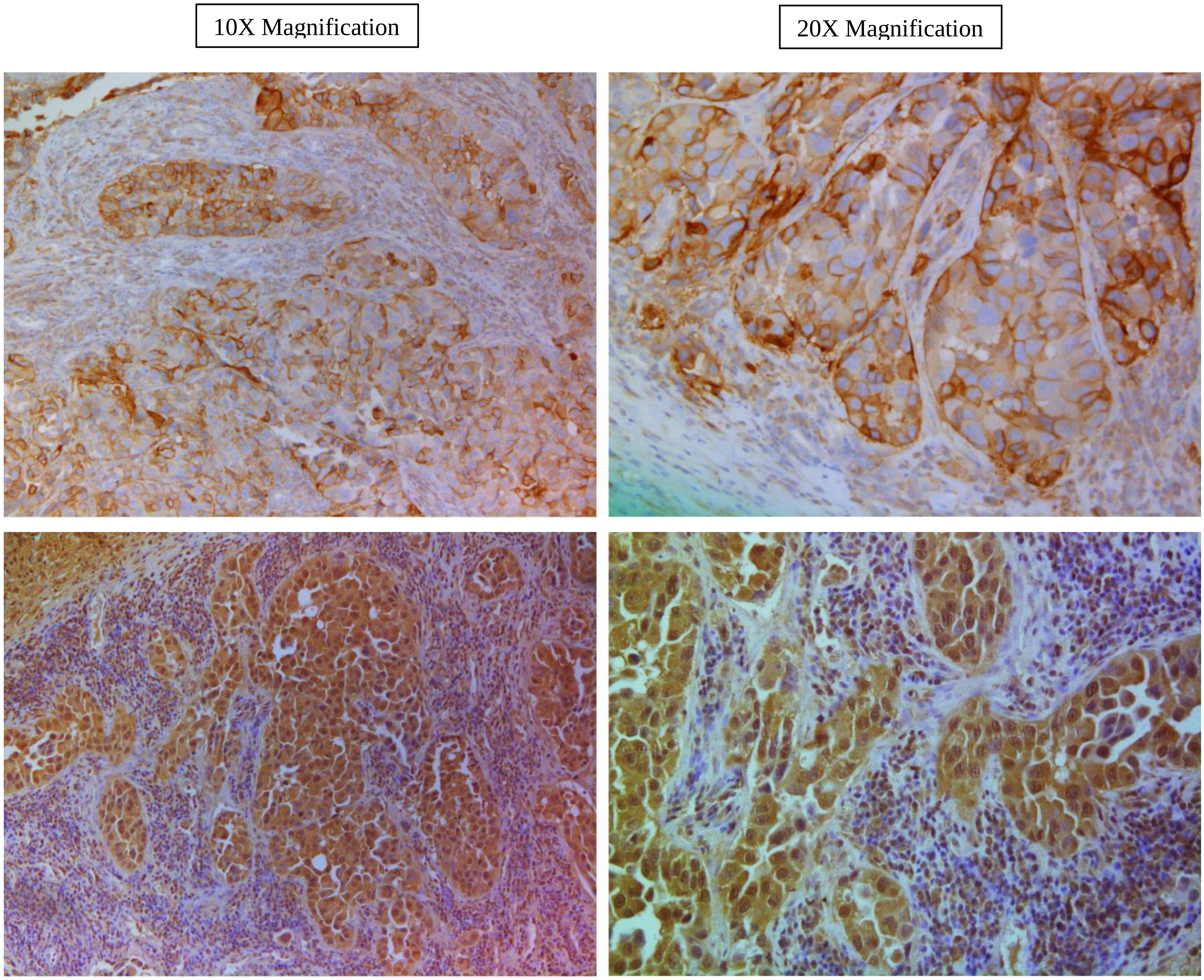

Figure S21. Patient Tissue from Mizzou clinical pathology lab was identified as tumor positive for PDL1 by both SP263 antibody kit on autostainer and with peptide using manual IHC

SP263 Kit,
Autostained.
Tumor (marked)
Stains Very
Faintly

Peptide,
Manual IHC.
Heavy Tumor
Staining.

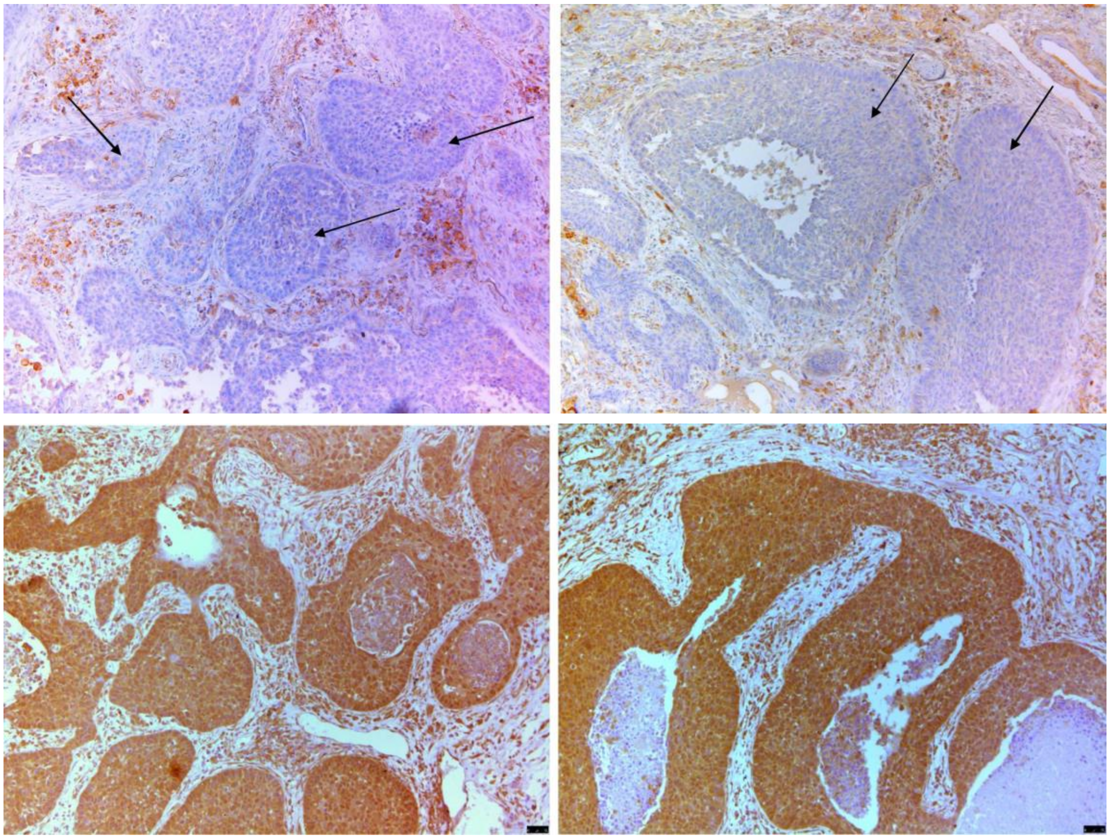

Figure S22. Patient A Stained with both SP263 and RK-10-biotin shows heavy staining of the tumor cells by RK-10 peptide but not SP263.

Tissue cut immediately prior to staining with peptide

Tissue Cut 3 Months Prior to Staining (April-July)

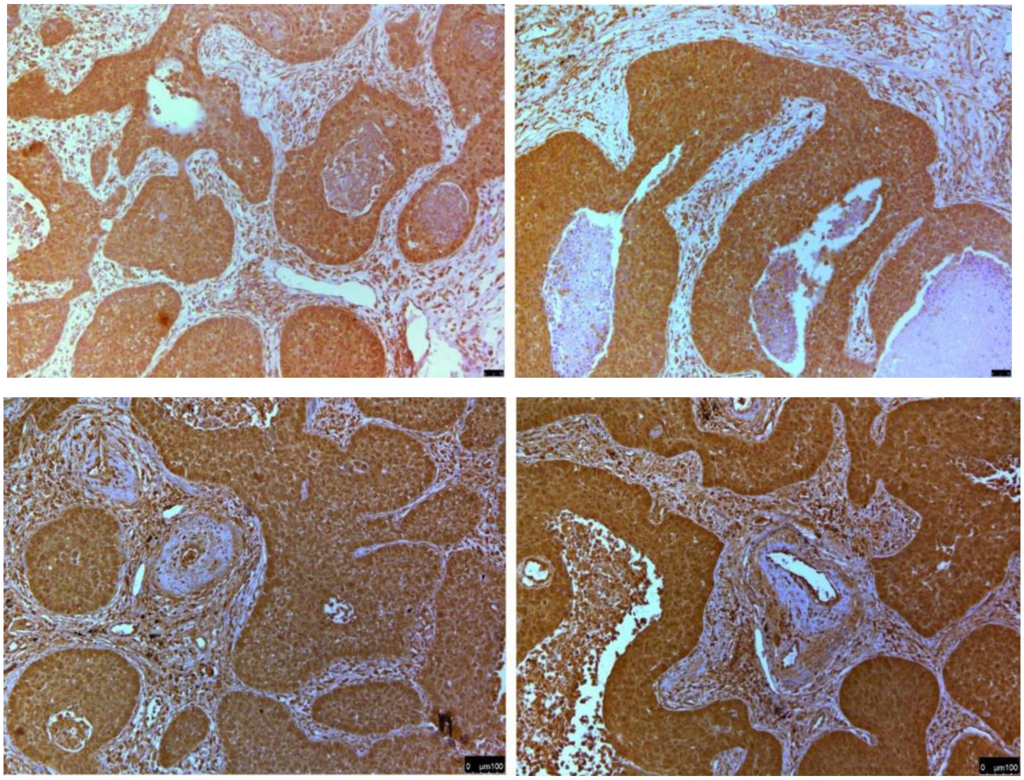

Figure S23: Patient A IHC with RK-10-Biotin: Tissue Cut 3 Months Prior To Staining

SP263 Kit, Autostained. Faint/No Tumor staining

Peptide, Manual IHC. Good Tumor staining.

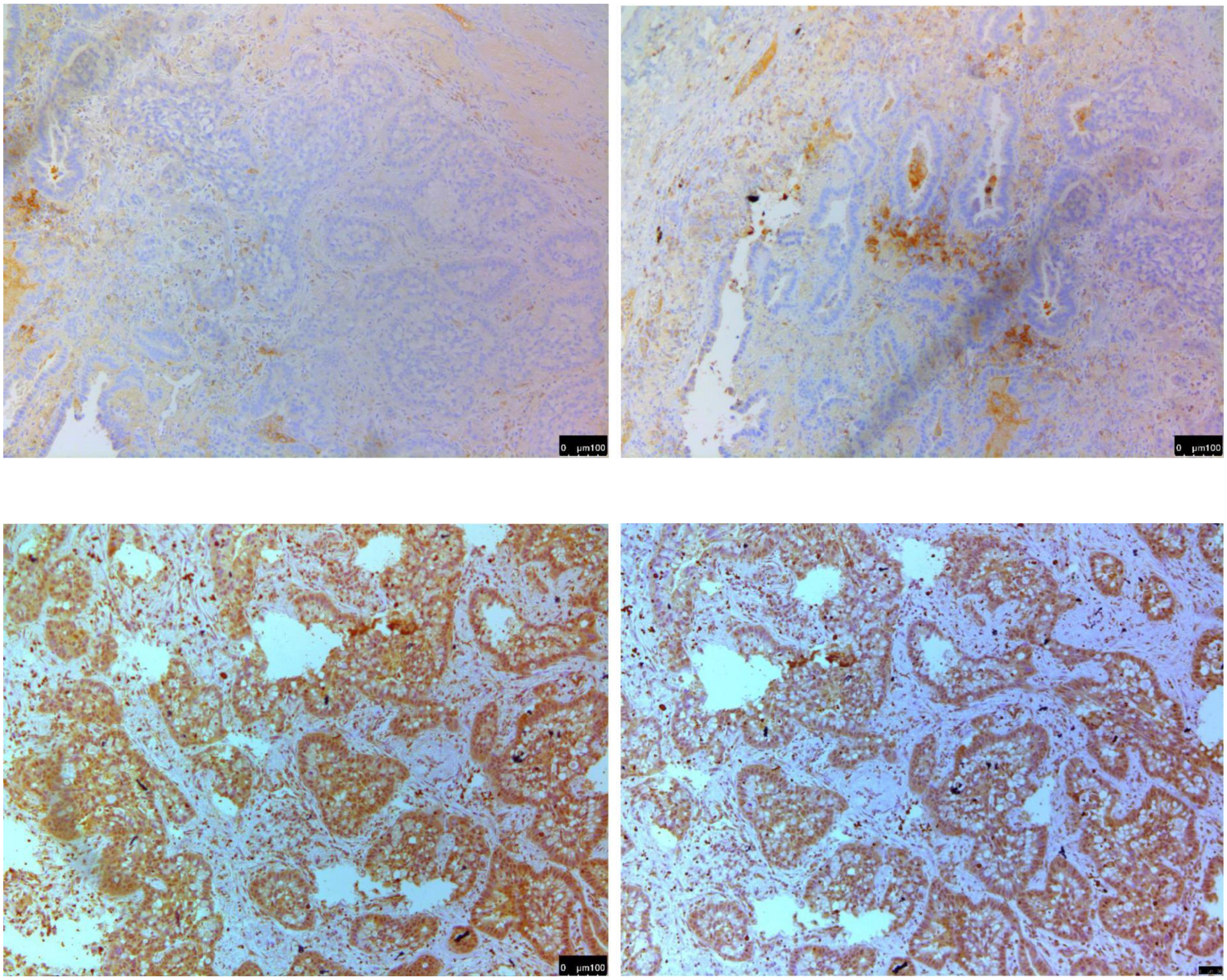

Figure S24: Patient B stained with SP263 and RK-10-Biotin shows specific staining in the tumor region using RK-10.

SP263 Kit, Autostained. Some tumor areas stain

Peptide, Manual IHC. Consistent Tumor staining.

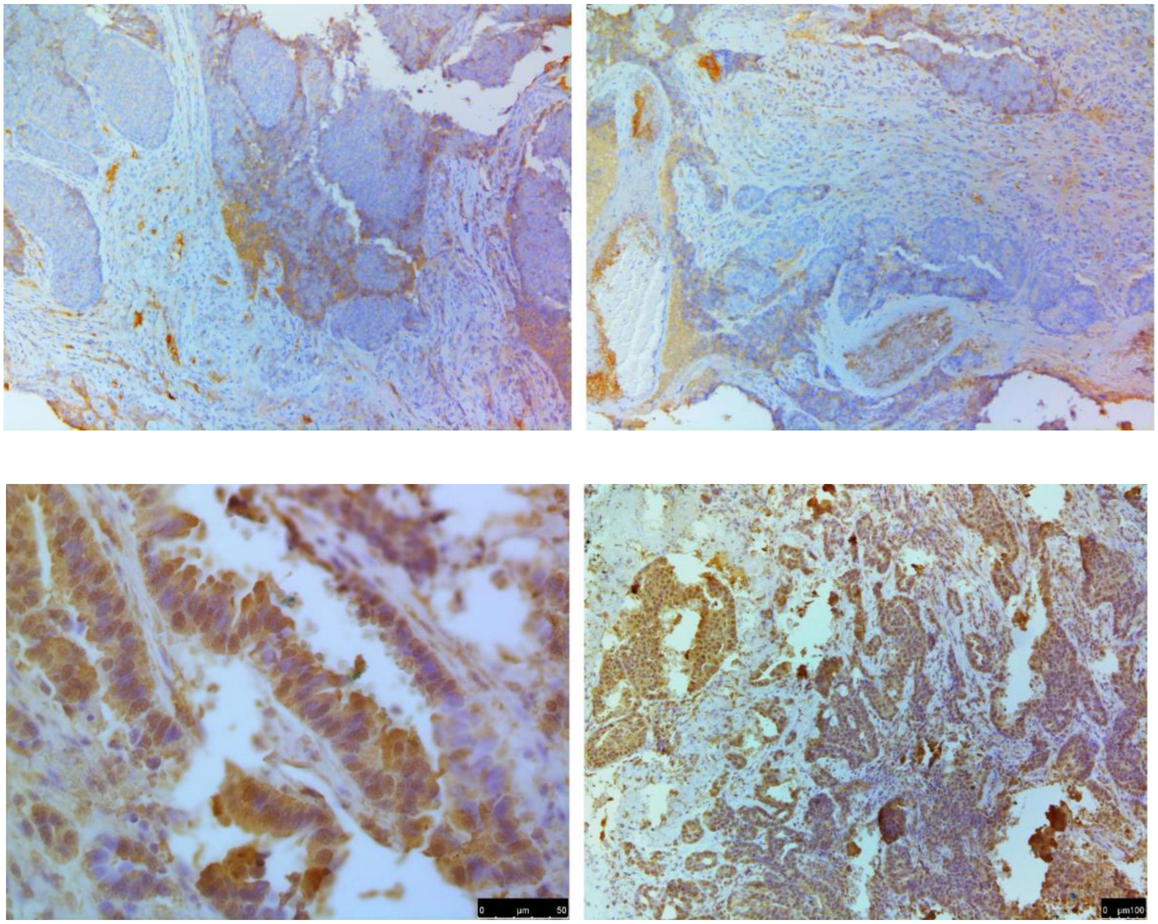

Figure S25: Patient C stained with SP263 and RK-10-biotin

SP263 Kit, Autostained. Faint/spotty tumor staining

Peptide, Manual IHC. Good Tumor staining.

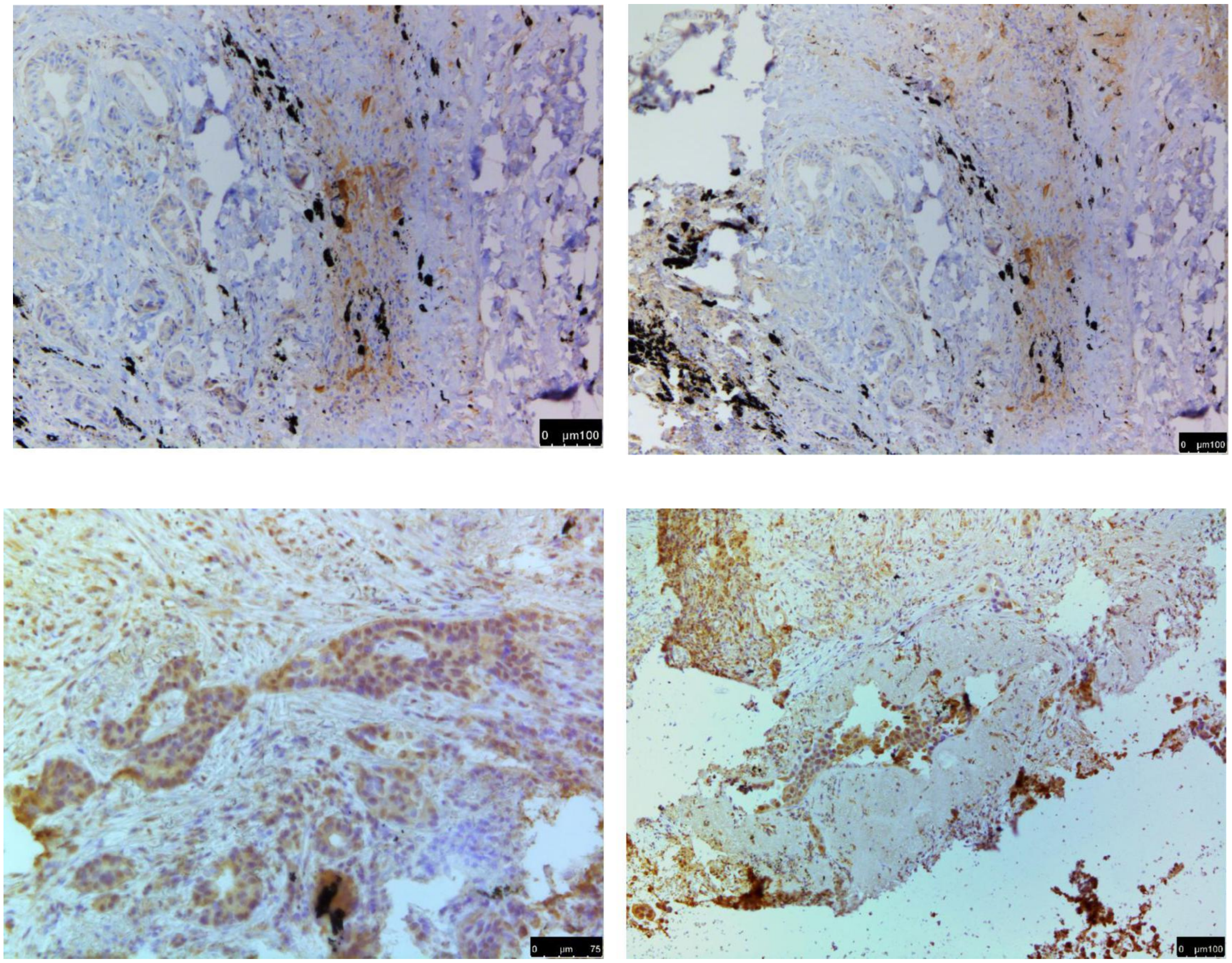

Figure S26: Patient D Stained with SP263 and RK-10-Biotin

SP263 Kit,
Autostained.
Faint/No
Tumor
staining

Peptide,
Manual IHC.
Good Tumor
staining.

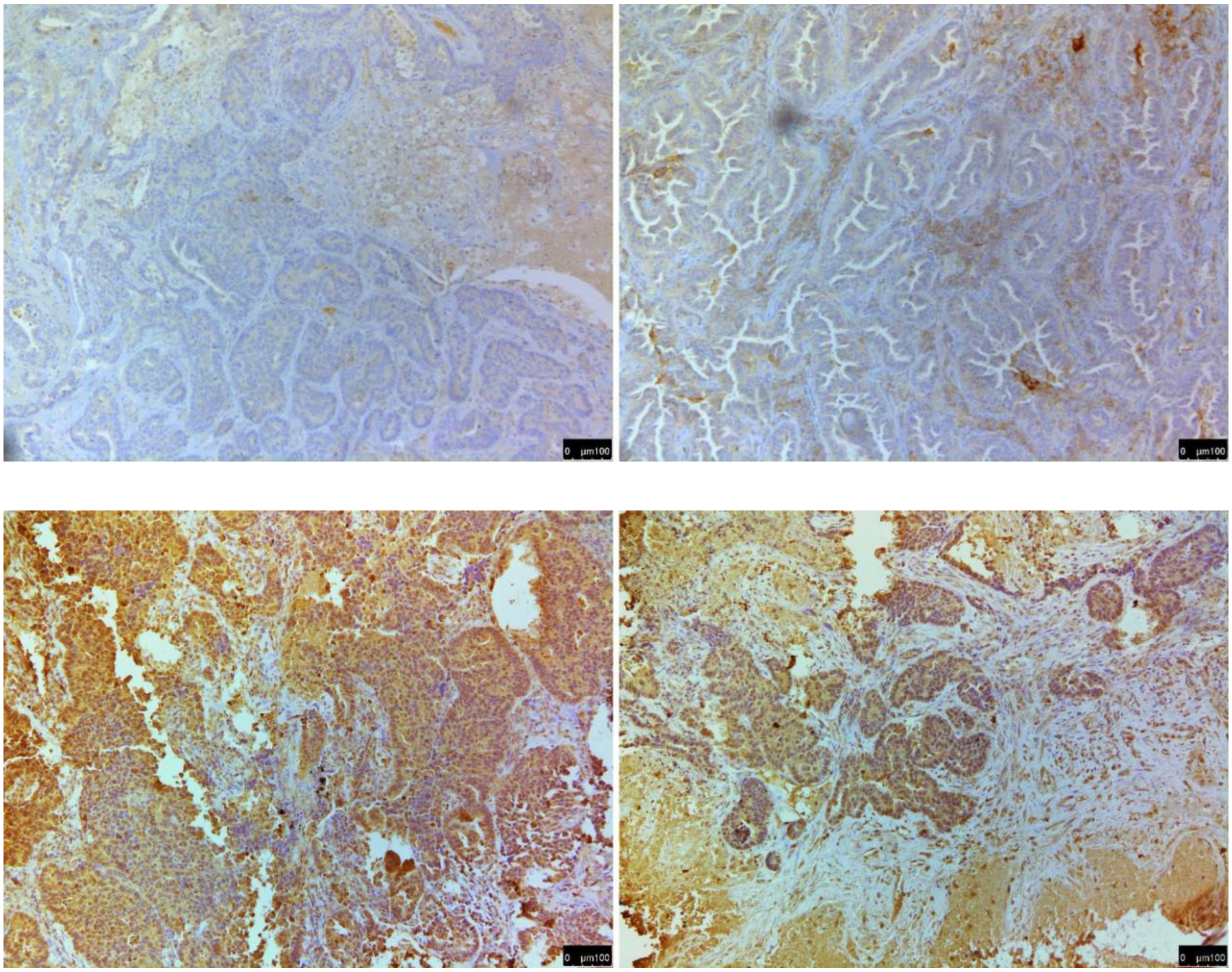

Figure S27. Patient E Stained with SP263 or RK-10-Biotin

SP263 Kit,
Autostained.
Very faint
staining seen

Peptide,
Manual IHC.
Tumor stains
heavily.

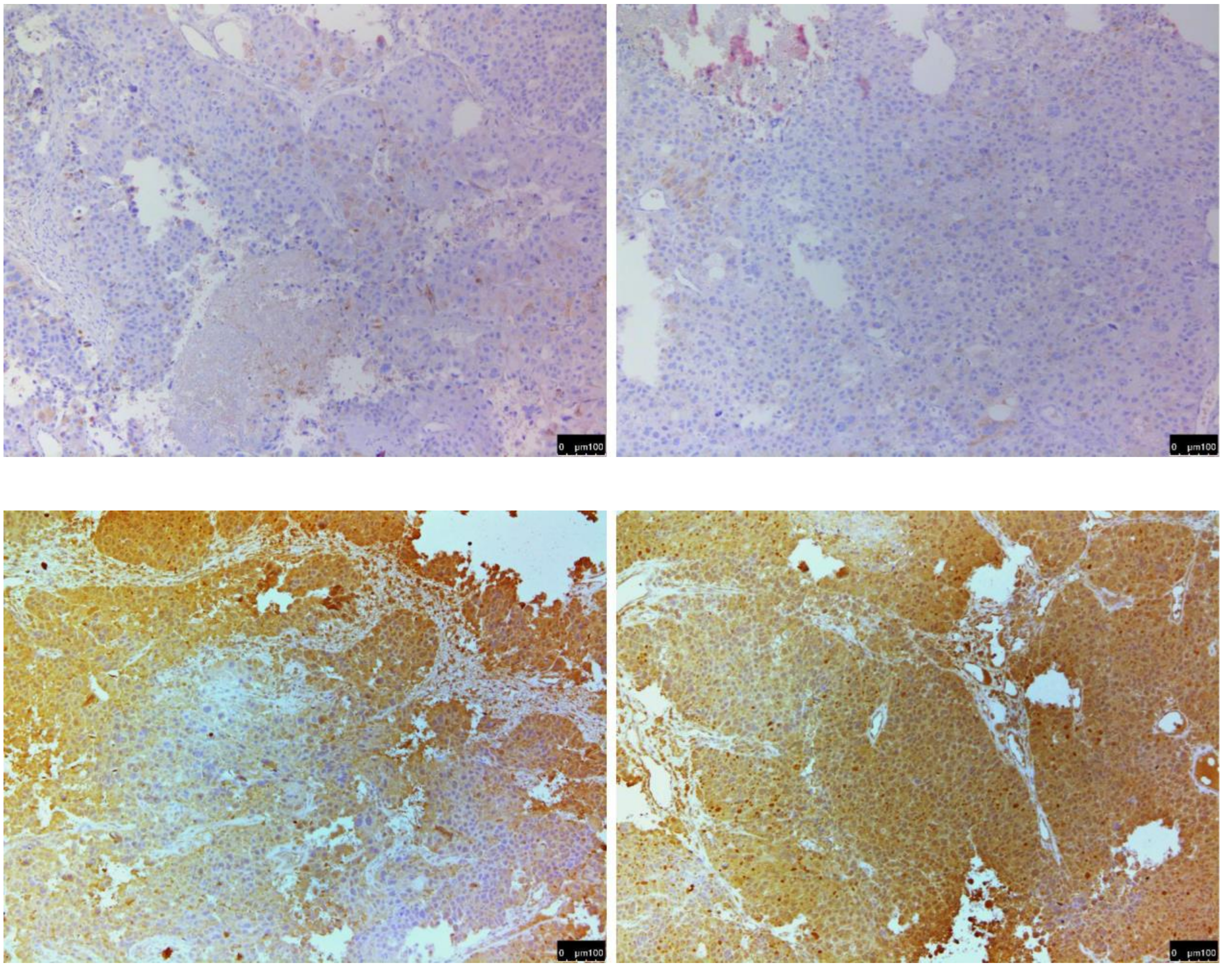

Figure S28. Patient F stained with SP263 and RK-10-Biotin

SP263 Kit, Autostained. Tumor Stains reliably

Peptide, Manual IHC. Tumor stains heavily.

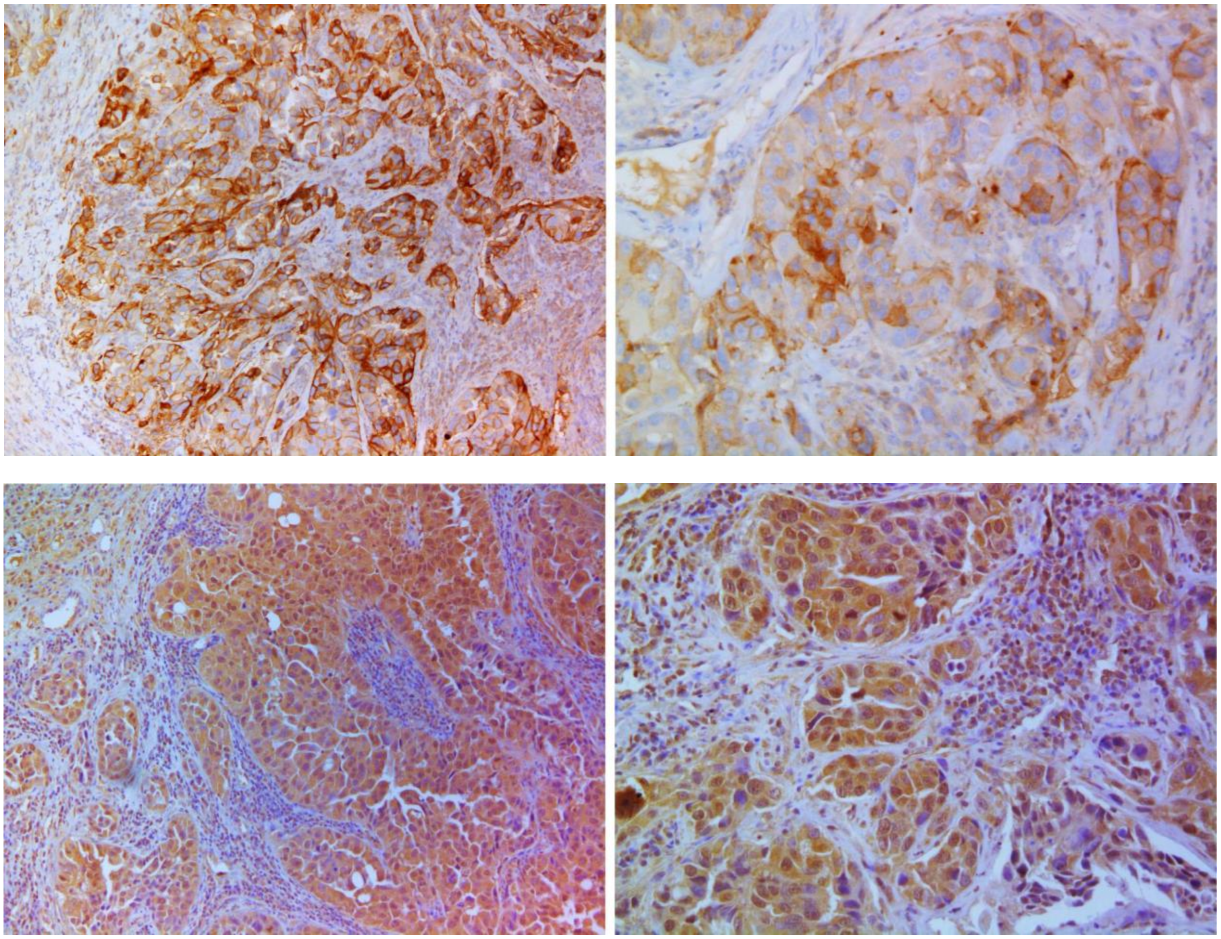

Figure S29. Patient G Stained with SP263 or RK-10-Biotin.

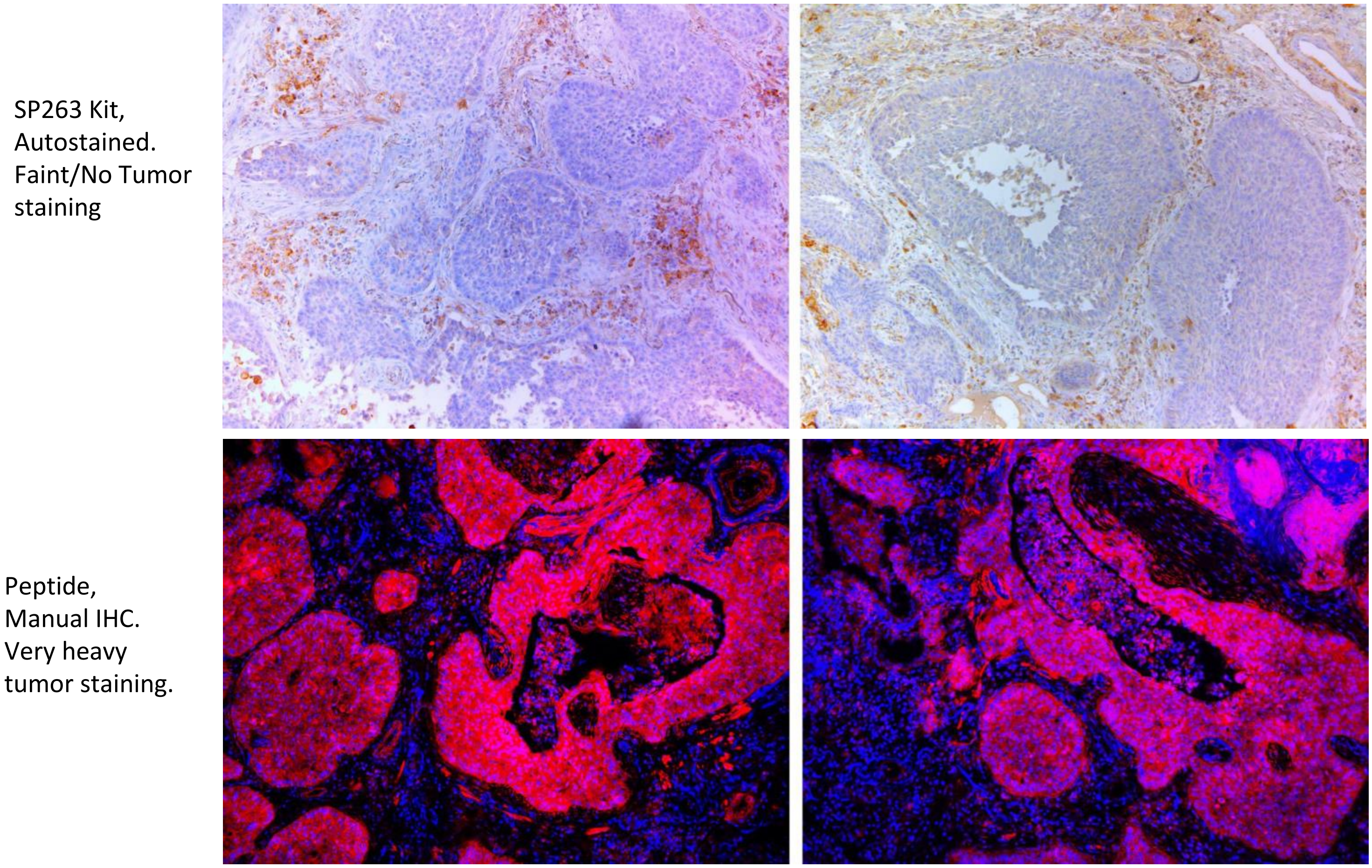

Figure S30 Patient A Stained with either SP263 or RK-10-Cy5

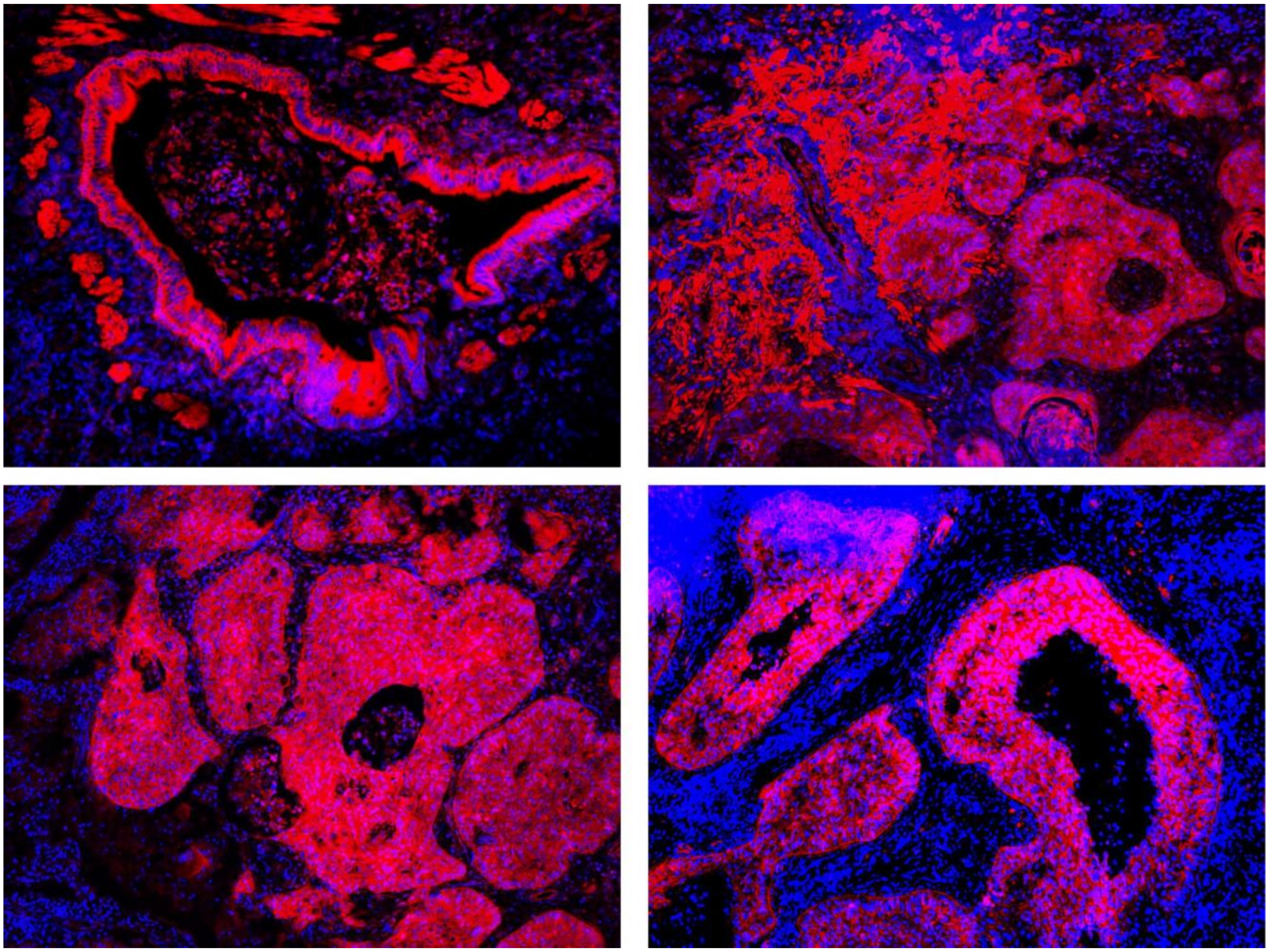

Figure S31 Patient A stained with RK-10-Cy5 clearly delineates tumor areas

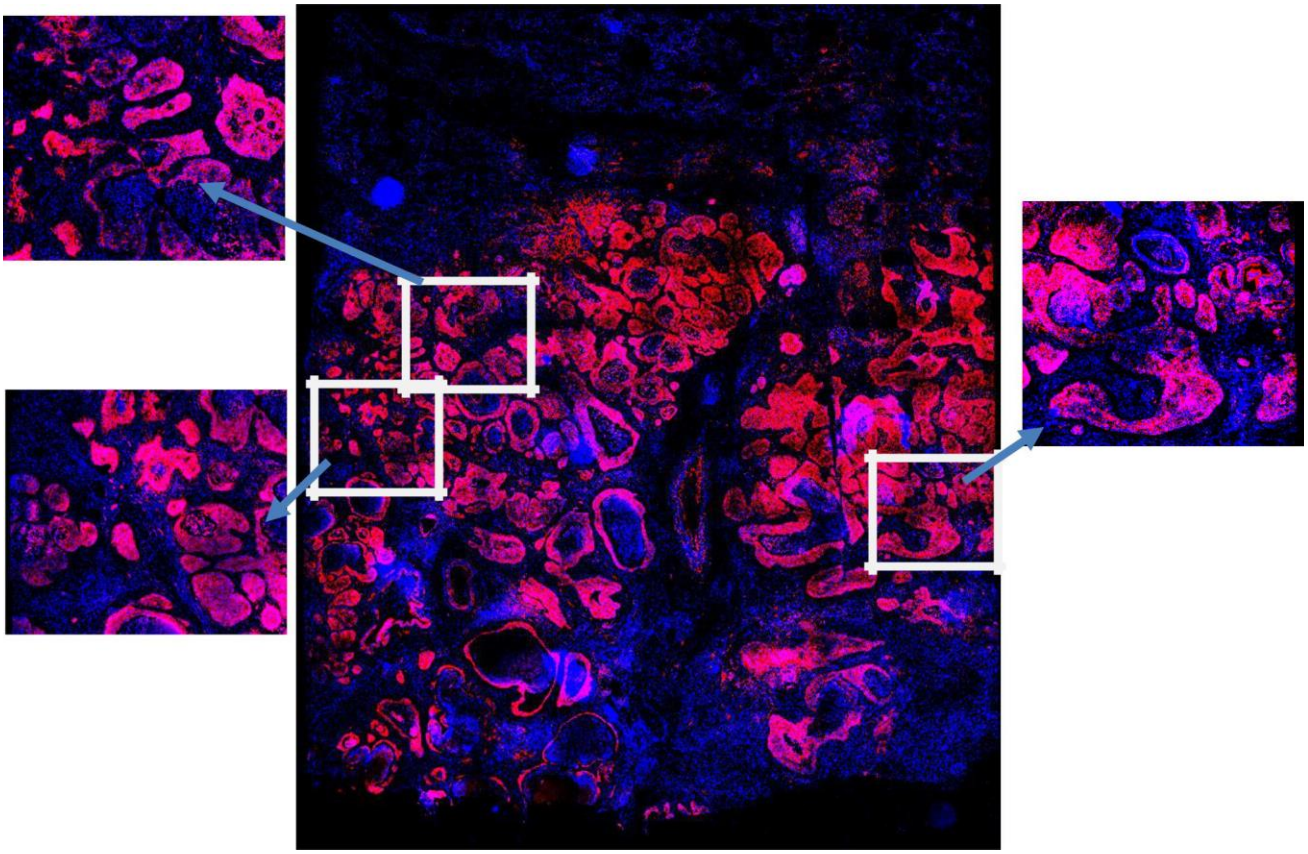

Figure S32 Whole slide scan of Patient A stained with RK-10-Cy5 identifies tumor areas

SP263 Kit,
Autostained.
Faint/No Tumor
staining

Peptide,
Manual IHC

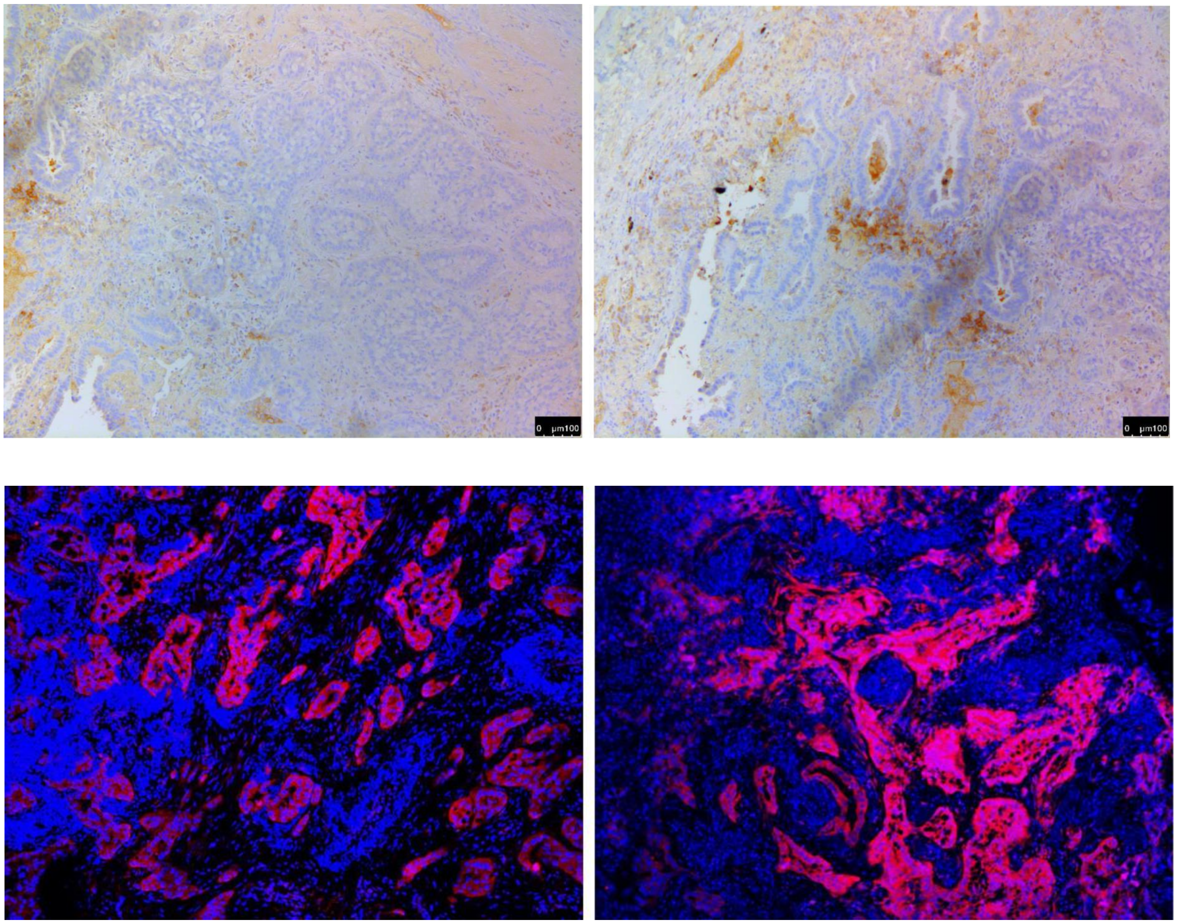

Figure S33. Patient B Stained with SP263 or RK-10-Cy5

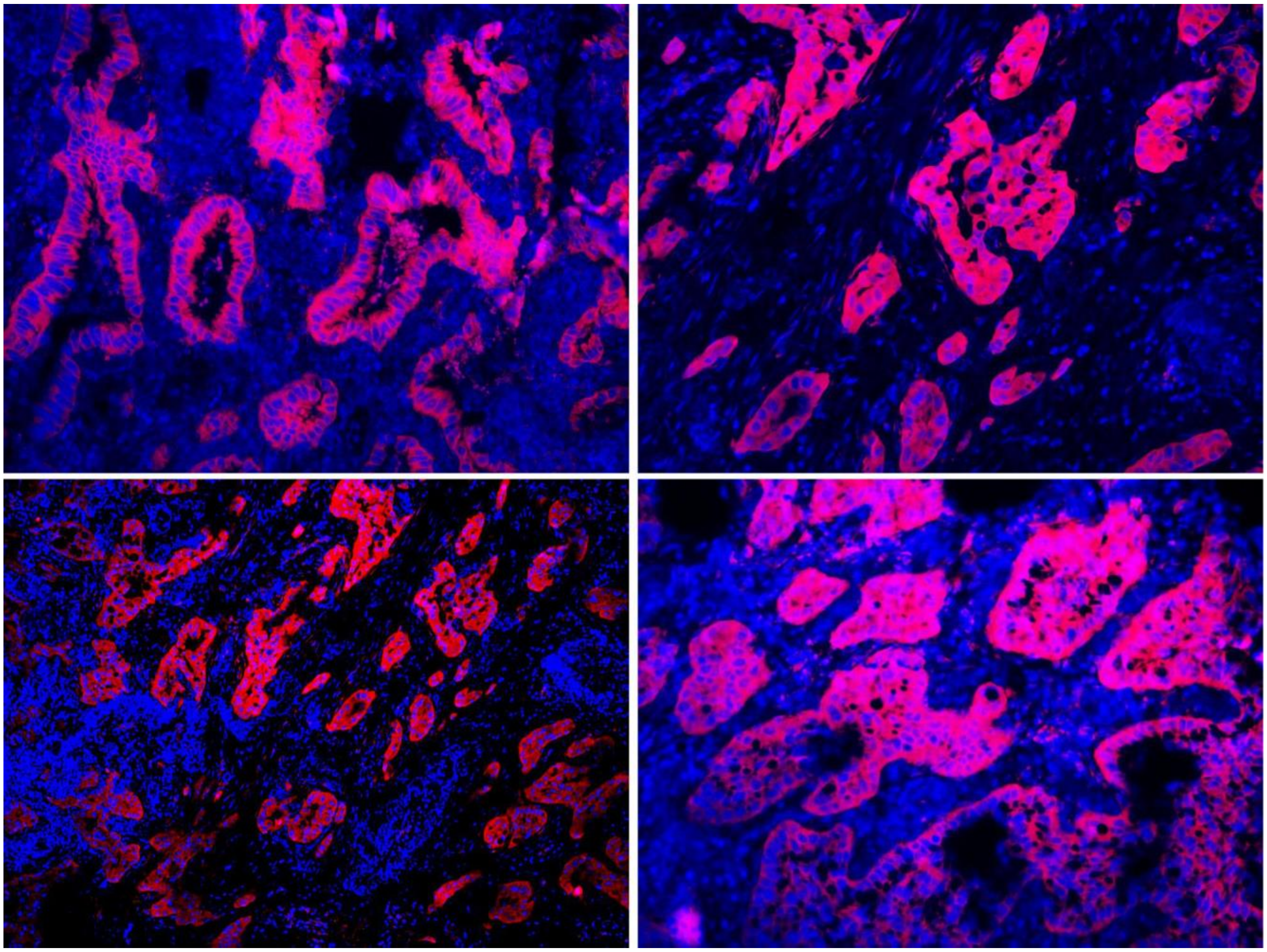

Figure S34. Patient B Stained with RK-10-Cy5 clearly delineates tumor regions

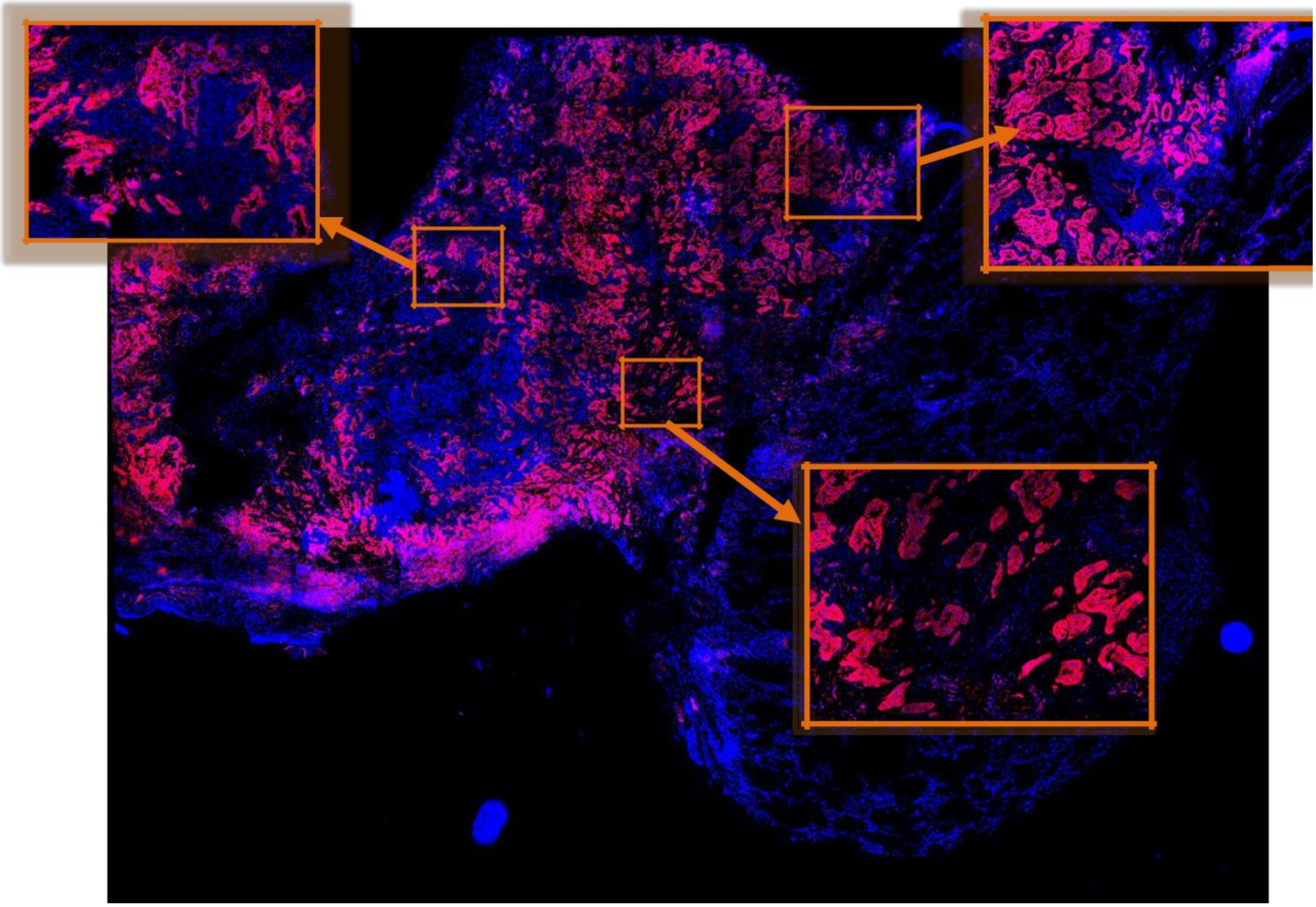

Figure S35 Whole slide scan of patient B Stained with RK-10-Cy5 clearly identifies tumor regions

SP263 Kit,
Autostained.
Faint/No
Tumor staining

Peptide,
Manual IHC

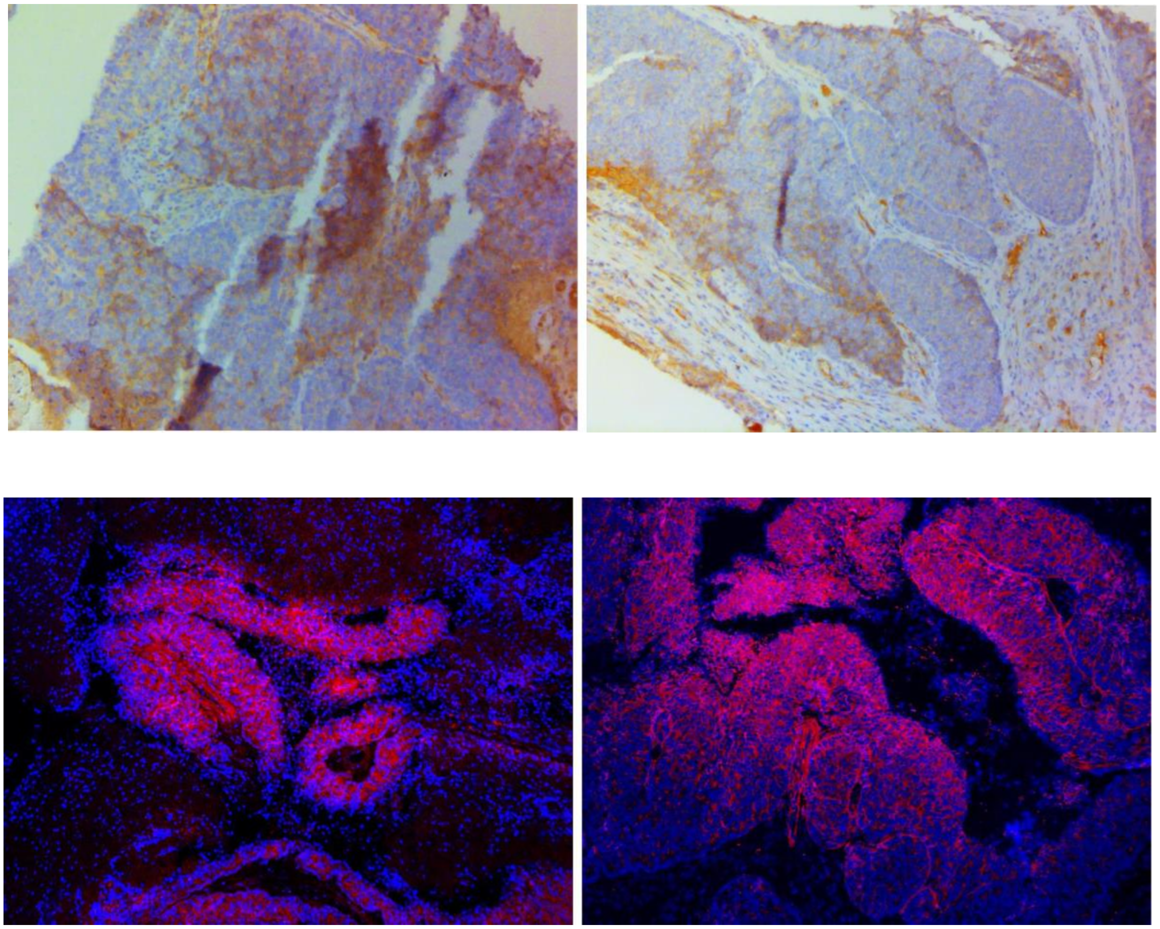

Figure S36 Patient C Stained with SP263 or RK-10-Cy5

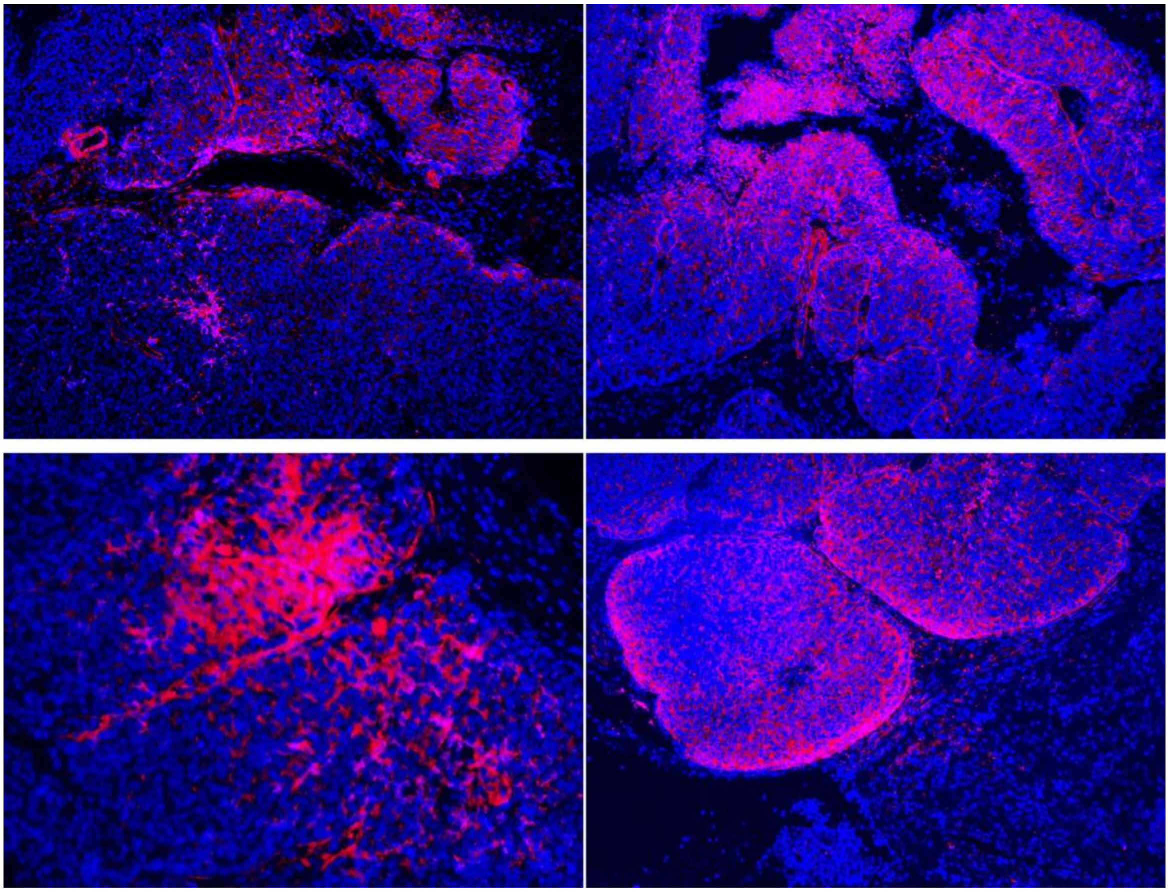

Figure S37 Patient C Stained with RK-10-Cy5 stains some regions of the tumor

SP263 Kit,

Autostained.

Peptide,
Manual IHC

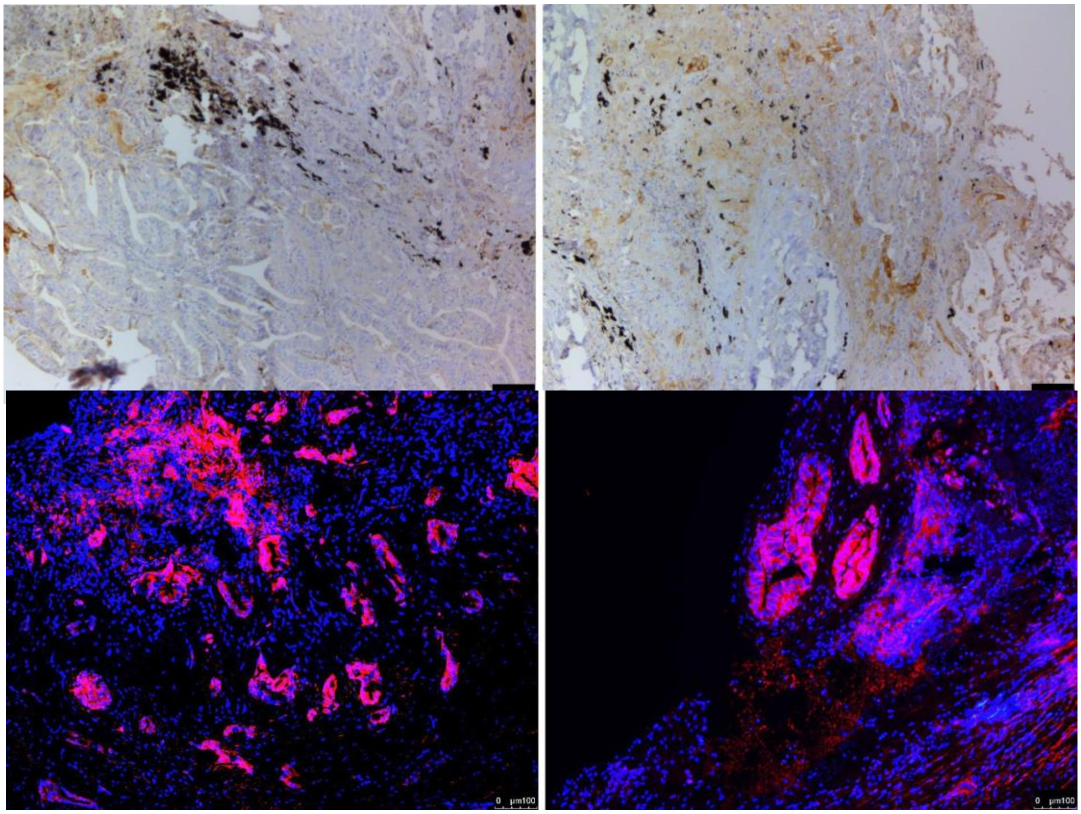

Figure S38 Patient D Stained with SP263 or RK-10-Cy5

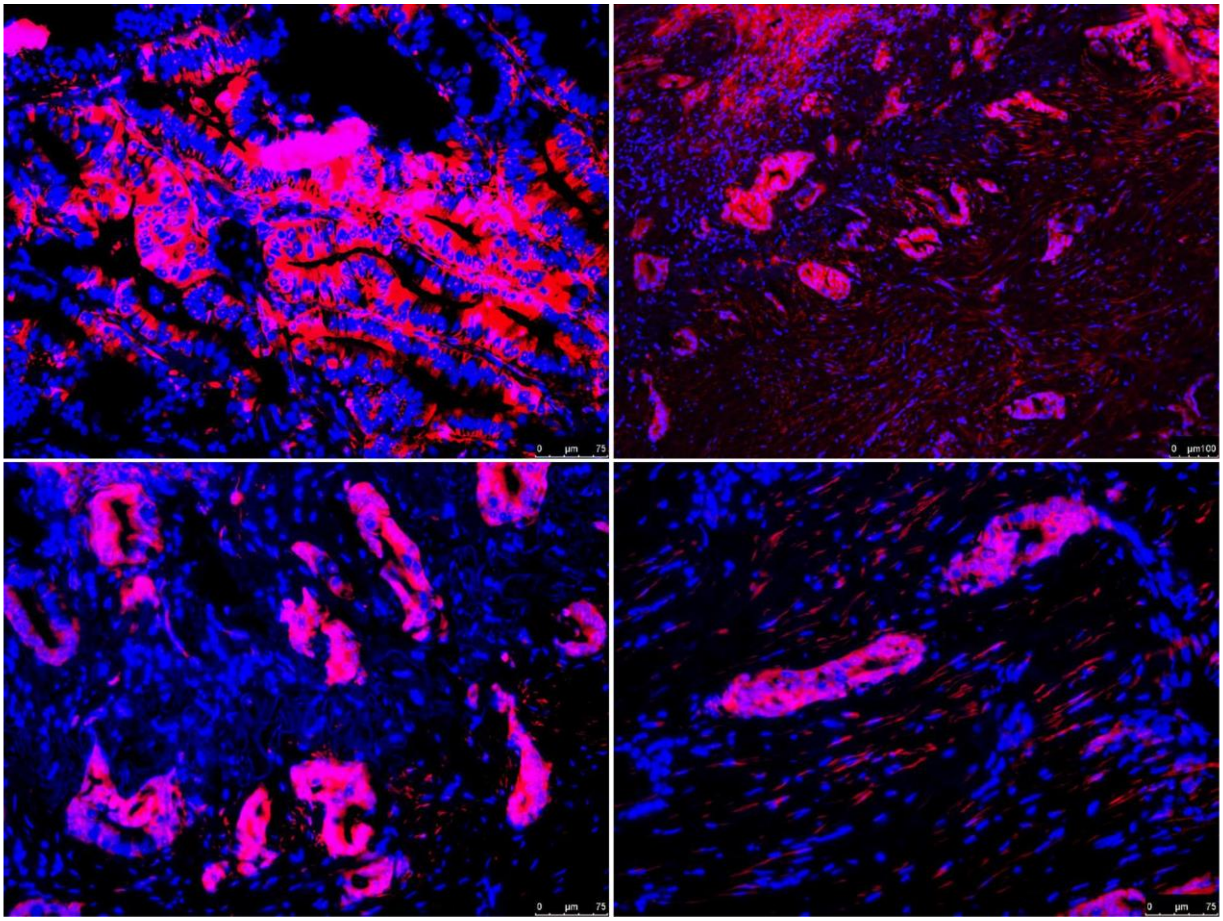

Figure S39 Patient D Stained with RK-10-Cy5

SP263 Kit,
Autostained.
Faint/No Tumor
staining

Peptide,
Manual IHC

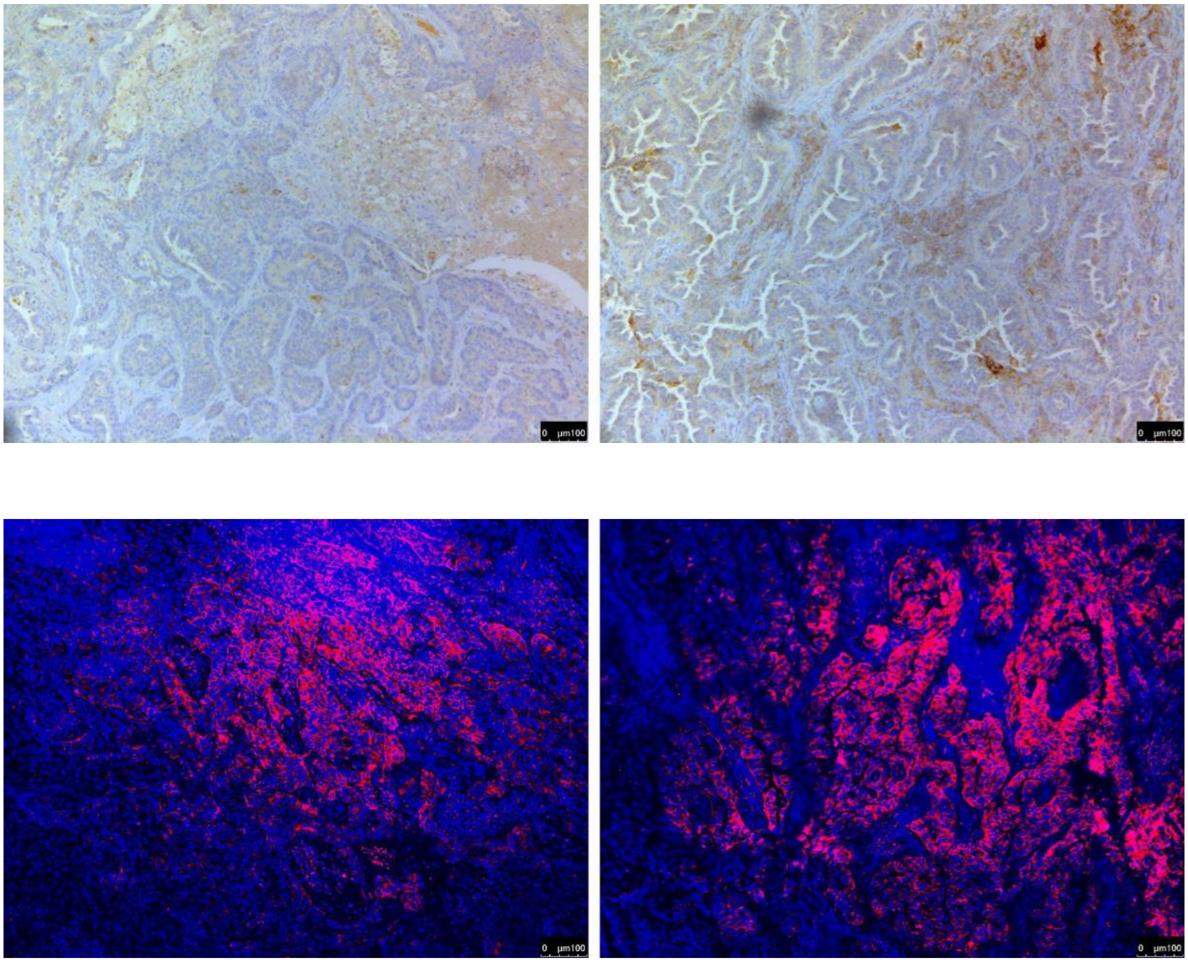

Figure S40 Patient E stained with SP263 or RK-10-Cy5

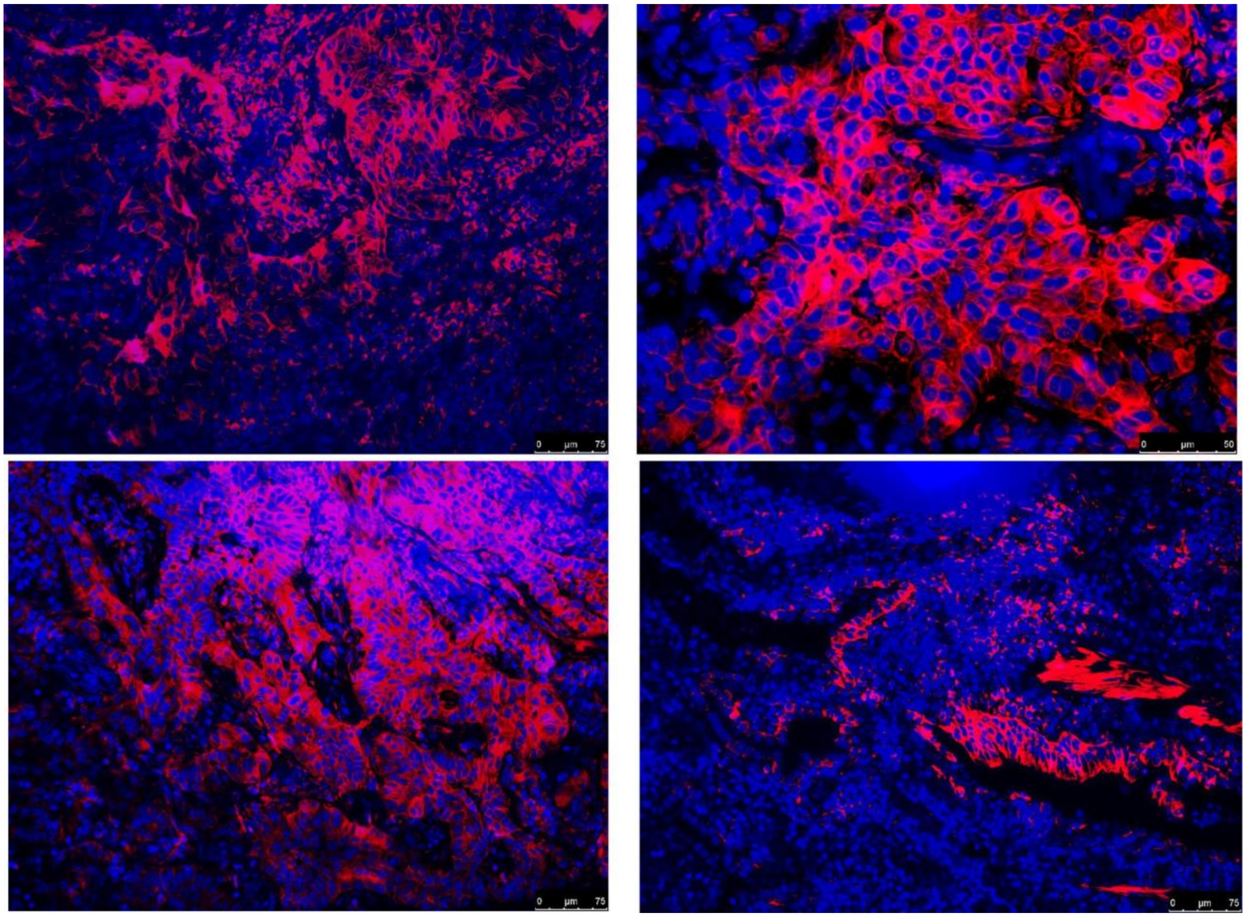

Figure S41 Patient E Stained with RK-10-Cy5

SP263 Kit,
Autostained.

Peptide,
Manual IHC

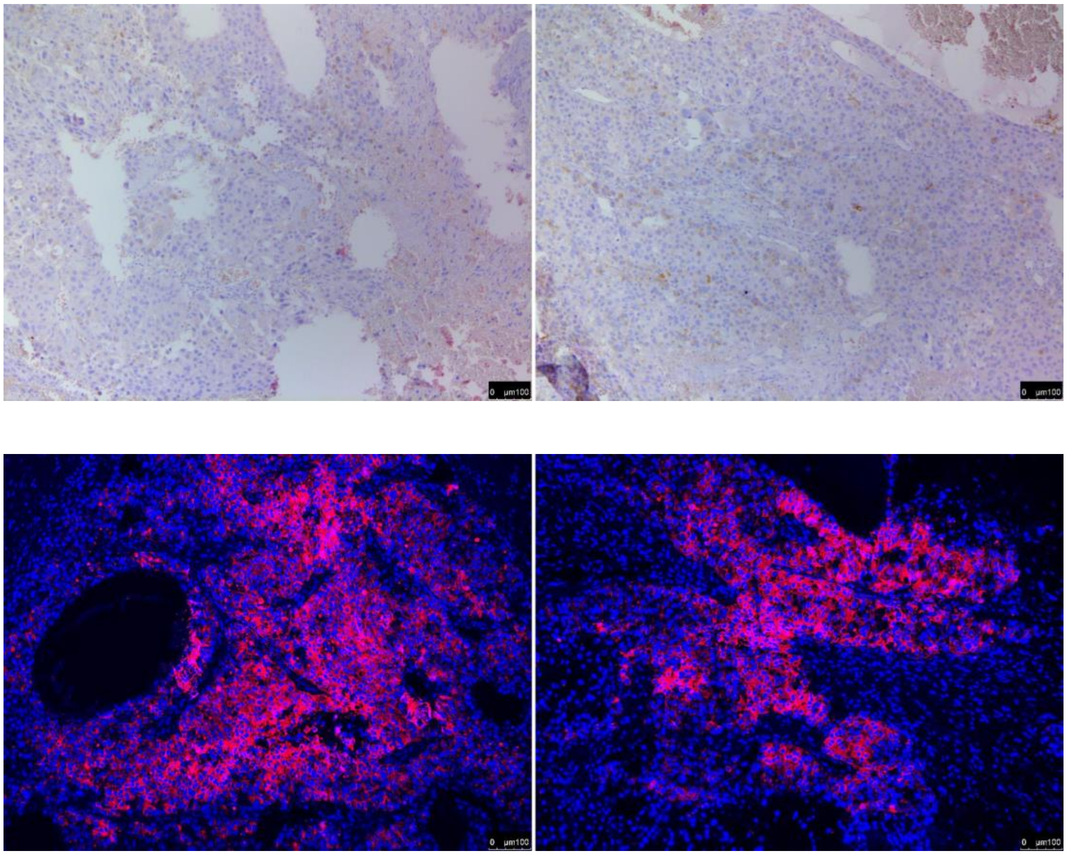

Figure S42 Patient F stained with SP263 or RK-10-Cy5

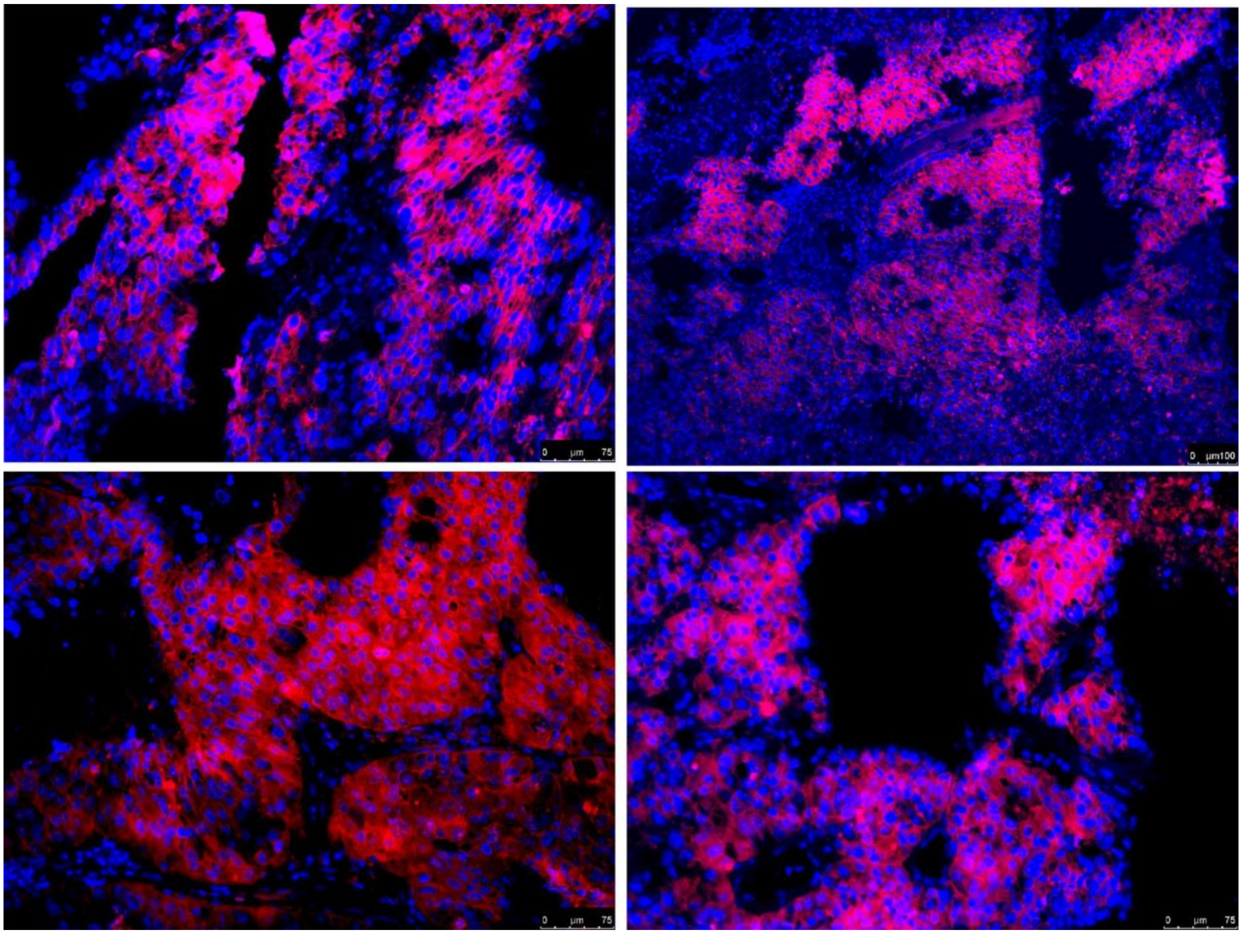

Figure S43 Patient F Stained with RK-10-Cy5

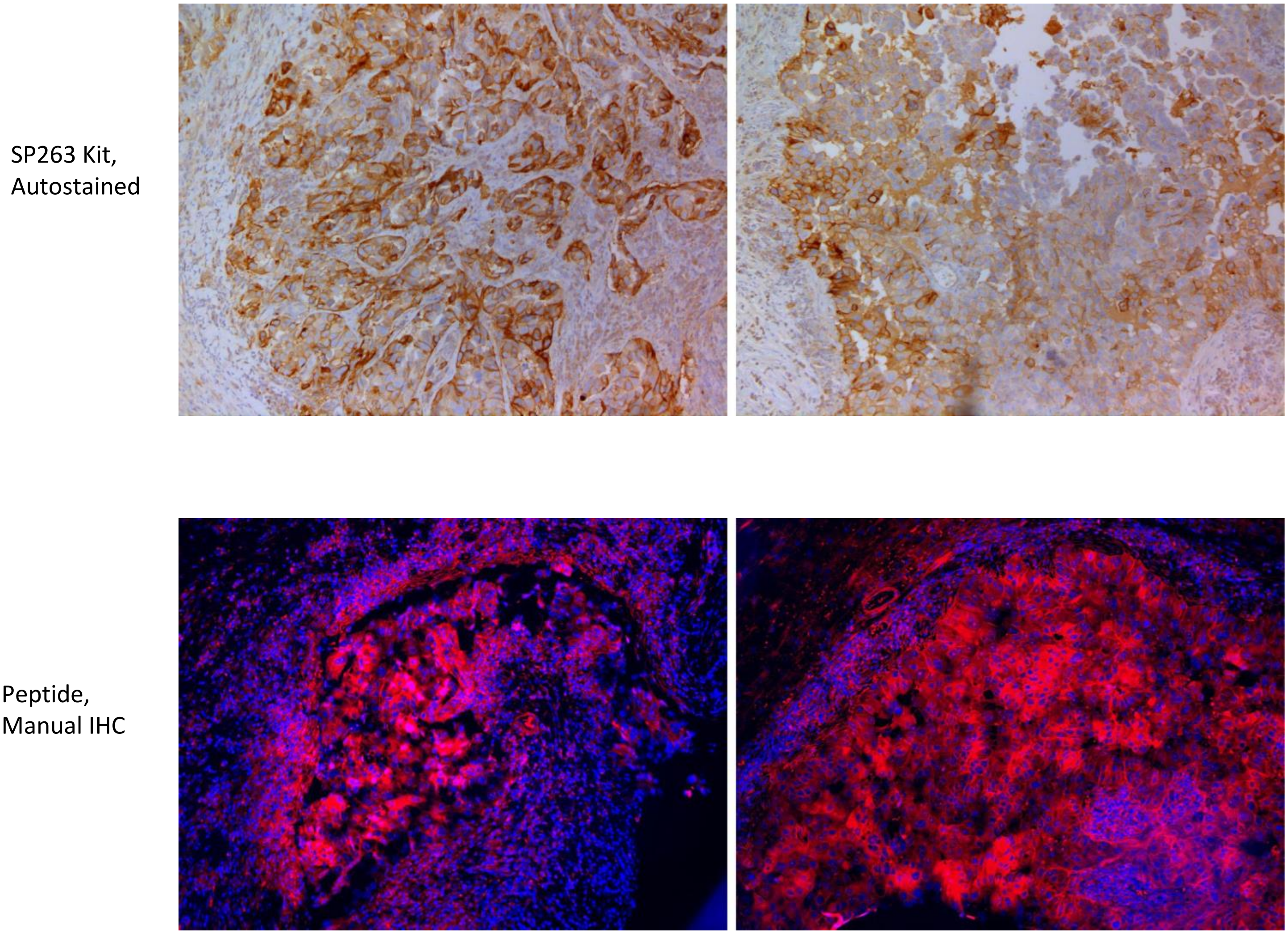

Figure S44 Patient G Stained with SP263 or RK-10-Cy5

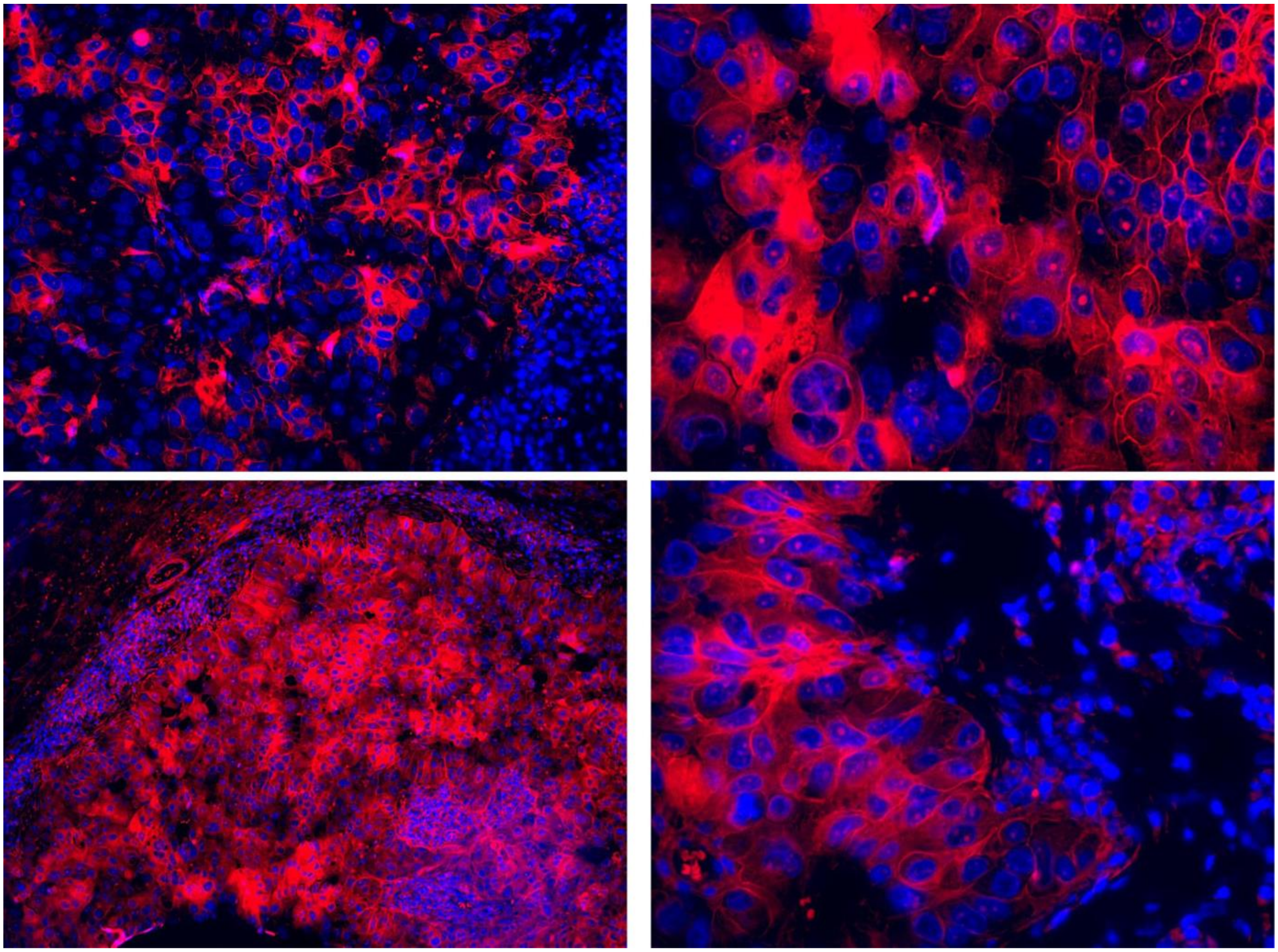

Figure S45 Patient G Stained with RK-10-Cy5

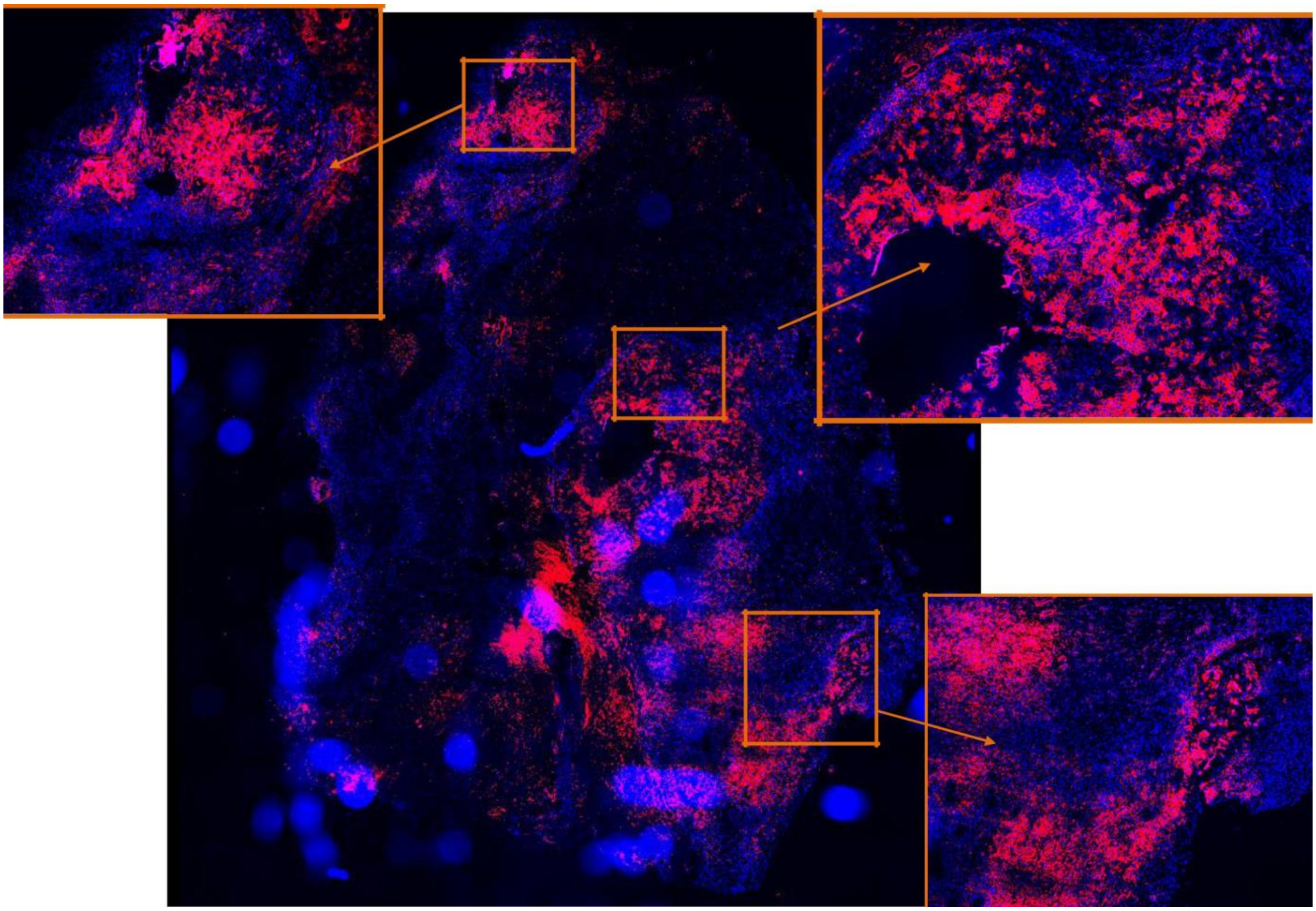

Figure S46 Whole slide scan of Patient G stained with RK-10-Cy5 shows tumor areas clearly highlighted by PD-L1 expression

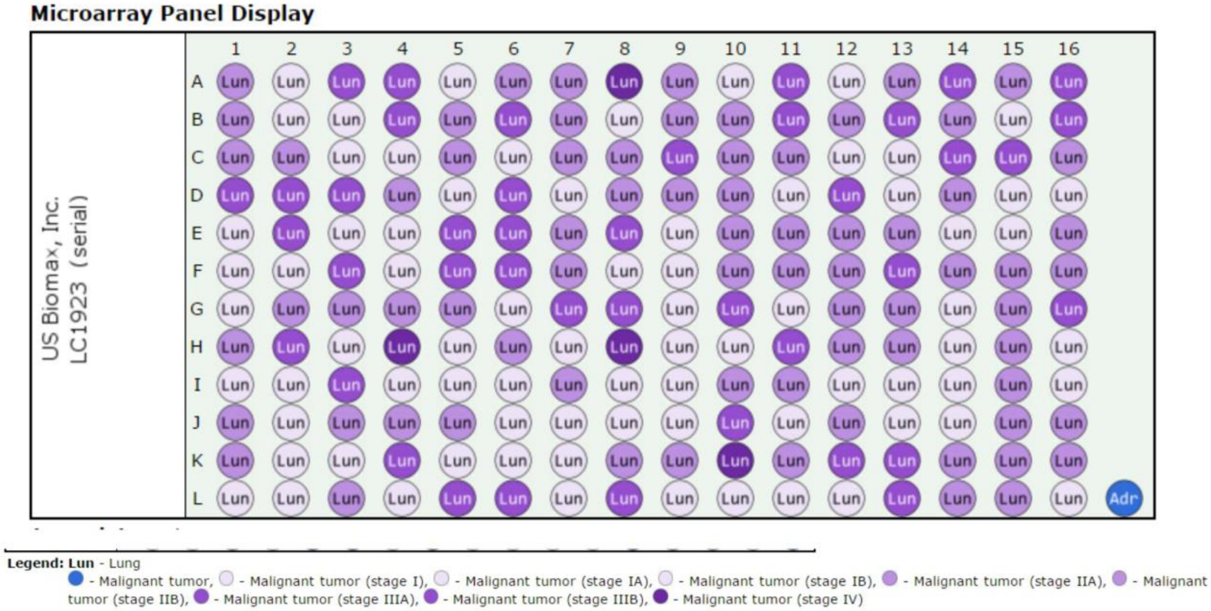

Figure S47 TMA obtained from US Biomax that was stained with RK-10-Cy5 or SP263

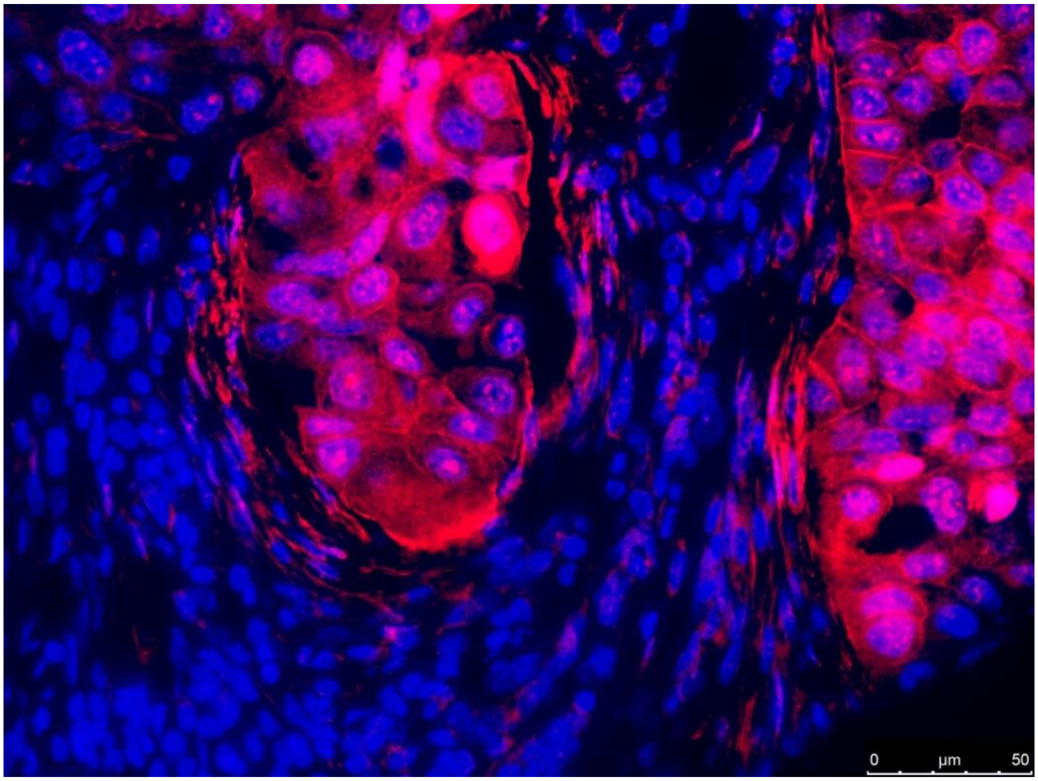

Figure S48 40X image of tumor area in TMA stained with RK-10-Cy5 shows specific staining of tumor for PD-L1 expression

S49: Double Negative Spot

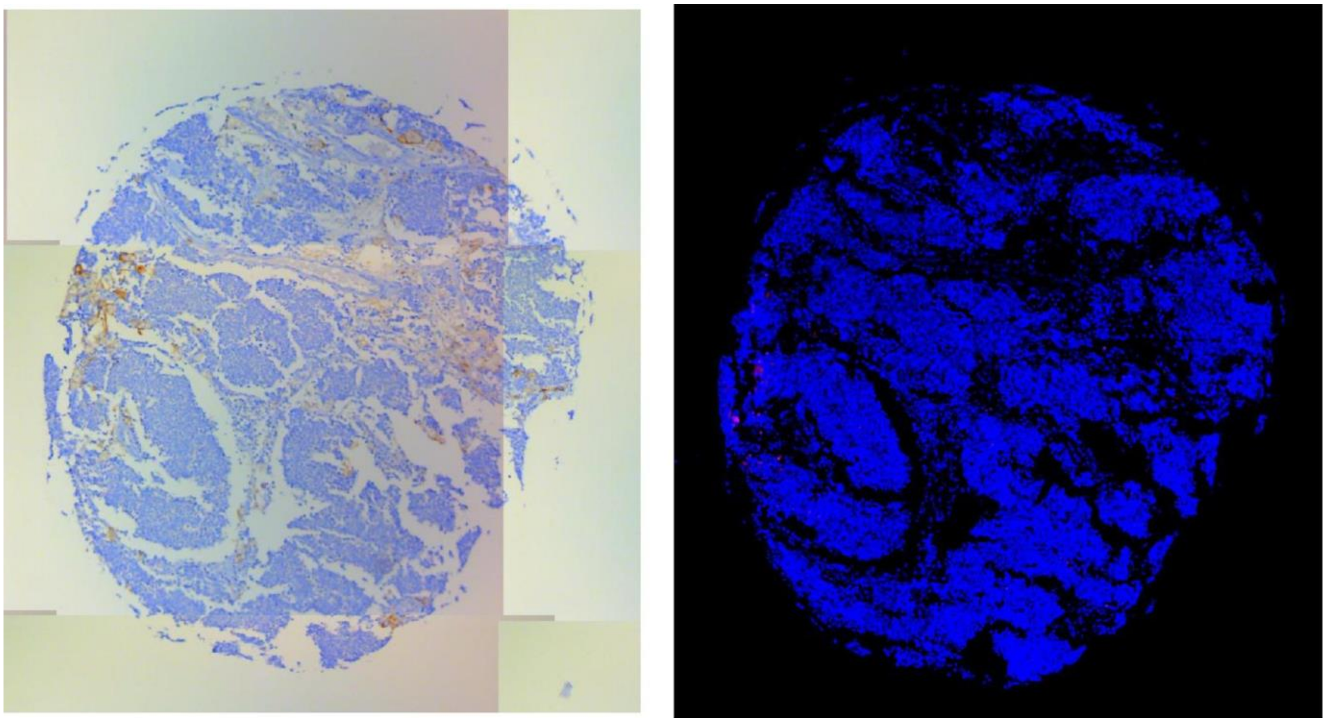

Figure S49. TMA Stained with SP263 (left) and RK-10-Cy5 (right) shows negative signal from both methods

S50: Double Positive Spot

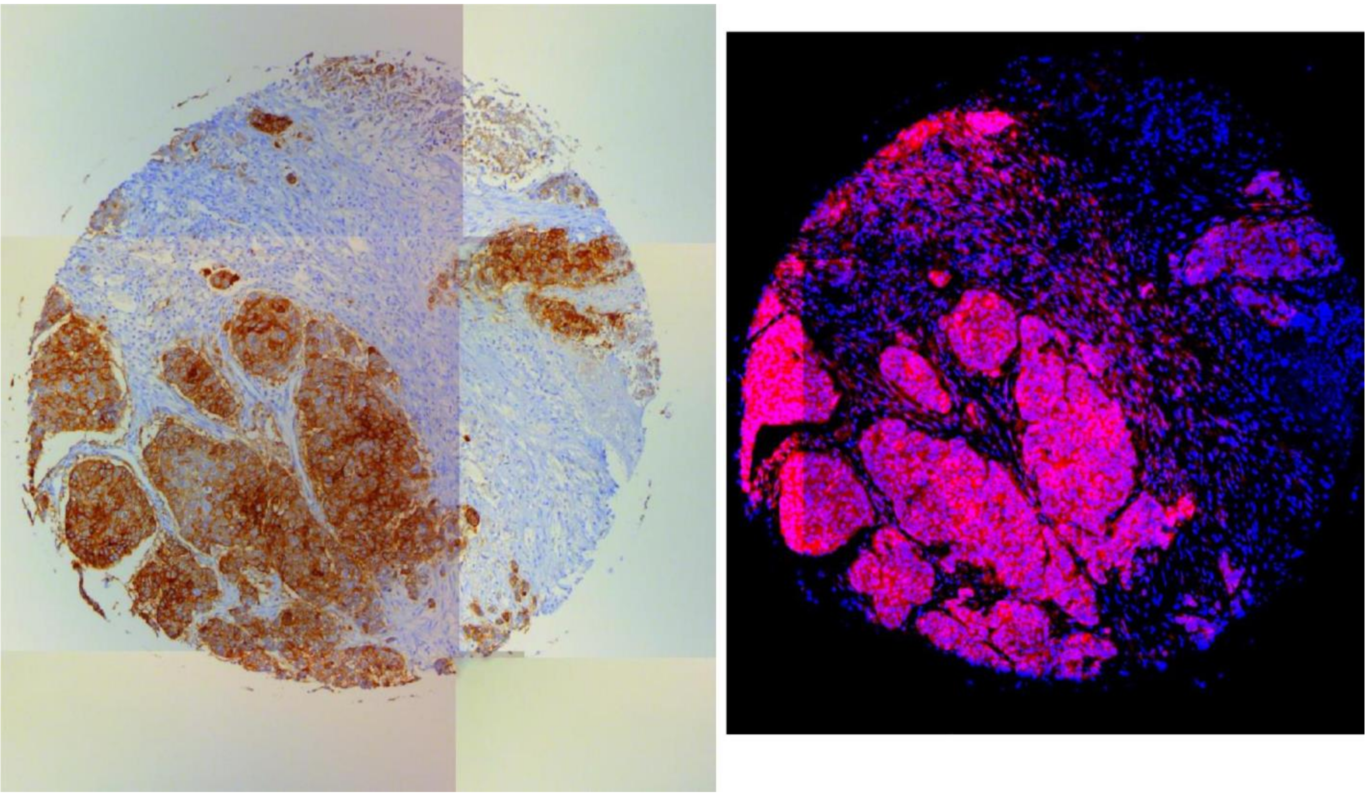

Figure S50. TMA Stained with SP263 (left) and RK-10-Cy5 (right) shows positive signal from both methods

S51: Peptide Positive, SP263 Negative

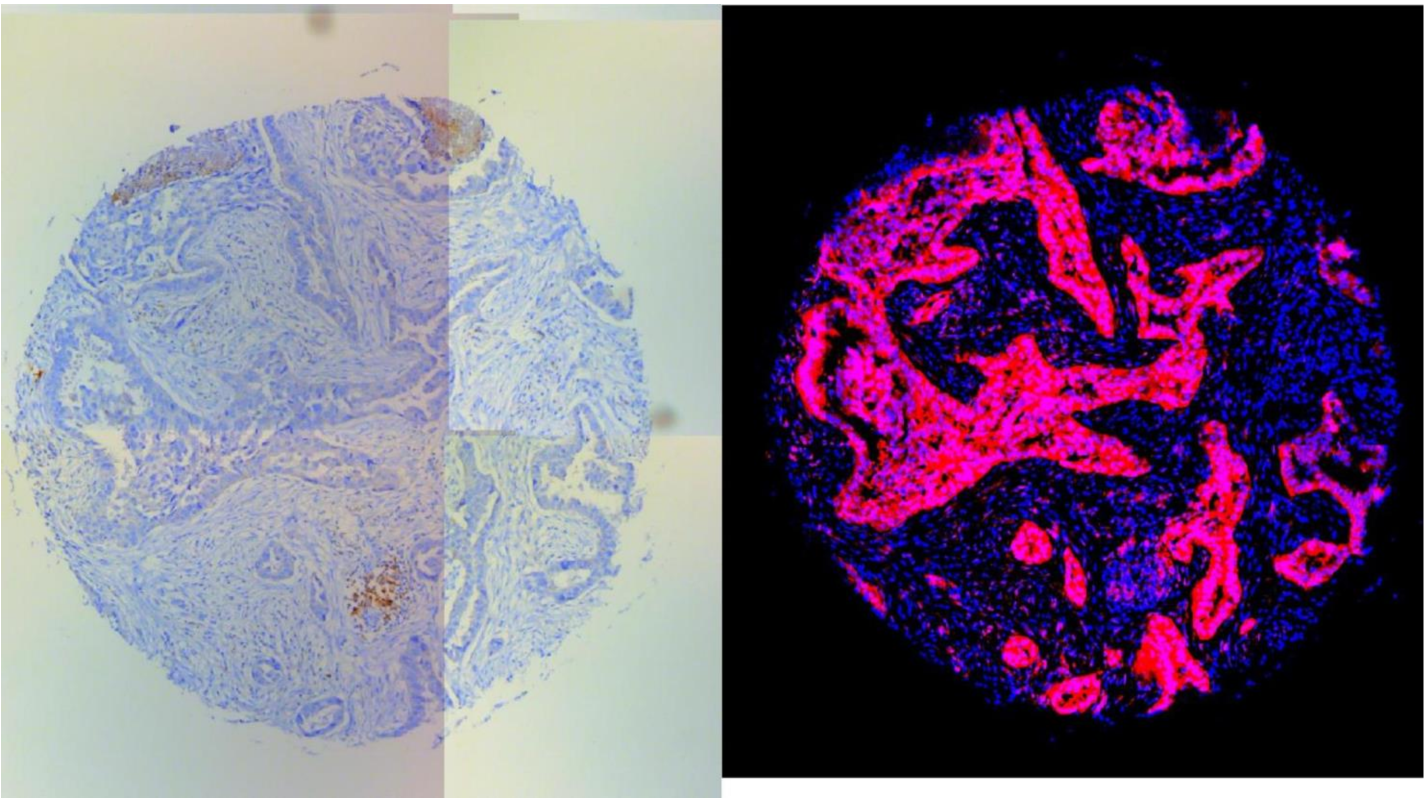

Figure S51. TMA Stained with SP263 (left) and RK-10-Cy5 (right) shows negative signal from SP263 and positive stain using the RK-10-Cy5 peptide.

S52: Double Positive Spot

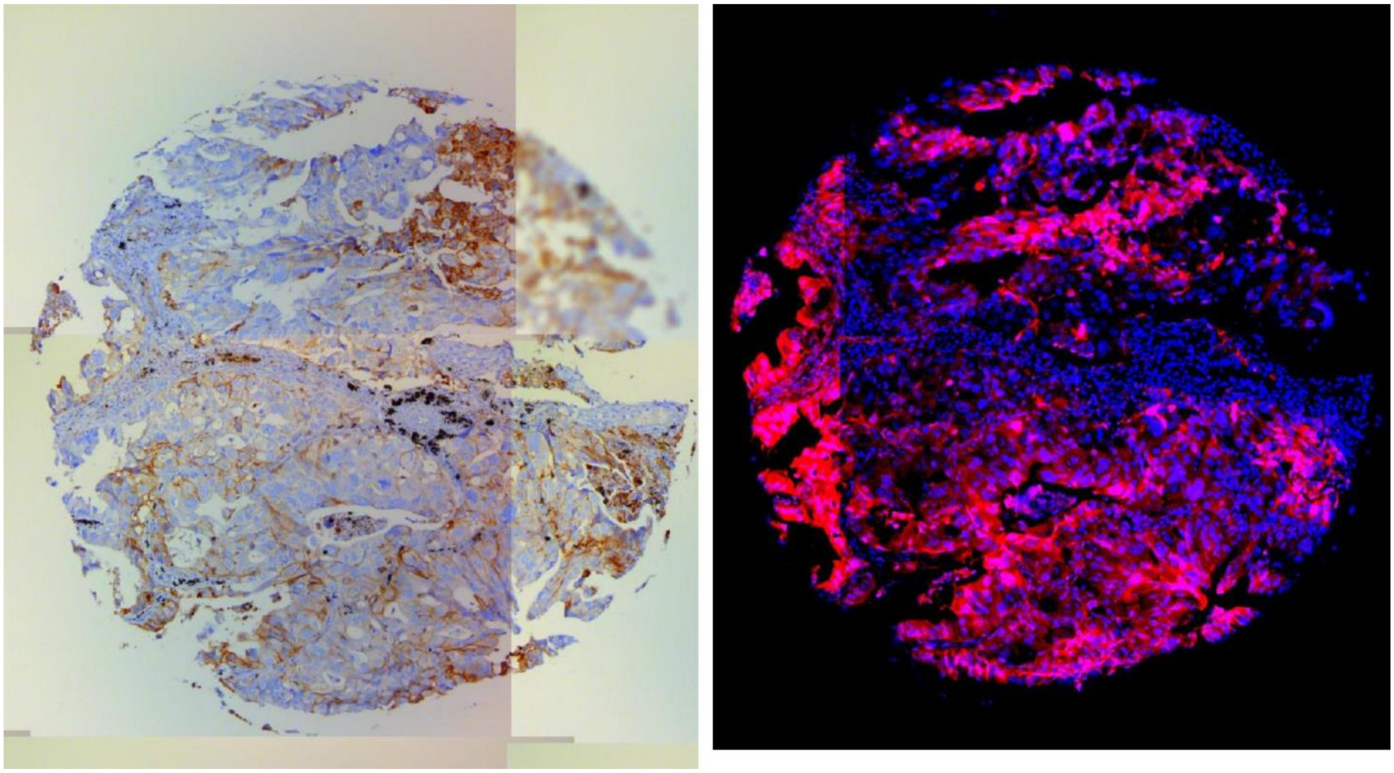

Figure S52. TMA Stained with SP263 (left) and RK-10-Cy5 (right) shows positive signal from both methods

S53: Double Positive Spot

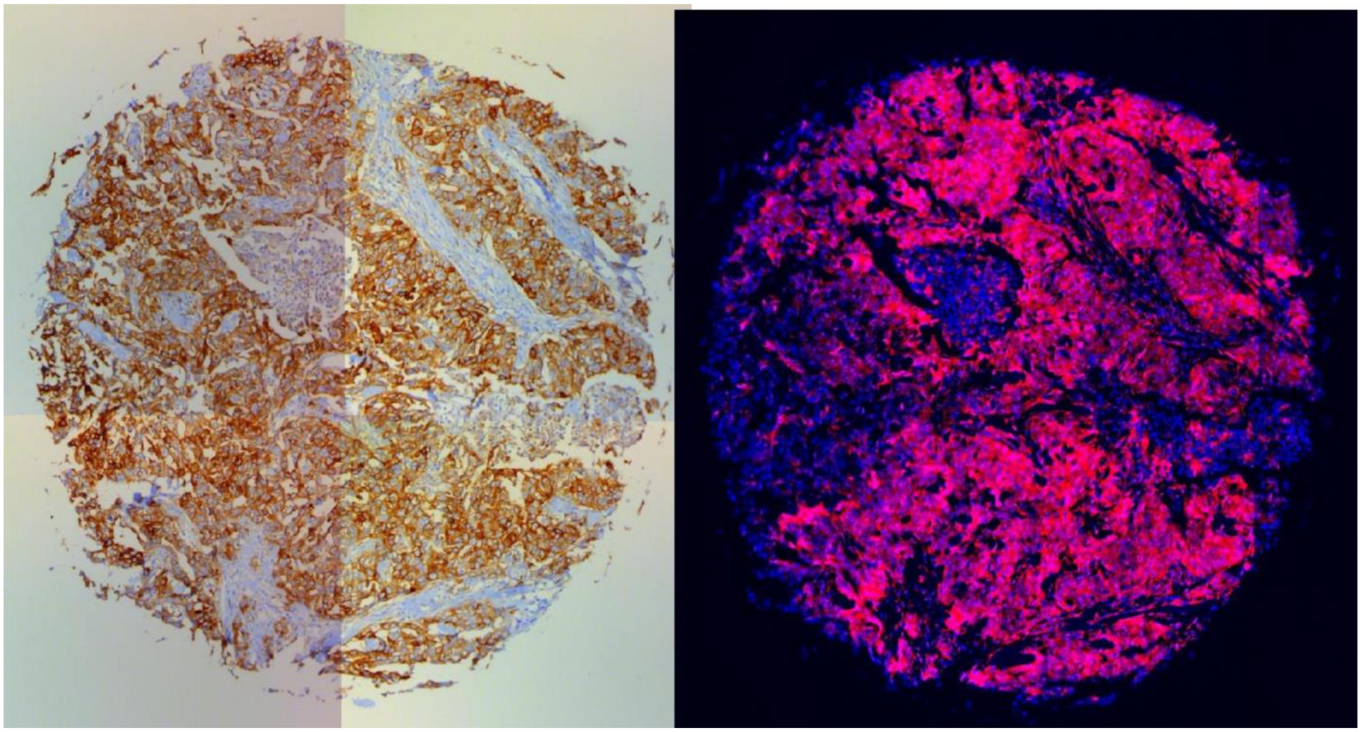

Figure S53. TMA Stained with SP263 (left) and RK-10-Cy5 (right) shows positive signal from both methods

S54: Double Positive Spot

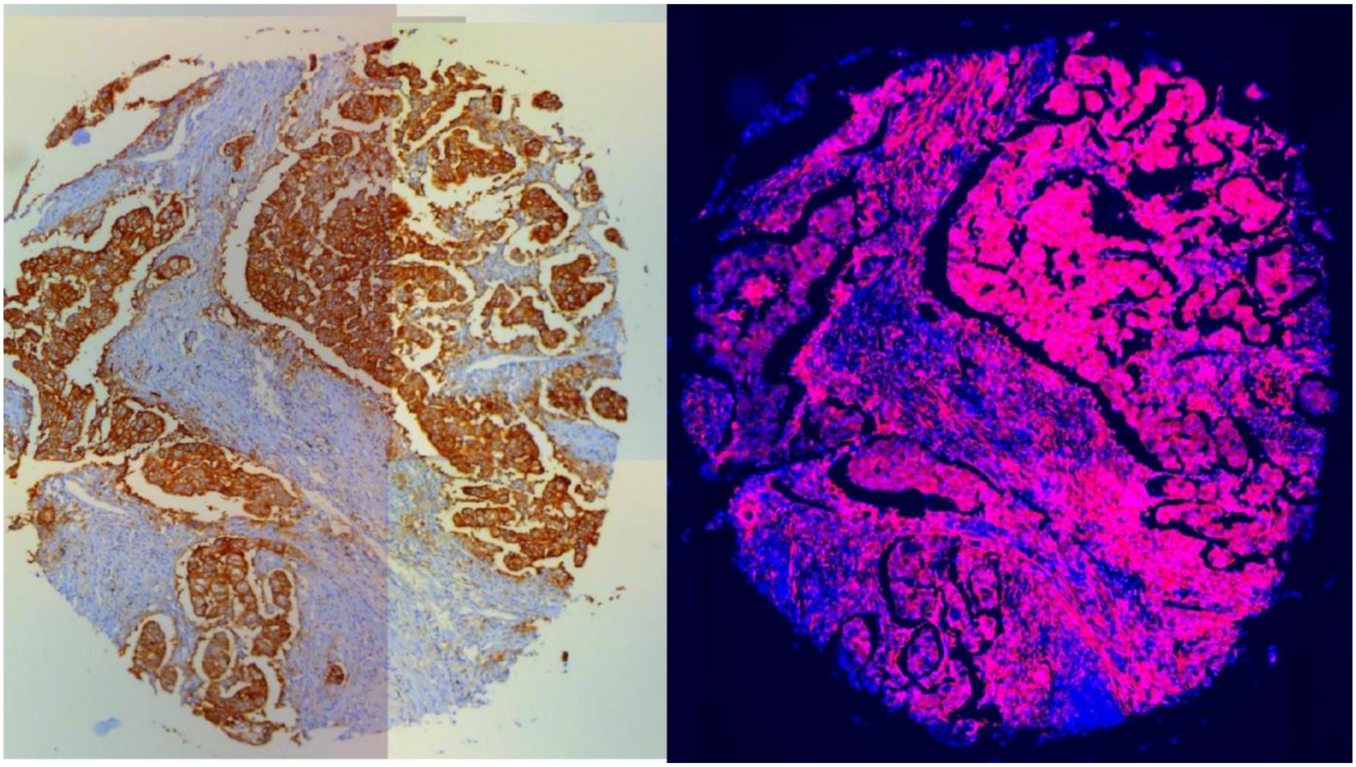

Figure S54. TMA Stained with SP263 (left) and RK-10-Cy5 (right) shows positive signal from both methods

S55: Peptide Positive, SP263 Negative

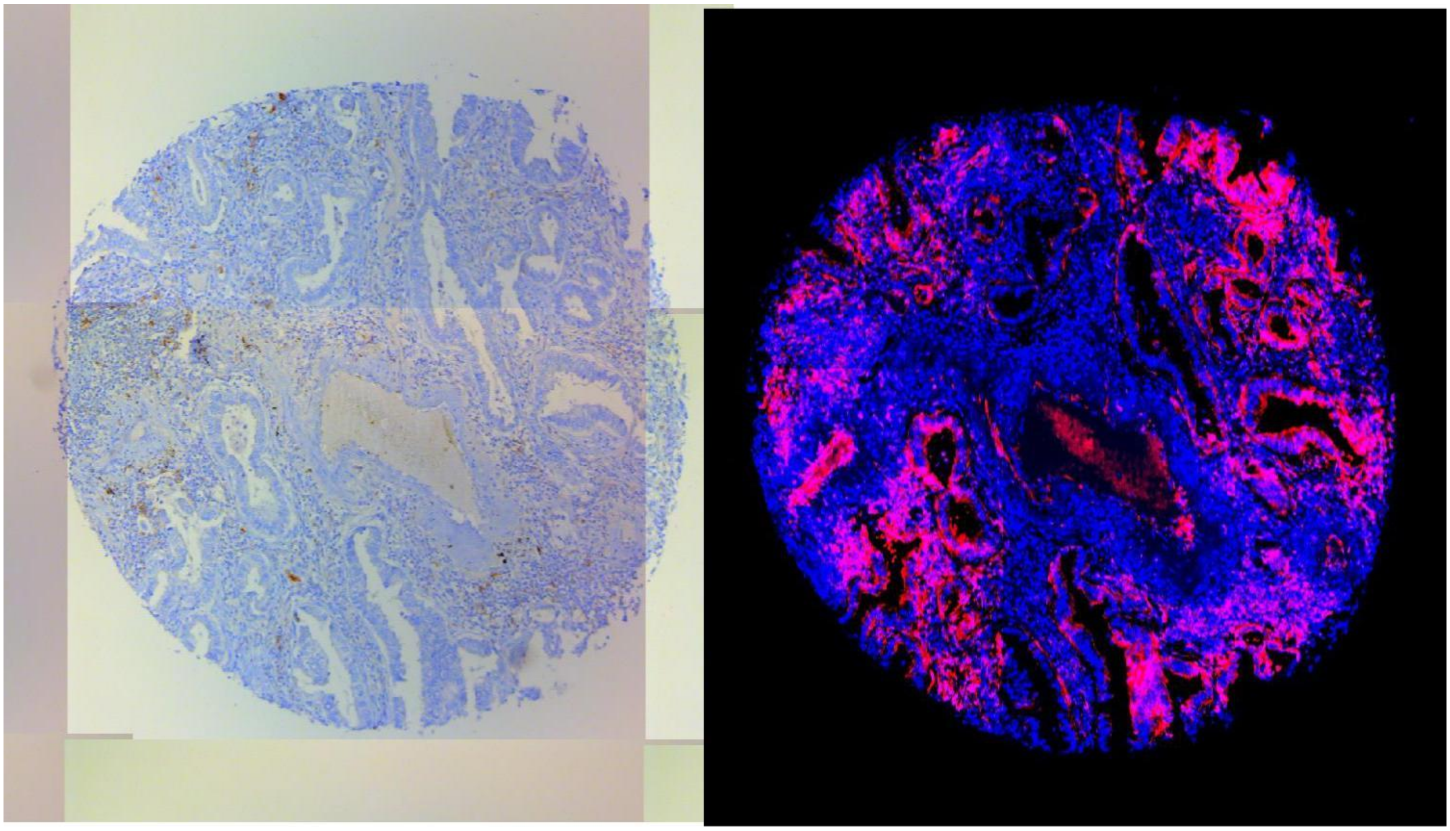

Figure S55. TMA Stained with SP263 (left) and RK-10-Cy5 (right) shows negative signal from SP263 and positive stain using the RK-10-Cy5 peptide.

S56: Peptide Positive, SP263 Negative

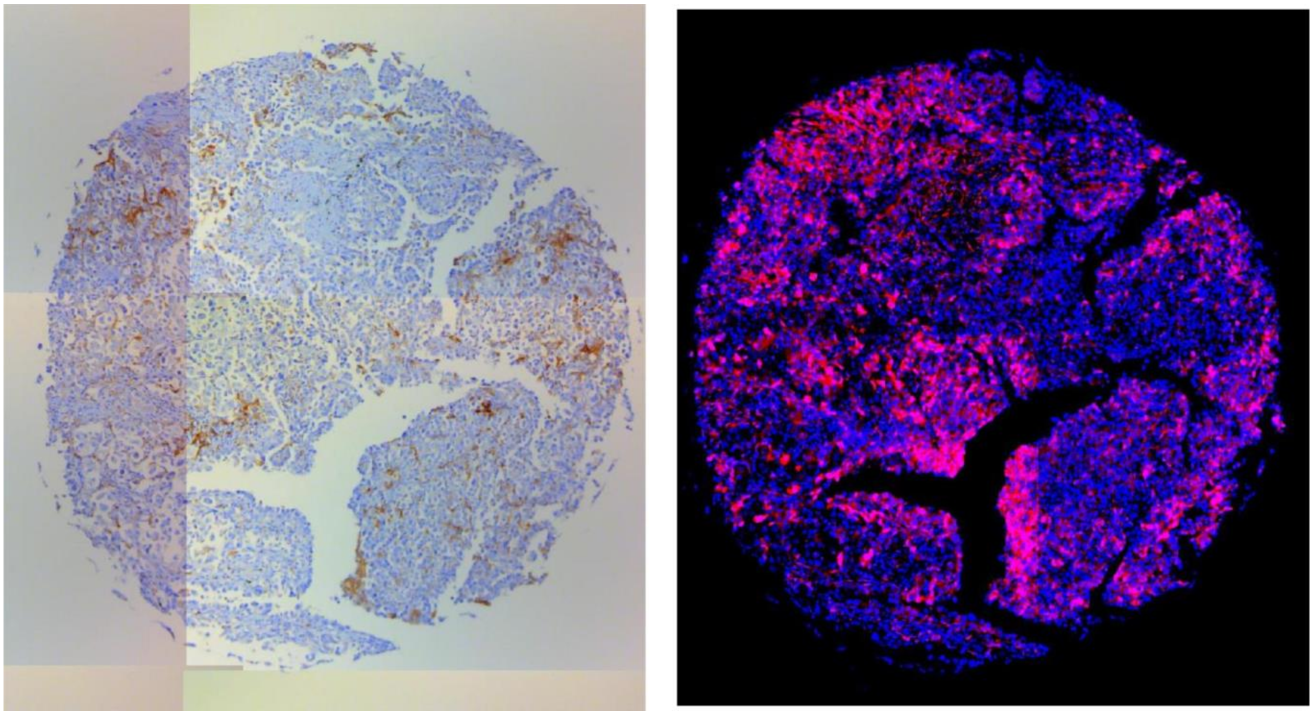

Figure S56. TMA Stained with SP263 (left) and RK-10-Cy5 (right) shows negative signal from SP263 and positive stain using the RK-10-Cy5 peptide.

S57: Peptide Positive, SP263 Negative

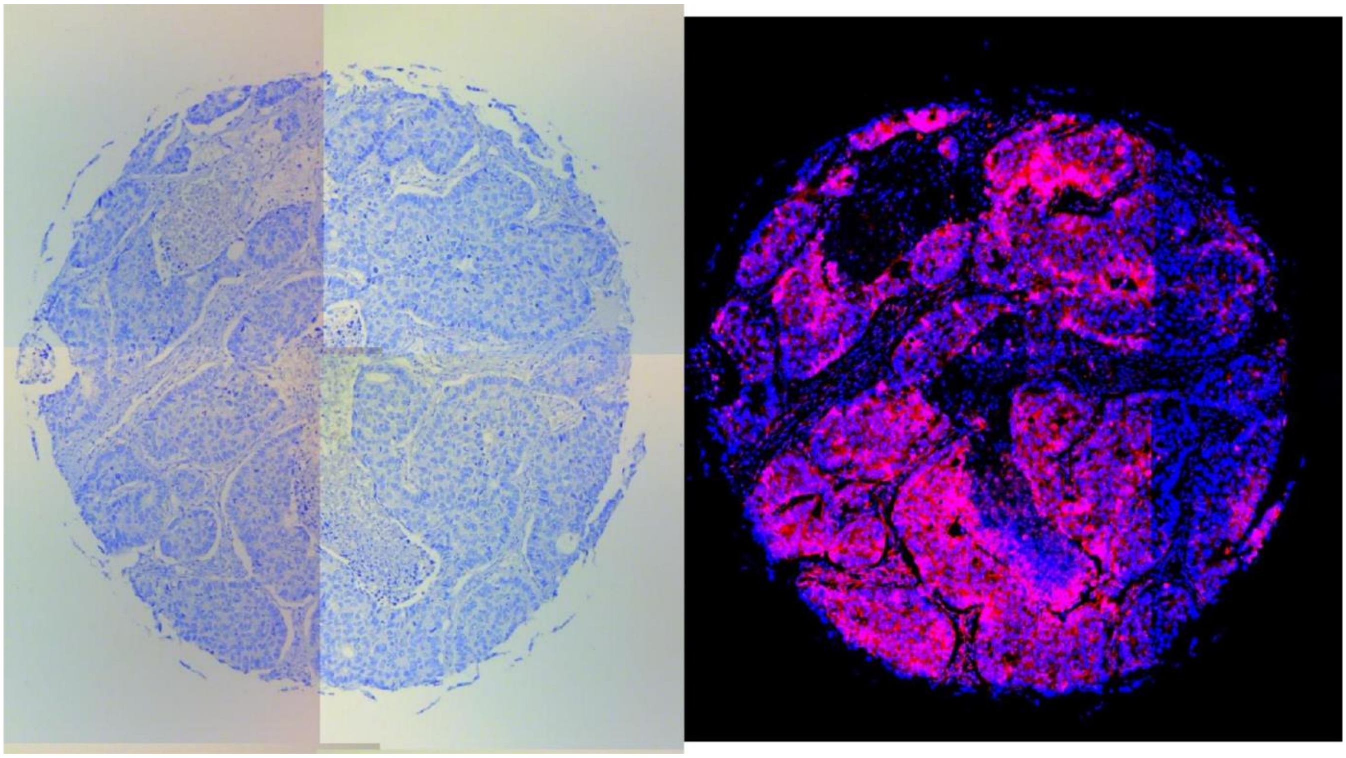

Figure S57. TMA Stained with SP263 (left) and RK-10-Cy5 (right) shows negative signal from SP263 and positive stain using the RK-10-Cy5 peptide.

S58: Peptide Positive, SP263 Negative

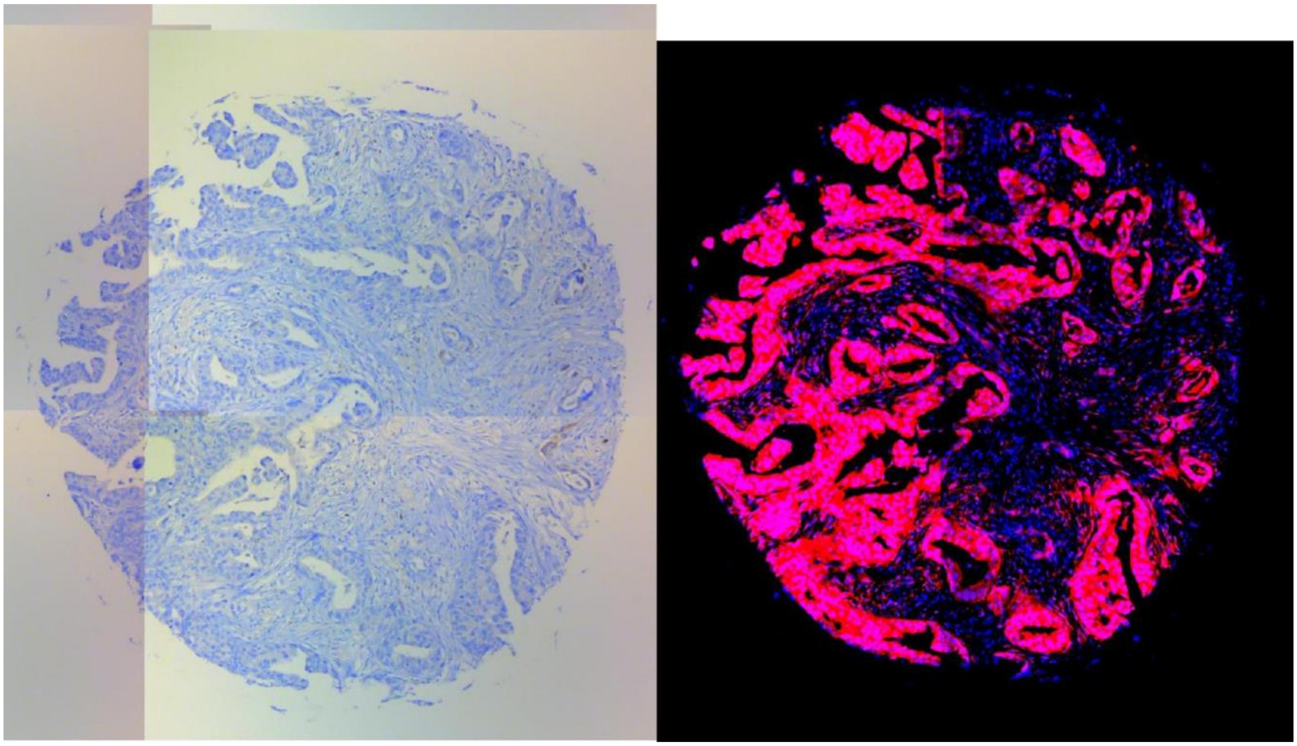

Figure S58. TMA Stained with SP263 (left) and RK-10-Cy5 (right) shows negative signal from SP263 and positive stain using the RK-10-Cy5 peptide.

S59: Peptide Positive, SP263 Negative

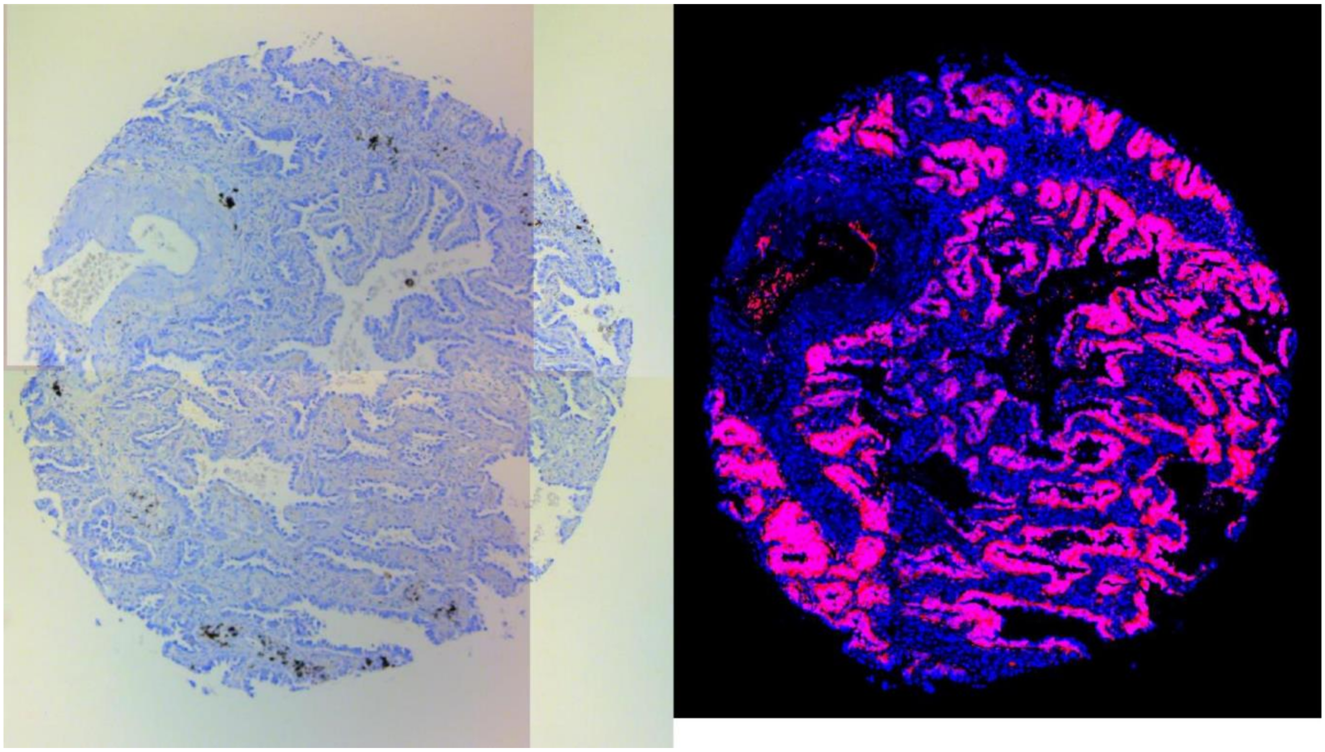

Figure S59. TMA Stained with SP263 (left) and RK-10-Cy5 (right) shows negative signal from SP263 and positive stain using the RK-10-Cy5 peptide.

S60: Peptide Positive, SP263 Negative

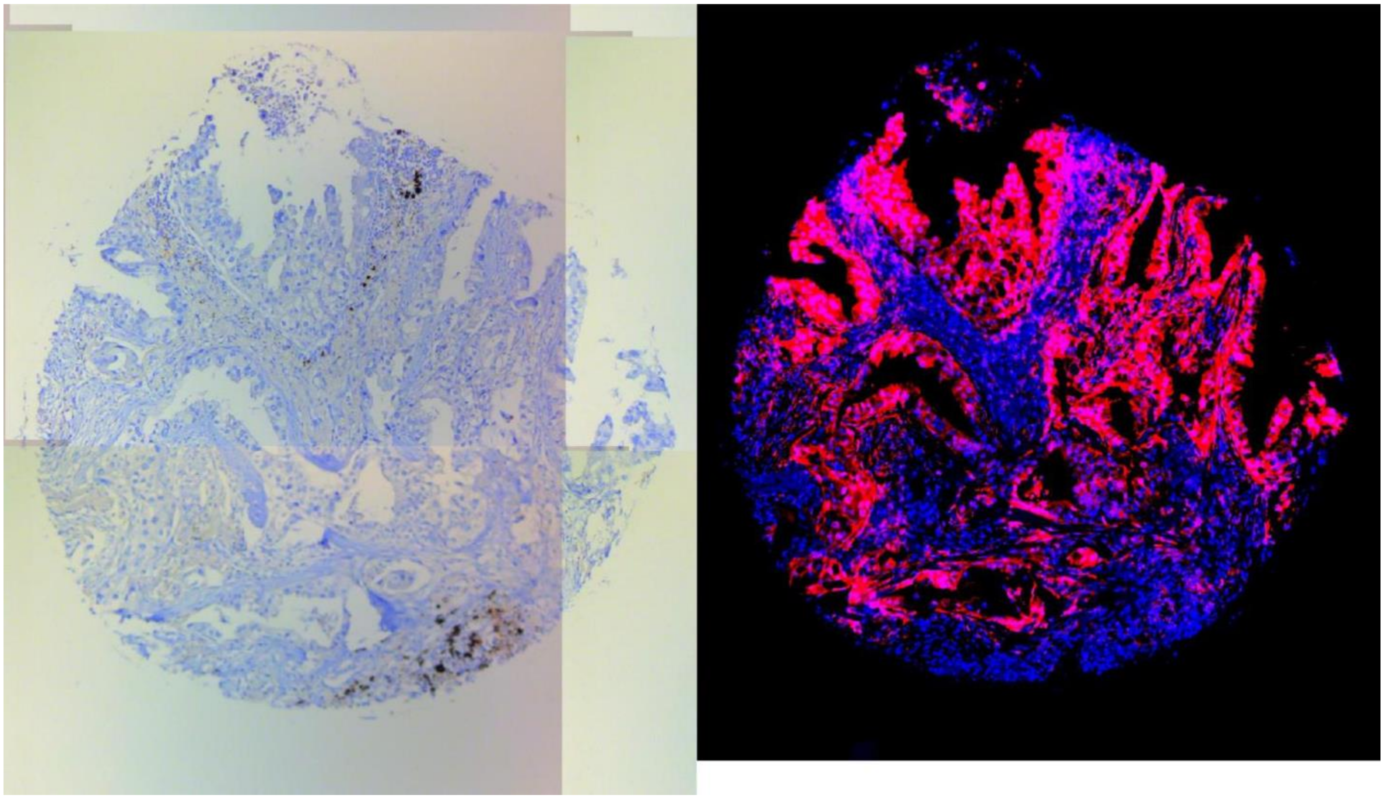

Figure S60. TMA Stained with SP263 (left) and RK-10-Cy5 (right) shows negative signal from SP263 and positive stain using the RK-10-Cy5 peptide.

S61: Peptide Positive, SP263 Negative

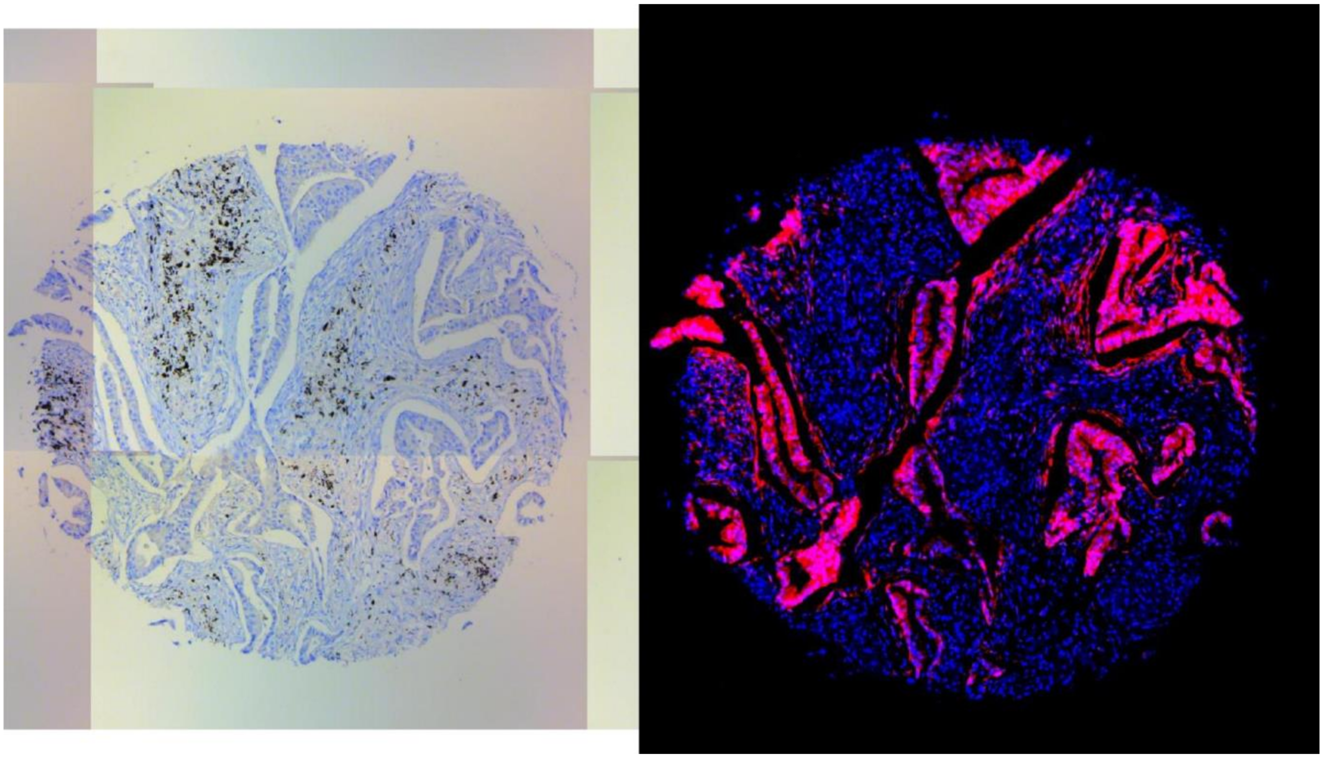

Figure S61. TMA Stained with SP263 (left) and RK-10-Cy5 (right) shows negative signal from SP263 and positive stain using the RK-10-Cy5 peptide.

S62: Peptide Positive, SP263 Negative

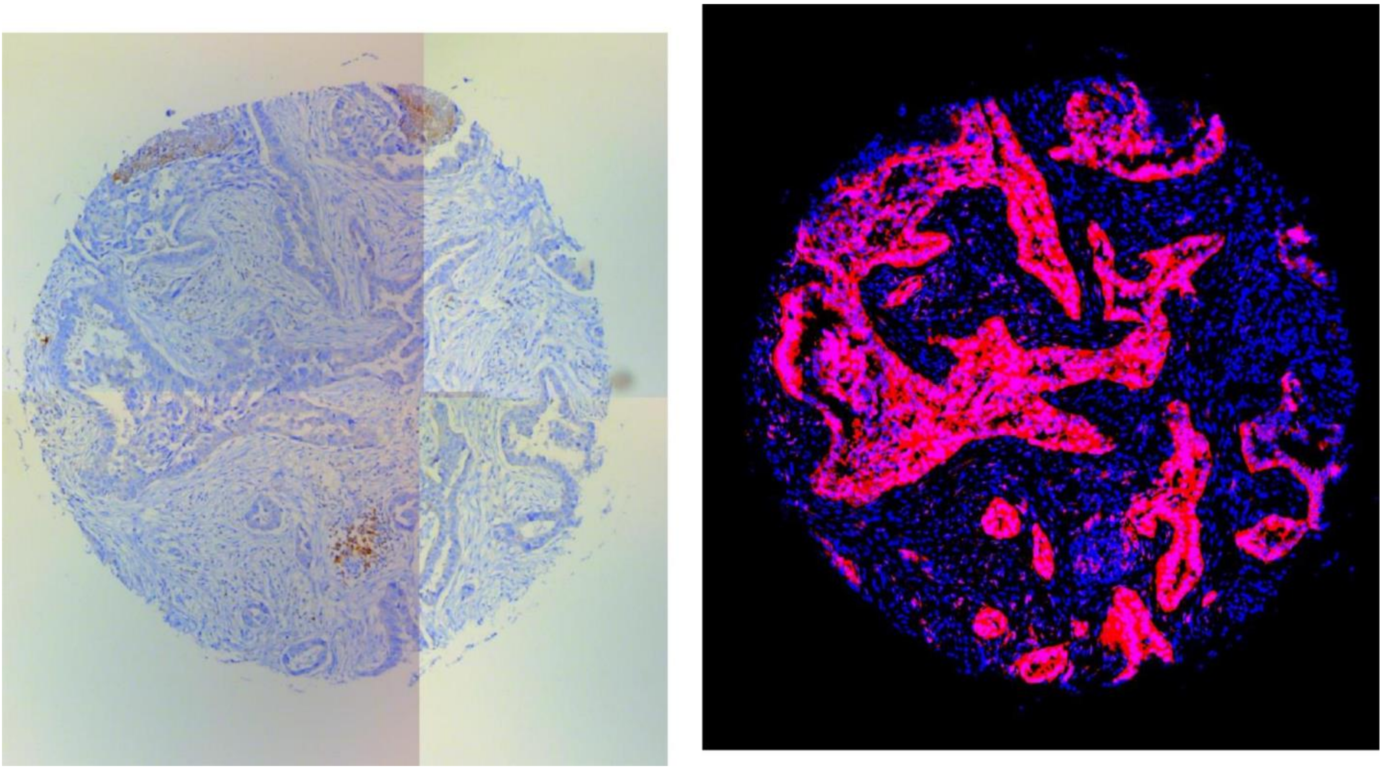

Figure S62. TMA Stained with SP263 (left) and RK-10-Cy5 (right) shows negative signal from SP263 and positive stain using the RK-10-Cy5 peptide.

S63: Peptide Positive, SP263 Negative

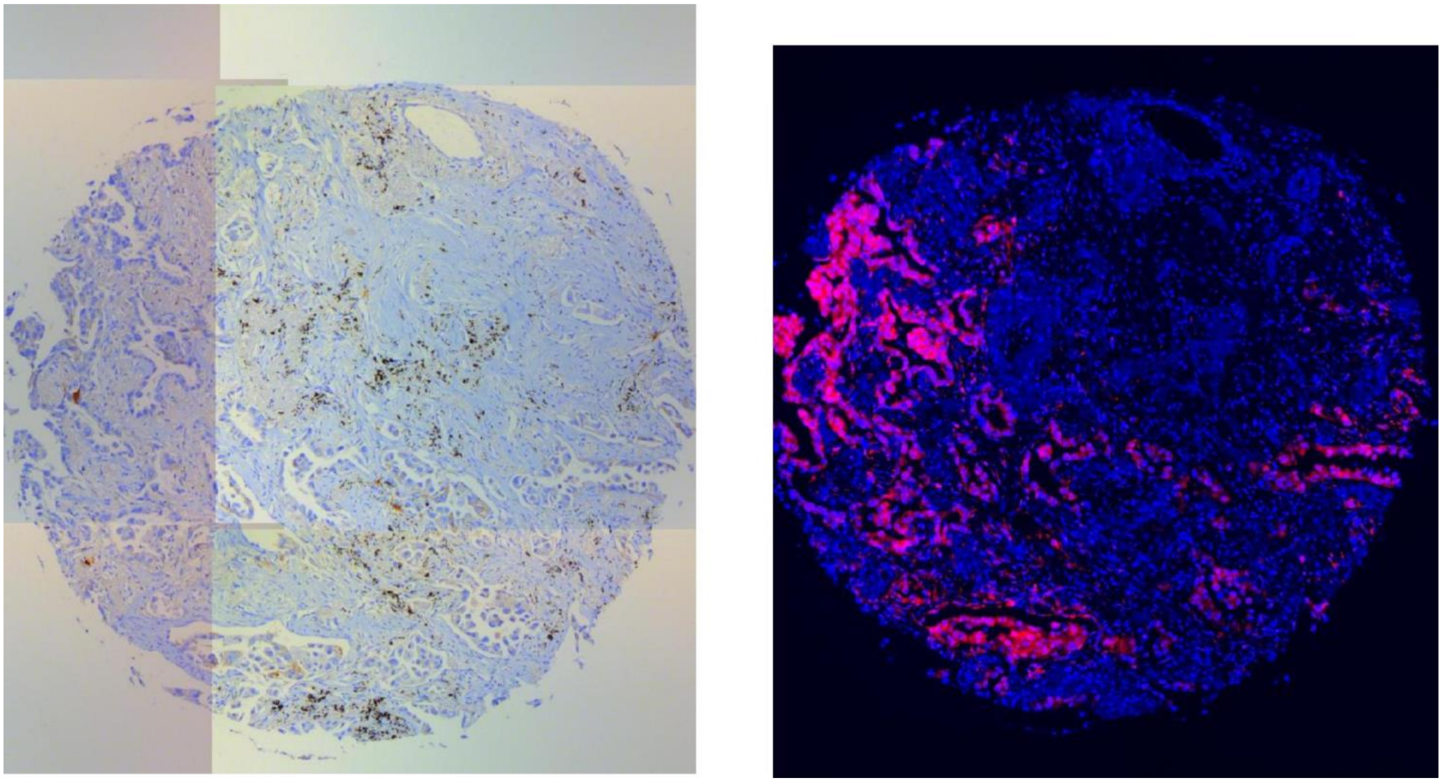

Figure S63. TMA Stained with SP263 (left) and RK-10-Cy5 (right) shows negative signal from SP263 and positive stain using the RK-10-Cy5 peptide.

S64: Peptide Positive, SP263 Negative

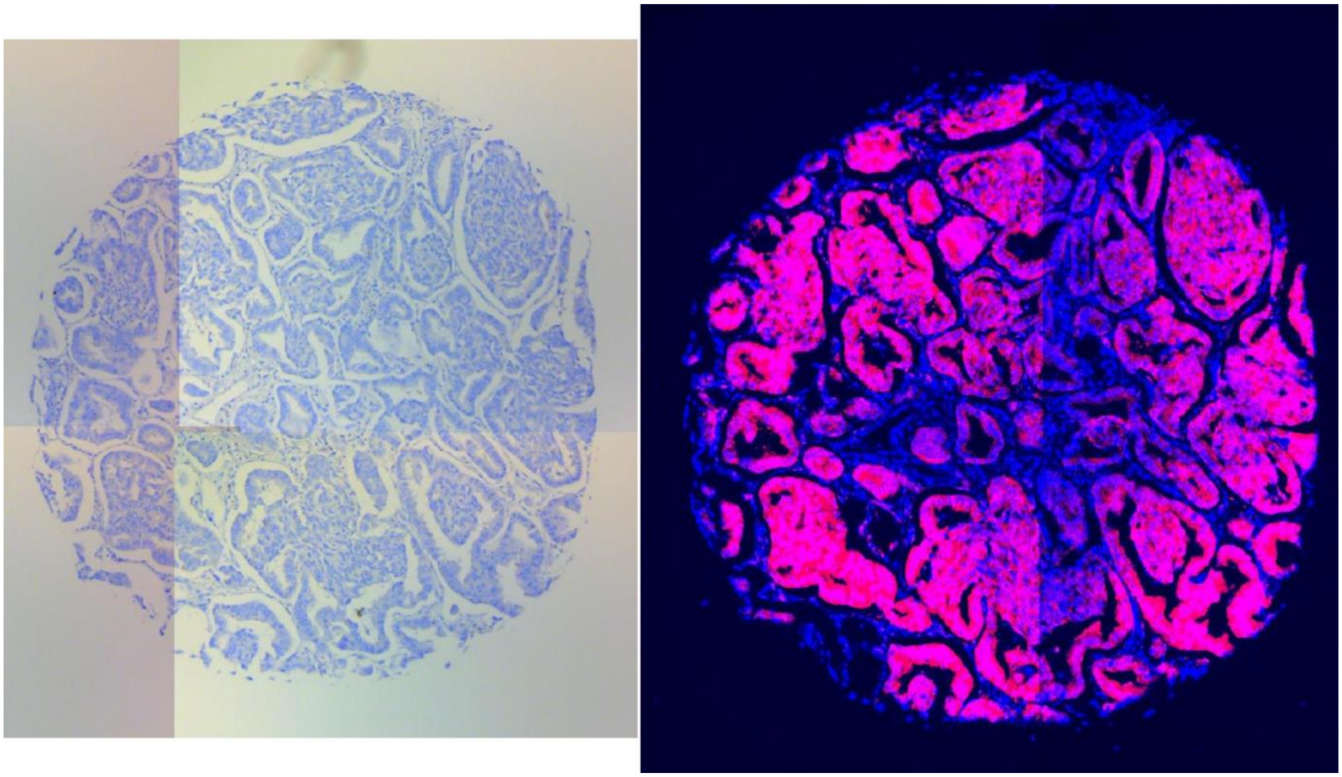

Figure S64. TMA Stained with SP263 (left) and RK-10-Cy5 (right) shows negative signal from SP263 and positive stain using the RK-10-Cy5 peptide.

S65: Peptide Positive, SP263 Negative

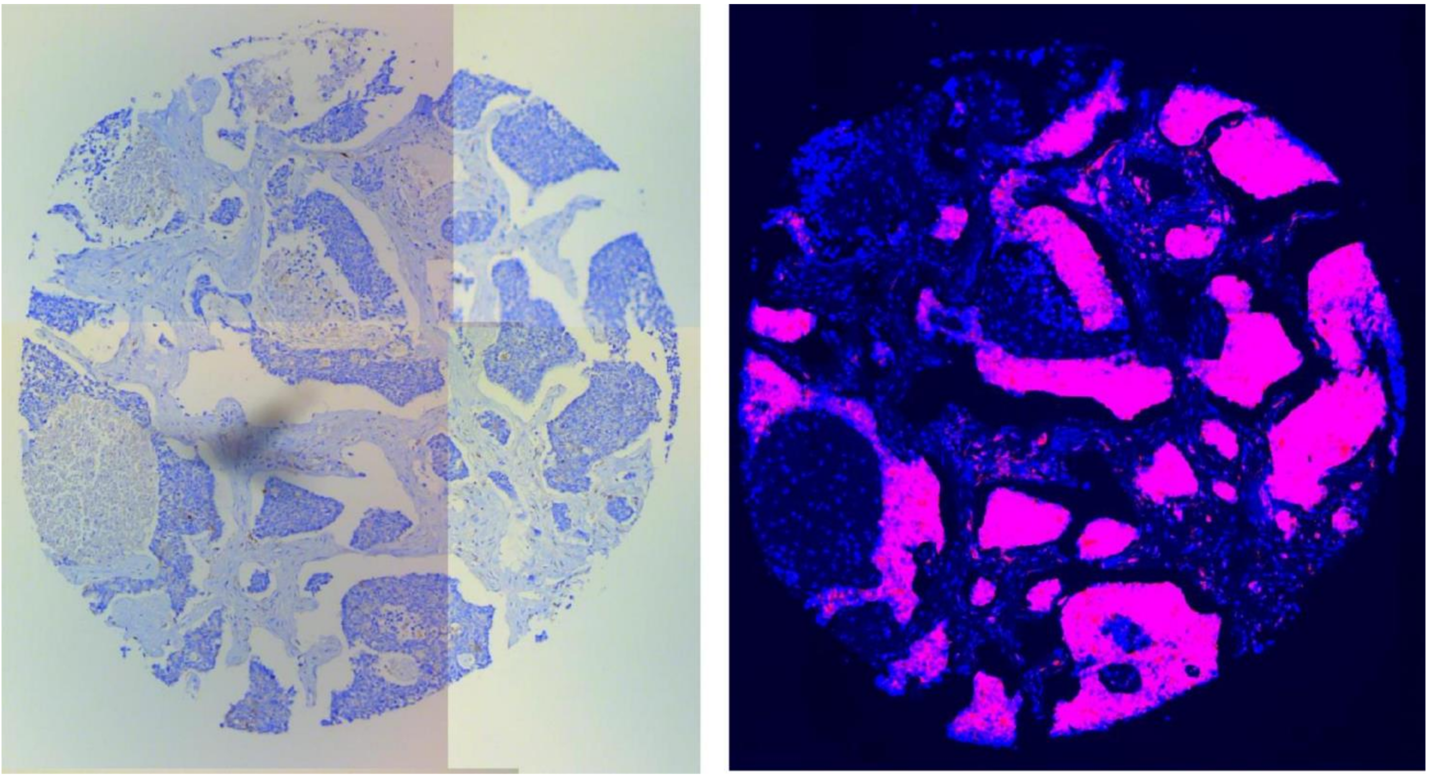

Figure S65. TMA Stained with SP263 (left) and RK-10-Cy5 (right) shows negative signal from SP263 and positive stain using the RK-10-Cy5 peptide.
